# Exposure of Polycyclic Aromatic Hydrocarbons (PAHs) and Crude Oil to Atlantic Haddock (*Melanogrammus aeglefinus*): A Unique Snapshot of the Mercapturic Acid Pathway

**DOI:** 10.1021/acs.est.4c05112

**Published:** 2024-08-05

**Authors:** Charlotte L. Nakken, Marc H. G. Berntssen, Sonnich Meier, Lubertus Bijlsma, Svein A. Mjøs, Elin Sørhus, Carey E. Donald

**Affiliations:** † Department of Chemistry, University of Bergen, Bergen 5007, Norway; ‡ Marine Toxicology, Institute of Marine Research, Bergen 5817, Norway; § Environmental and Public Health Analytical Chemistry, Research Institute for Pesticides and Water, University Jaume I, Castellón 12071, Spain

**Keywords:** Bile metabolites, fish, high-resolution mass spectrometry, crude oil, screening, xenobiotic

## Abstract

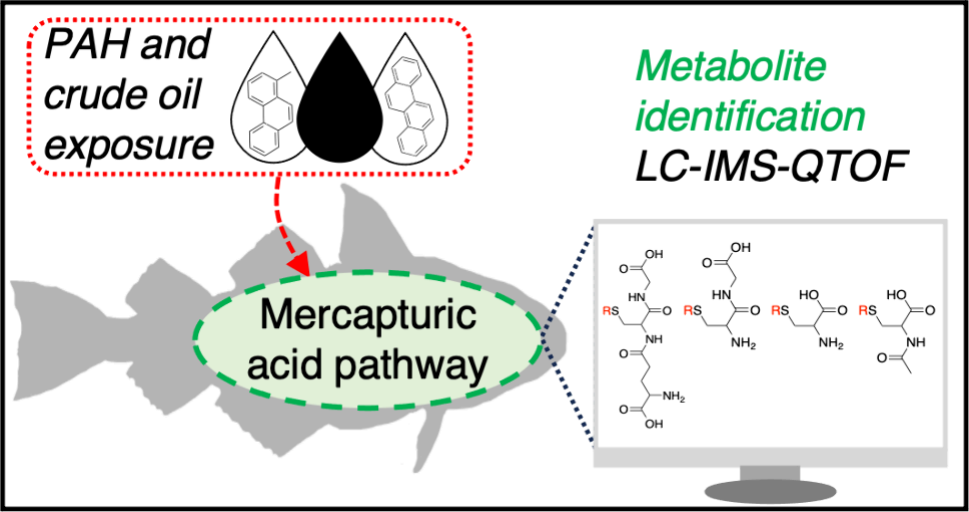

Fish exposed to xenobiotics like petroleum-derived polycyclic aromatic hydrocarbons (PAHs) will immediately initiate detoxification systems through effective biotransformation reactions. Yet, there is a discrepancy between recognized metabolic pathways and the actual metabolites detected in fish following PAH exposure like oil pollution. To deepen our understanding of PAH detoxification, we conducted experiments exposing Atlantic haddock (*Melanogrammus aeglefinus*) to individual PAHs or complex oil mixtures. Bile extracts, analyzed by using an ion mobility quadrupole time-of-flight mass spectrometer, revealed novel metabolites associated with the mercapturic acid pathway. A dominant spectral feature recognized as PAH thiols set the basis for a screening strategy targeting (i) glutathione-, (ii) cysteinylglycine-, (iii) cysteine-, and (iv) mercapturic acid S-conjugates. Based on controlled single-exposure experiments, we constructed an interactive library of 33 metabolites originating from 8 PAHs (anthracene, phenanthrene, 1-methylphenanthrene, 1,4-dimethylphenanthrene, chrysene, benz[*a*]anthracene, benzo[*a*]pyrene, and dibenz[*a*,*h*]anthracene). By incorporation of the library in the analysis of samples from crude oil exposed fish, PAHs conjugated with glutathione and cysteinylglycine were uncovered. This qualitative study offers an exclusive glimpse into the rarely acknowledged mercapturic acid detoxification pathway in fish. Furthermore, this furnishes evidence that this metabolic pathway also succeeds for PAHs in complex pollution sources, a notable discovery not previously reported.

## Abstract 2

Laboratory studies on Atlantic haddock present new insights into the detoxification of polycyclic aromatic hydrocarbons by the mercapturic acid pathway.

## Introduction

Fish in the vicinity of offshore oil and gas production are constantly exposed to emissions, more specifically produced water (PW) which represents dispersed crude oil and the petroleum constituents polycyclic aromatic hydrocarbons (PAHs).^1−3^ To facilitate the elimination of PAHs, a series of Phase I and II reactions are initiated to enhance their polarity and aid excretion (Phase III). The primary Phase I reaction is mediated by the cytochrome P450 (CYP) enzymes to produce oxygenated metabolites.^4^ Further metabolic action is typically performed by Phase II biotransformations, which involve covalent bonding to large polar groups, predominantly sulfate, glucuronic acid, and glutathione (**GSH**) conjugates.^5−8^ These biotransformation processes primarily occur in the liver. Due to the subsequent storage of detoxification products in the gall bladder, biliary metabolite analysis have become a well-established biomarker of recent PAH exposure in fish.^9^ Thus, mapping the bile metabolites becomes crucial in assessing PAH exposure, as it provides a snapshot of PAHs prior to excretion. However, since these analyses have typically focused on hydroxylated metabolites along with deconjugated sulfate and glucuronide forms,^10^ little emphasis has been given to other metabolic products, including the **GSH** compounds.

**Figure 1:**
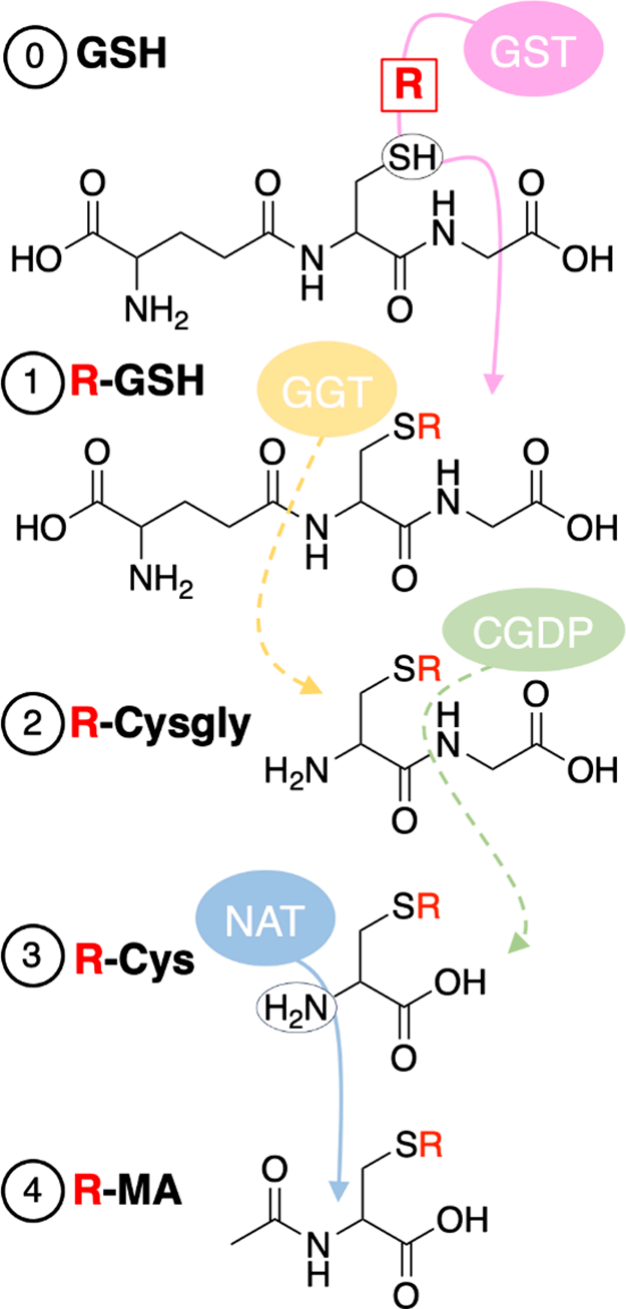
Proposed conjugates in the mercapturic acid pathway (**MAP**): (1) Glutathione (**GSH**), (2) Cysteinylglycine (**cysgly**), (3) Cysteine (**cys**), and (4) *N*-acetyl-l-cysteine (Mercapturic acid, **MA**). R = xenobiotic, GST = glutathione S-transferase, GGT = gamma-glutamyl transferase, CGDP = cysteinylglycine dipeptidase, NAT = *N*-acetyltransferase.^14^

Conjugation to the tripeptide **GSH** is initiated by a nucleophilic addition reaction with an electrophilic xenobiotic, like a Phase I PAH diol epoxide produced by CYP.^11^ Glutathione *S*-transferases (GST) typically catalyze this process (0, Figure 1), though the reaction can also occur spontaneously.^12^**GSH** can further enter the major biotransformation pathway known as the mercapturic acid pathway (**MAP**),^13^ which consists of four different conjugates: (1) **GSH**, (2) Cysteinylglycine (**cysgly**), (3) Cysteine (**cys**), and (4) *N*-acetyl-l-cysteine (Mercapturic acid, **MA**).^13,14^ Briefly, the cleavage of glutamic acid by gamma-glutamyl transferase (GGT) forms the second conjugate, **cysgly**. Next, cleavage of the glycine moiety by cysteinylglycine dipeptidase (CGDP) results in the amino acid **cys**. Acetylation catalyzed by *N*-acetyltransferase (NAT) leaves the ultimate conjugate, **MA.**(14,15)

Research in mammals indicates that the **MAP** is a central **GSH**-mediated metabolization pathway for PAHs as they can form various metabolites in the **MAP** across multiple biological contexts, encompassing human research, rodents, and cell studies.^16−21^ While glucuronide and sulfate metabolites are the main focus of Phase II pathways investigated in fish, **GSH**-derived metabolites have been comparatively less investigated.^10^ GST activity can serve as a biomarker to assess the effects of PAHs in fish,^22^ indicating that **GSH** plays a role in PAH detoxification. The tentative identifications of **GSH** metabolites from benzo[a]pyrene in English sole and starry flounder supports this conclusion.^23−25^ Still, our current knowledge base suggests limited insight into the potential transformation of **GSH** conjugates into **cysgly**, **cys**, or **MA** conjugates via the **MAP** in PAH exposed fish and their detection using mass spectrometry.

In this work, analysis by ion mobility combined with high-resolution mass spectrometry (IM-HRMS), aimed at mapping the conventional metabolites in fish bile, revealed numerous unexpected metabolites originating from the **MAP**. Based on our data set from individual PAH exposed fish, we developed a method to establish a **MAP** metabolite library designed for application in suspect screening analyses of fish exposed to complex oil mixtures. These metabolites are presented in an interactive library along with a modified nontarget screening strategy. The qualitative metabolite analysis herein contributes to improved insight into PAH metabolism and presents **MAP** metabolites not previously reported from oil-exposed fish. Hence, these newly identified products should be included in future investigations to better understand the impact of chronic emissions from oil production.

## Methods

### Chemicals

An instrument calibration solution (CCS Major Mix) and leucine enkephalin, used as the lock mass, were purchased from Waters (Manchester, U.K.). HPLC- and UHPLC-grade solvents (Chromasolv) were obtained from Honeywell (Seelze, Germany). Phenanthrene (CAS 85–01–8), dimethyl sulfoxide (DMSO), and tricaine methanesulfonate (MS-222) were purchased from Sigma-Aldrich (Oslo, Norway). 1-Methylphenanthrene (CAS 832–69–9), 1,4-dimethylphenanthrene (CAS 22349–59–3), benz[a]anthracene (CAS 56–55–3), chrysene (CAS 218–01–9), benzo[a]pyrene (CAS 50–32–8), and dibenz[a,h]anthracene (CAS 53–70–3) were purchased from Chiron (Trondheim, Norway). 1-Methylnaphtalene (CAS 90–12–0), 1,4-dimethylnaphtalene (CAS 571–58–4), anthracene (CAS 120–12–7), and 1-methylpyrene (CAS 2381–21–7) were purchased from LGC Standards AB (UK).

Two different complex petroleum related PAH mixtures were designed to represent the PAH composition in heavy weathered crude oil and production water (PW). Details about the composition and the making of these mixtures are given in Meier et al.^26^ The two petroleum mixtures contained significant quantities of various oil compounds, with PAHs contributing 1.5% (crude oil) and 1.1% (PW) of the weight. The PAH profile (% of total PAHs) in the weathered crude oil was dominated by 3-ring PAHs (93%), some 4-ring PAHs (5.3%) and 5-ring PAHs (1%). The PW-mixture was dominated by 2-ring PAHs (77%), with some 3-rings PAHs (22%) and trace amounts of heavy PAHs (>0.6% of 4–5 ring PAHs). The weathered crude oil was prepared using 45% of Gullfaks oil (Norwegian North Sea, Tampen area) distillation fractions (boiling points: 320–375 °C) and 55% distillation fractions (boiling points: 375–400 °C) with the addition of 0.06% pyrene. The PW-mixture was prepared using 90% of a cyclo-hexane extract of PW from Statfjord A (Norwegian North Sea, Tampen area) and 10% of distillation fractions (boiling points: 240–320 °C) of Gullfaks oil.

### Fish Exposure and Bile Sample Preparation

The study was performed using Atlantic haddock (*Melanogrammus aeglefinus*) as it is an important ecological and commercial species with habitat in areas with chronic emissions of PAHs in the North Sea.^2^ Exposure experiments were performed similarly as described in Meier et al.,^26^ with adult haddock supplied from a brood stock at the Austevoll Research Station. All animal experiments were approved (FOTS ID 5924) by the Norwegian Animal Research Authority. All methods were performed in accordance with the approved guidelines. The Austevoll Research station has the following permission for the catch and maintenance of Atlantic haddock: H-AV 77, H-AV 78, and H-AV 79 (given by the Norwegian Directorate of Fisheries). The Austevoll Research station has a permit to run as a Research Animal facility using fish (all developmental stages) with code 93.

To establish the proof of concept for PAH detoxification, the study utilized injected exposure concentrations that would ensure the detection of all potential PAH metabolites. The study included 14 treatments representing a suit of petroleum relevant PAHs or complex oil mixtures: 5 alkylated PAHs, 6 unsubstituted PAHs, 2 complex oil mixtures, and a control. Fish were exposed to a high dose of an individual PAH or alkylated PAH compound dissolved in DMSO and fish oil corresponding to a dose of 5 mg/kg fish by intraperitoneal injection (number of bile extracts from individually exposed fish in brackets): 1-methylnaphthalene (2), 1,4-dimethylnaphtalene (1), anthracene (2), phenanthrene (3), 1-methylphenanthrene (2), 1,4-dimethylphenanthrene (2), chrysene (2), benz[a]anthracene (2), 1-methylpyrene (2), benzo[a]pyrene (2), dibenz[a,h]anthracene (2). The control treatment (1) had no PAHs administered. The two complex oil mixtures, crude oil (4) and PW (4), were injected directly without DMSO or fish oil. The exposure doses were 7.4 ± 0.3 mg PAH/kg fish for crude oil and 5.3 ± 0.3 mg PAH/kg fish for PW. Fish were anesthetized before injection (60 mg/L MS-222).

The fish were euthanized 3 days postinjection using a high dose of anesthetic. Bile was kept frozen in liquid nitrogen and stored at −80 °C until metabolite extraction following the protocol of da Silva et al.^27^ and was later stored in the dark at −20 °C until metabolite analysis. In brief, the sample preparation involved methanol extraction, including phospholipid removal, followed by hydrolysis with glucuronidase and sulfatase enzyme (pH 5, 1 h, 40 °C), and solid phase extraction. Each bile extract represents one fish, exposed to one PAH or mixture treatment. Two bile samples had less than the desired bile volume (<50 uL; one from each of the treatments 1-methylnaphthalene and chrysene) and were therefore discarded.

### Instrumentation

Analyses were performed on a Waters Acquity UPLC system interfaced to an ion mobility quadrupole time-of-flight mass spectrometer (LC-IM-QTOF MS) (Waters, Milford, MA, USA). The reverse phase C18 ACQUITY UPLC BEH column (Waters, 100 × 2.1 mm, 1.7 μm) was kept at 45 °C. A binary mobile phase, composed by methanol (B) and water (A), was employed at a flow rate of 0.45 mL/min following the gradient program: 0.0 min, 30% B; 0.1 min, 30% B; 20.10 min, 80% B; 20.2 min, 98% B; 24.0 min, 98% B, 24.1 min, 30% B. The column equilibrated until 29.0 min. The sample manager was set at 10 °C, and the injection volume was 5 μL.

The LockSpray electrospray ionization was set at 2.6 kV in negative ionization mode; cone voltage of 40 V; desolvation temperature of 475 °C; source temperature of 110 °C; desolvation gas flow (N_2_) of 950 L/h; and cone gas flow (N_2_) of 50 L/h. Automatic lock mass correction (leucine enkephalin; *m*/*z* 554.2615) was used for accurate mass correction at half-minutes intervals. The analyzer mode was set to sensitivity, and the acquisition mode was in high definition MS^E^. A scan time of 0.3 s was set in the range of *m*/*z* 50–800. The low energy scan was set at 6 eV to monitor the deprotonated molecules, while the high energy ramp (8–45 eV) monitored their corresponding fragment ions. Nitrogen was employed as drift gas and argon as collision-induced dissociation gas.

Originally, samples from exposures to single compounds were analyzed. A year later, screening of the bile extracts collected from the crude oil and PW exposed fish were performed using the new established library.

### Data Processing and Library Building

**Figure 2:**
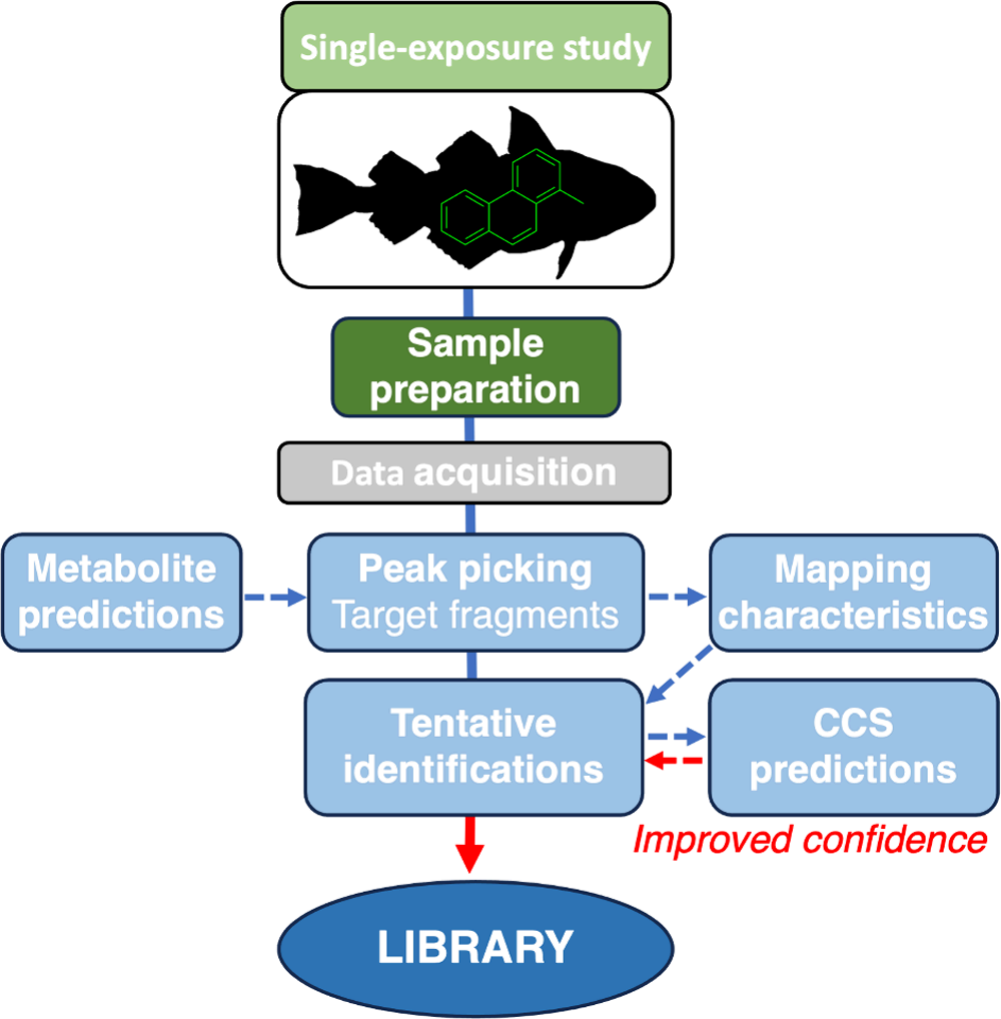
Schematic overview of the experimental workflow for constructing a custom library of novel metabolites, based on controlled single-exposure experiments and data processing with improved confidence from predicted collision cross section (CCS) values (adapted from Bijlsma et al.^28^).

Data acquisition and processing were performed in UNIFI software (version 1.9.4.053, Waters). To enhance the data processing and reduce the number of false positives, the retention time and mass were constrained by the detection information on the heaviest and least polar PAH in the study. Accordingly, based on the detection information on dibenz[a,h]anthracene, data filtering and processing were focusing on compounds (<700 Da) eluting within 10 min. Intensity thresholds for low and high energy ions were set at 5 and maximum candidates (i.e., detected *m*/*z*) to keep for screening was set to 15 000 per sample. As presented in Figure 2, the experimental design involves a modified version of the refined nontarget workflow by Bijlsma et al.^28^ The single-exposure study and the sample preparation are described in the section Fish Exposure and Bile Sample Preparation.

Following the acquisition of the bile extracts, several data processing steps were carried out. First, a filter targeting the suspected base peaks in the spectra, i.e. [M+S–H]^−^ and [M+S+H_2_O_2_–H]^−^, corresponding to a PAH-thiol and a dihydroxy-dihydro-PAH-thiol, was applied for peak picking. Concurrently, metabolites from **MAP** were explored using the transformation tool in UNIFI for predicting potential biotransformation products of the treatment compound. Phase I transformations included oxidation (+O), double oxidation (+O_2_), and dihydrodiol formation (+H_2_O_2_). Phase II transformations consisted of conjugation with **GSH** (+C_10_H_15_N_3_O_6_S, + C_10_H_15_N_3_O_5_S), **cysgly** (+C_5_H_8_N_2_O_3_S), **MA** (+C_5_H_7_NO_3_S), and **cys** (+C_3_H_5_NO_2_S, + C_3_H_5_NOS). The maximum number of transformations was set at two Phase I and one Phase II transformations.

Peaks matching both the mass of a biotransformation product and a target fragment ion were manually investigated for common characteristics, such as common neutral losses (CNLs) and common fragment ions. Several fragments and CNLs were noted but were excluded from the method due to a lack of specificity. This same procedure was applied to all single compound samples, resulting in the CNLs and fragments presented in the results (Table 1). These characteristics were incorporated into an in-house-built accurate mass screening workflow within the UNIFI software to facilitate tentative identifications. The chemical structures of two potential isomers fitting the accurate mass were drawn in ChemDraw (version 20.1.0.112) and uploaded to the UNIFI software to aid in fragment elucidation. Collision cross section (CCS) predictions for additional identification confidence were performed by the CCS_H-_ model presented by Celma et al.^29^ Prediction accuracy for the CCS_H-_ model was ±5.86% (relative error within the 95% confidence interval).

**Table 1:** Key Fragment Ions (*m/z*) and Common Neutral Losses (Monoisotopic Mass) Employed for Selective Screening of Metabolites in the Mercapturic Acid Pathway (**MAP**) in Negative Ionization Mode

Conjugate	Fragment ion (*m*/*z*)	Common neutral loss (Da)
*Glutathione (****GSH****)*	C_10_H_14_N_3_O_6_ (272.08881)	C_10_H_19_N_3_O_7_S (325.09437)
		C_10_H_17_N_3_O_7_ (291.10665)
		C_10_H_16_N_3_O_6_ (274.10391)
		C_10_H_15_N_3_O_6_ (273.09609)
		C_5_H_10_N_2_O_4_ (162.06406)
		C_5_H_10_N_2_O_3_ (146.06914)
*Cysteinylglycine (****cysgly****)*	C_5_H_9_N_2_O_3_S (177.03394)	C_5_H_12_N_2_O_4_S (196.05178)
	C_5_H_7_N_2_O_3_ (143.04622)	C_5_H_10_N_2_O_4_ (162.06406)
		C_5_H_8_N_2_O_3_ (144.05349)
*Cysteine (****cys****)*	C_5_H_4_NO_2_S (141.99682)	C_3_H_9_NO_5_ (139.04807)
		C_3_H_5_NO_2_ (87.03203)
*N-acetyl-*l*-cysteine (Mercapturic acid*; ***MA****)*	C_5_H_6_NO_3_ (128.03532)	C_5_H_7_NO_3_ (129.04259)

Due to the lack of available reference standards for any of the reported metabolites, we placed a high priority on reducing the risk of false positives and gaining the highest possible confidence in our identifications. Hence, we implemented strict identification criteria for inclusion in our custom scientific library in UNIFI: (i) mass accuracy ≤ 5 ppm for the suspect ion, (ii) a characteristic CNL, (iii) a characteristic conjugate fragment ion, (iv) CCS deviation within model limits, and (v) not detected in the control. Criteria (i)-iv) were automated in the data processing by using an accurate mass screening workflow in UNIFI. The custom library serves as a repository of detection information (*m*/*z*, fragment ions, retention time, CCS, spectra). Afterward, it can be imported and implemented in data processing in other batches, as demonstrated in our case with crude oil and PW samples.

All identified metabolites were collected in a library, presented here as interactive files in the Supporting Information. The library (.html and .pdf) presents the following information: (1) Structural information: formula, neutral mass, predicted CCS, suggested chemical structure, and simplified molecular-input line-entry system (SMILES), 2) Detection information: observed *m*/*z*, retention time, CCS, delta difference for acquired vs predicted CCS, adduct, and low- and high energy mass spectra. The exact position of the substitutions and conjugates are indeterminable. Compounds with the same conjugation were assigned numerical names with increasing number Phase I substitutions (e.g., cysteinylglycine I, II, and III). If isomers were detected, the library comprised the two largest peak areas, denoted as A and B, respectively. Detection information (*m*/*z*, retention time, and CCS) is reported in Table S1. Due to the lack of analytical standards and overall dose-dependent enzymatic deconjugation of most newly described metabolic pathways, the results are qualitative rather than quantitative.

## Results and Discussion

### Bile Extracts Captured Unique Insight into the Mercapturic Acid Pathway

Implementation of the screening strategy herein led to the detection of 33 bile metabolites involving all four conjugates in the **MAP**: (1) **GSH**, (2) **cysgly**, (3) **cys**, and (4) **MA** (Figure 1). Metabolites were identified in the following treatments: dibenz[*a*,*h*]anthracene, benzo[*a*]pyrene, chrysene, benz[*a*]anthracene, 1,4-dimethylphenanthrene, 1-methylphenanthrene, phenanthrene, and anthracene. Detection information and suggested structural details are presented in the library in the Supporting Information. No identifications were performed in bile extracts from the treatment groups 1-methylnaphthalene, 1,4-dimethylnaphtalene or 1-methylpyrene.

**Figure 3:**
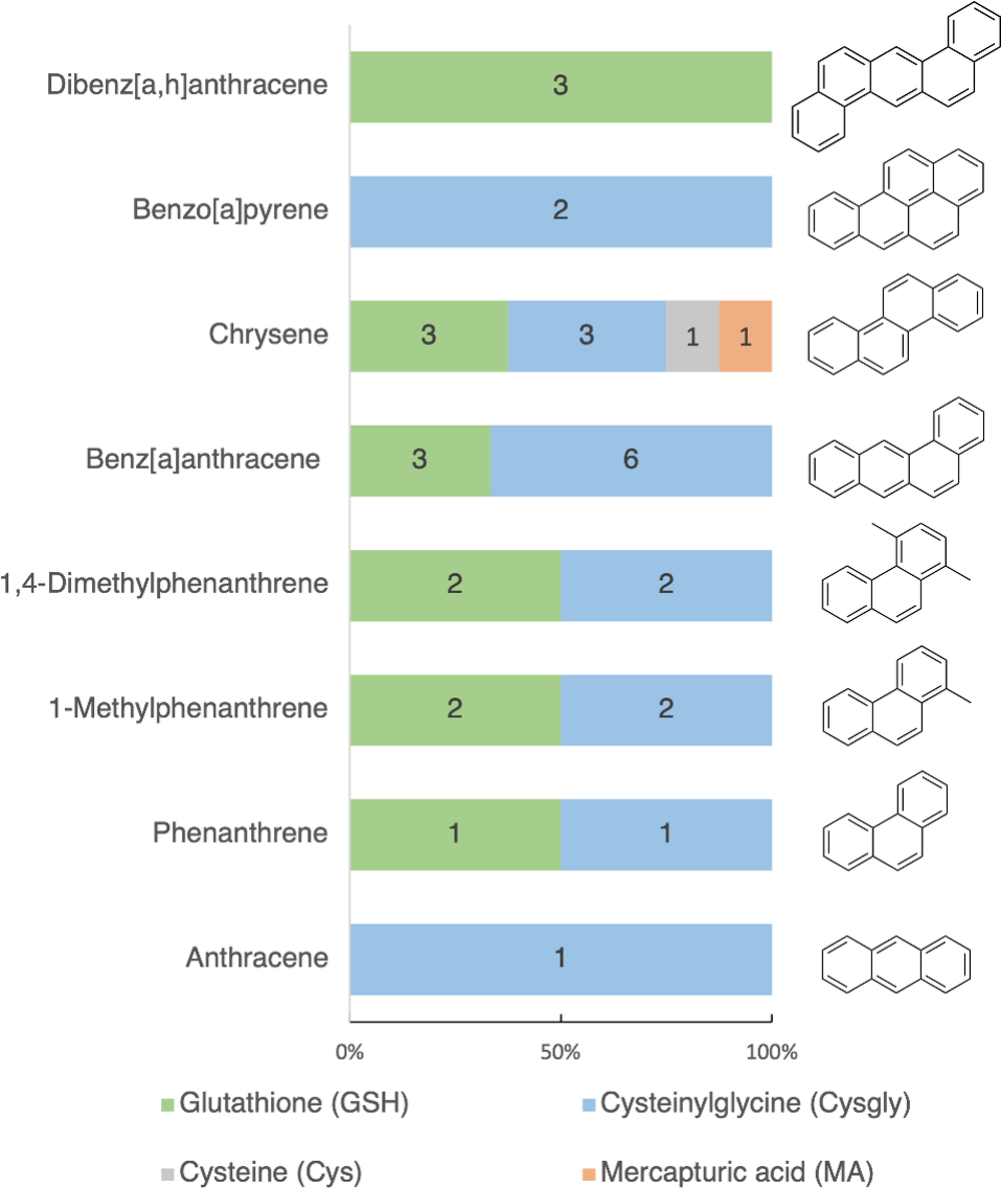
Graphical presentation of number metabolites from the mercapturic acid pathway (**MAP**) detected in the treatment groups dibenz[*a*,*h*]anthracene, benzo[*a*]pyrene, chrysene, benz[*a*]anthracene, 1,4-dimethylphenanthrene, 1-methylphenanthrene, phenanthrene, and anthracene.

A visual overview of the 33 identified metabolites across the single-exposure treatments is provided in Figure 3. **GSH** and **cysgly** represent the first and second steps of the pathway and were notably the most commonly observed metabolite types overall.

The results from the benzo[*a*]pyrene treatment are of particular interest since it is a well-studied carcinogen known to produce highly reactive metabolites that can covalently bond to DNA, i.e. form DNA-adducts.^30,31^ The presence of DNA adducts provides an indication of previous exposure and the potential induction of carcinogenesis.^32^**GSH** can participate in conjugation reactions with benzo(a)pyrene metabolites and thus prevents potential DNA damage by reducing the available reactive species.^33^ In fact, there is a strong correlation between the level of **GSH** and DNA-adducts.^34^ In the benzo[*a*]pyrene treatment, two isomers of benzo[*a*]pyrene diol epoxide connected to **cysgly** were detected (library item *Benzo[a]pyrene cysteinylglycine A-B*). Our findings suggest that the **MAP** successfully converts potential mutagenic species into a detoxified metabolite. However, the exact configuration of the diol epoxide remains unknown.

**Figure 4:**
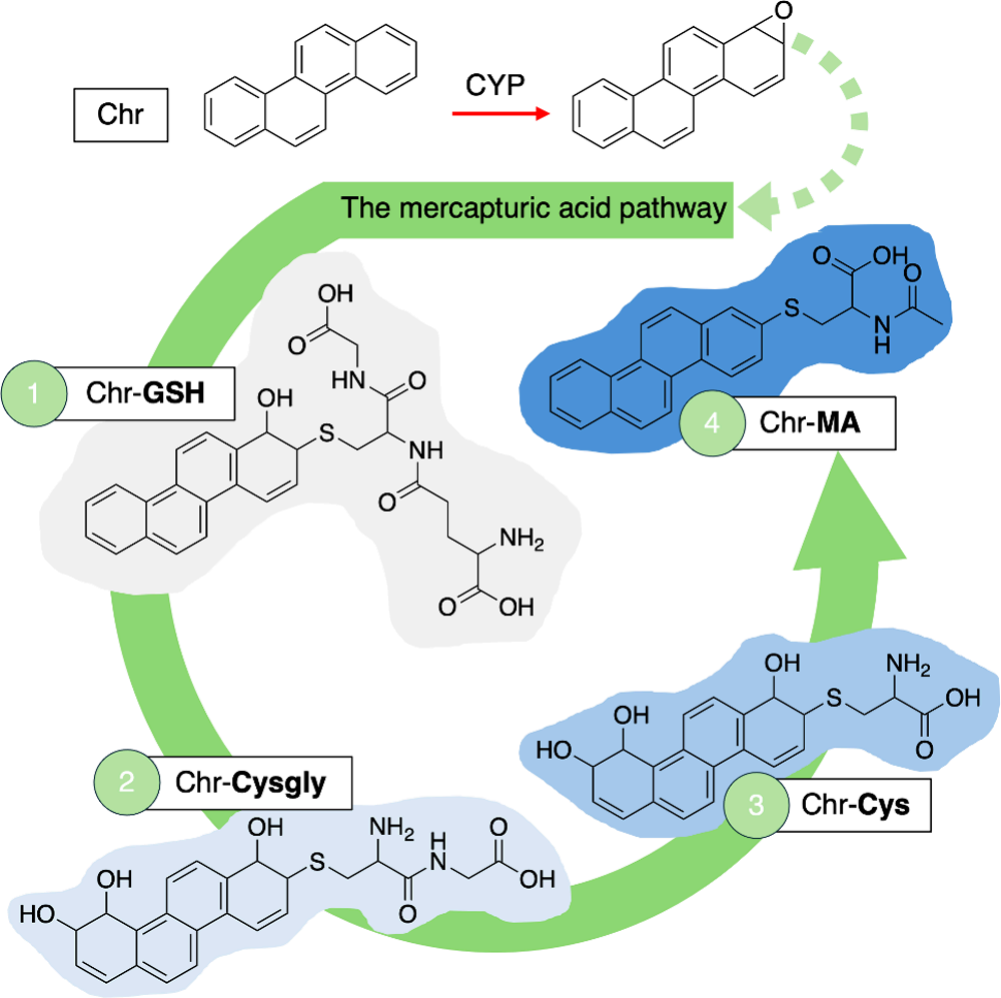
Chrysene (Chr) can be bioactivated by cytochrome P450 (CYP) enzymes to an epoxide and detoxified by the mercapturic acid pathway (**MAP**), producing metabolites with (1) Glutathione (**GSH**), (2) Cysteinylglycine (**cysgly**), (3) Cysteine (**cys**), and (4) Mercapturic acid (**MA**) conjugates.

Chrysene yielded metabolites of all four conjugation types, which allows for mapping of the entire pathway, as depicted in Figure 4. The metabolic route can be initiated by the cytochrome P450 enzymes which can form a highly reactive epoxide metabolite.^12^ By conjugation with **GSH**, the epoxides are detoxified (1) and can continue the pathway as a **cysgly** conjugate (2) in the **MAP**. In the case outlined in Figure 4, there is an additional dihydrodiol moiety present compared with the previous **GSH** step (1). Our hypothesis suggests that Phase I oxidations may occur both prior to and following the conjugation events. Moving forward to a **cys** metabolite, the same Phase I moieties were observed (3). Further metabolic processes can give rise to **MA** (4). However, no indication of other functional groups was observed in the mass spectra of this compound. A proposed explanation is the elimination of water, leaving the **GSH** residue singly on the ring.^35,36^ This observation, where only the **MAP** conjugate was observed on the PAH, occurred in 13 of the 33 compounds identified in our library.

The elimination of PAHs via bile is affected by environmental factors and dynamic physiological processes.^37^ It should be emphasized that the metabolism in **MAP** is a continuous process, and our sampling only captured a snapshot of the metabolic products. In principle, it is not anticipated that all these metabolites will persist simultaneously in the bile for an extended duration, as observed in the Chrysene treatment (Figure 4). The **MAP** is an interorgan process where the formation of the **MA** conjugate usually occurs in the kidney.^15^ These S-conjugates can also re-enter the liver-bile detoxification system and prolong their presence by enterohepatic circulation.^13^ Therefore, the detections presented in Figure 4 of Chrysene metabolites are unique, as all versions in the **MAP** are available in one bile extract. These conjugates exhibit high polarity, making them readily excretable in urine and less likely to exist in bile.

### Analytical Workflow

The employed screening strategy integrated both a specific CNL and a diagnostic fragment ion for each conjugate, which allowed for selective data filtering. The characteristic fragmentation behaviors of the four conjugation types (Figure 1) are reported in Table 1. The CNL enables the detection of the characteristic PAH metabolite (Phase I; with or without sulfur retained), while a fragment ion confirms the identity of the conjugate.

The candidates matching the characteristics in Table 1 were further prioritized by incorporating predicted CCS values for extra identification confidence.^38^ The presented strategy accomplishes the detection of **MAP** metabolites in one injection of the sample using distinct combinations of compound characteristics. Based on the observed fragmentation patterns of the conjugates, we expect that the presented workflow also can be employed in xenobiotic scenarios beyond PAHs.

The conventional strategy for the detection of **GSH** and **MA** metabolites is a CNL of 129 Da.^39,40^ Another approach is negative precursor ion scanning of *m*/*z* 272 targeting **GSH**s.^41−43^ In contrast to the conventional methods that use CNL or precursor scans followed by product ion scans for structural elucidation work, additional injections were not necessary with MS^E^. Efforts to find well-documented analytical methods for **cysgly** and **cys** beyond the thorough elucidation presented by Levsen et al.^36^ were unsuccessful. We hypothesize that this diminished emphasis is due to the expected efficiency of the **MAP,** where the significance of intermediates is overshadowed by the initial **GSH** and the ultimate conjugate **MA**.

Two specific analytical challenges arise when analyzing the metabolites presented herein. First, it concerns the similarity in mass of conjugates: conjugation with **cysgly** (+C_5_H_8_N_2_O_3_S) and O-glucuronidation (+C_6_H_8_O_6_) adds 176.02556 and 176.03209 Da, respectively. Both have the same CNL strategy of monitoring 176 Da losses.^36^ Thorough investigation of spectra is therefore crucial to make the correct annotation based on fragment ions. Second, two key fragments of the metabolites have striking similarity in mass. Coincidentally, the mass of a biotransformed PAH into either a PAH-diol (containing two −OH groups) or a PAH-thiol (−SH group) differs by only 0.018 Da, where the difference appears in the second decimal. Hence, it is unlikely to make the same discovery with low resolution mass spectrometry since the technique lacks the ability to distinguish two peaks of slightly different mass-to-charge ratios. Benz[a]anthracene **cysgly** was the first metabolite to be elucidated after recognizing this small, but crucial mass difference. PAH-thiols were implemented in the workflow by reprocessing the data (*see Data Processing and Library Building)*. This facilitated real-time investigations, eliminating the need for sample reinjection, and thereby conserving both time and chemicals.

The limited identifications of **MA** and **cys** (Figure 3) could be attributed to biological factors as discussed earlier or the specificity of the method. For instance, **cys** did not have diagnostic fragments beyond *m*/*z* 141, corresponding to the inclusion of carbon bonds from PAH. Ideally, we would have observed a fragment corresponding to the entire conjugate, but this was not present. However, not all conjugates exhibit the same characteristic loss during fragmentation^44^ or produce fragments at detectable levels. All of the involved conjugates are S-conjugates, also known as thioether conjugates. We observe variations in fragmentation pattern despite identical conjugation type, suggesting a strong influence of the aromatic system and the number of Phase I modifications on the PAH. Ma et al.^45^ reported 98% specificity for 121 CNL for **cys**. In our case, sulfur is retained, resulting in losses of 87 and 139 Da (Table 1).

### Application of Library to Oil-Polluted Fish

Environmental samples typically contain PAHs from various sources. Hence, two relevant pollution sources were considered in this study: crude oil and PW. Increased expression of GST has been observed following oil exposure studies in early life stages of haddock.^46^ Thus, we anticipated the presence of **MAP** metabolites in marine fish exposed to crude oil mixtures. The detections corroborated the hypothesis as **GSH** and **cysgly** candidates matching alkylated 3-ring PAHs and a 4-ring PAH were identified (Table 2). This demonstrates that the formation of such metabolites is not limited to single compound exposures but also occurs in realistic and highly complex mixtures such as crude oil.

**Table 2:** Tentatively Identified Polycyclic Aromatic Hydrocarbon (PAH) Metabolites from the Mercapturic Acid Pathway (**MAP**) produced by Atlantic haddock after Exposure to Crude Oil

PAH type	Conjugate	Formula	Observed mass (*m*/*z*)	Observed common neutral loss (Da)	Observed diagnostic fragment(s) (*m/z*)
*Methylated 3-ring PAH*	**Cysgly**	C_20_H_20_N_2_O_3_S	367.1117	144.0532	143.0462
	**GSH**	C_25_H_27_N_3_O_6_S	496.1547	273.0960	254.0784, 272.0887
*Dimethylated 3-ring PAH*	**Cysgly**	C_21_H_22_N_2_O_3_S	381.1277	144.0533	143.0463
	**GSH**	C_26_H_29_N_3_O_6_S	510.1708	273.0961	254.0784, 272.0889
		C_26_H_29_N_3_O_6_S	510.1703	273.0960	272.0889
*4-ring PAH*	**Cysgly**	C_23_H_20_N_2_O_3_S	403.1124	144.0535	143.0465

Petrogenic PAHs dominate in crude oil polluted samples, characterized by a predominant presence of alkylated low molecular PAHs (2- and 3-ring) and modest contributions from heavier unsubstituted PAHs.^47^ Based on their large contributions in crude oil, the 3-ring PAHs are expected to be phenanthrene derivatives and the 4-ring is suspected to be pyrene or chrysene.^26^ Detection information (*m*/*z*, retention time, CCS) for all of the metabolites is reported in Table S2.

The formulas presented are the best fitted formula according to obtained *m*/*z* and isotope pattern using the elemental composition calculator in UNIFI. As observed in our metabolite library, the **MAP** metabolites exhibit a wide range of retention times, highlighting the significance of the substitution position in terms of polarity. Given the resemblance in detection information among isomers, identifying the compounds becomes impractical. We therefore assign generic names to the compound due to the numerous potential options available.

The second oil pollution treatment, PW, represents operational discharge containing the most water-soluble, low molecular weight PAHs.^26^ Given these considerations, we suspected a predominant presence of naphthalene metabolites in fish exposed to PW. Yet, no metabolites from C0-, C1-, or C2-naphthalene were elucidated in bile from either single compound, crude oil, or PW exposures. In fact, no **MAP** metabolites were detected by our workflow. Effective biotransformation processes can result in low internal concentration in fish despite high contribution in the polluted water,^48^ so we hypothesize that rapid detoxification following the exposure could be a contributing factor. We also acknowledge the possibility of loss of compounds during extraction (*e.g*. volatilization) or due to instrument limitations (i.e., inadequate level of detection). It is worth mentioning that it initially appeared to be a match with 1-methylphenanthrene, but upon closer examination of the base peak and isotopic pattern, the candidate was dismissed. Based on the detection of characteristic fragments *m*/*z* 272, 179, and 128, we are confident that the compound contains **GSH**. However, the exact source linkage from which parent compound it originated remains ambiguous.

### Diversification of Metabolite Types Can Improve Environmental Monitoring

Enzymatic deconjugation to reconstruct the original Phase I metabolites is typically performed for quantitative investigations of PAH exposure.^10^ It is noteworthy that metabolites from the **MAP** could be present in samples using conventional extraction methods: the deconjugation enzymes (i.e., β-glucuronidase and aryl sulfatase) that recovers Phase I metabolites, do not hydrolyze these conjugates.^10,25^ As a result, they may potentially go unnoticed in the mixture. The inclusion of **GSH** metabolites can be accomplished by an extra enzymatic deconjugation step by addition of gamma-glutamyltranspeptidase^49^ or by performing acidic hydrolysis.^50^ This extra effort during sample preparation would give a more accurate overview of metabolite quantities and ultimately provide more thorough assessments of PAH exposure. An augmentation like this could enhance detection sensitivity at lower concentrations, thereby enabling the adoption of more refined analytical techniques.

The present study is a qualitative and nontargeted assessment of metabolites, identifying new **MAP** compounds previously unknown in fish but recognized in mammalian research.^14^ Ayala et al.^16^ quantitatively measured the urinary excretion of naphthalene metabolites and found that **GSH** and **MA** accounted for >60% of the total measured metabolites in mice. **MA**s have also been identified as urinary metabolites in humans.^51^ Contrary to the findings of Ayala et al.,^16^**MA**s have been characterized as minor metabolites in cigarette smokers.^35^ However, that study measured only one out of the four potential conjugates in the **MAP**. Similarly, Willet et al.^49^ observed a minimal presence of **GSH** metabolites in fish bile after exposure to benzo[a]pyrene. The present results imply that these limited detections might be due to the sequential metabolization of **GSH** into **cysgly**, **cys**, or **MA** derivates which are subsequently eliminated through Phase III pathways.^14^ Thus, the current identifications warrant further investigation into the enzymatic pathways initiated by GST.

This work demonstrates the discovery of novel metabolites by utilizing the power of IM-HRMS and the possibility to perform retrospective analysis. The controlled exposures of individual components in our study allowed us to develop a comprehensive metabolite library using our own sample set. This innovative approach proved instrumental in identifying previously undiscovered metabolites in fish exposed to crude oil. Specifically, our method enhances the scope of PAH metabolite analyses, offering a new approach for detecting and characterizing a wider range of metabolites compared to traditional methodologies.

The objective of the presented bile extracts was to qualitatively profile PAH metabolites to gain a deeper understanding of the metabolic pathways involved in their detoxification. Due to minimal reporting on metabolites of the **MAP** in the literature, these were not prioritized but discovered incidentally. Hence, it is likely that these xenobiotic metabolites frequently escape detection, leading to underestimated exposure assessments. In essence, this metabolite class should be incorporated into future investigations to enhance our understanding of the impact of chronic emissions of contaminants. By employing the methods outlined in this work, high confidence tentative identifications can be achieved.

## Supporting Information Available

Metabolite library (PDF, ZIP)
Detection results of identified metabolites and detection results from crude oil exposure (PDF)
