# Supplementary material for: Exposure of Polycyclic Aromatic Hydrocarbons (PAHs) and Crude Oil to Atlantic Haddock (Melanogrammus aeglefinus): A Unique Snapshot of the Mercapturic Acid Pathway
Source: Environ Sci Technol. 2024 Aug 5;58(33):14855–63. doi: 10.1021/acs.est.4c05112 (PMC11340023; doi:10.1021/acs.est.4c05112)
Supplement: Supplementary file 1 — es4c05112_si_001.pdf [file es4c05112_si_001.pdf]

Metabolite library

- 1,4-Dimethylphenanthrene cysteinylglycine A
- 1,4-Dimethylphenanthrene cysteinylglycine B
- 1,4-Dimethylphenanthrene glutathione I
- 1,4-Dimethylphenanthrene glutathione II
- 1-Methylphenanthrene cysteinylglycine A
- 1-Methylphenanthrene cysteinylglycine B
- 1-Methylphenanthrene glutathione I
- 1-Methylphenanthrene glutathione II
- Anthracene cysteinylglycine
- Benz(a)anthracene cysteinylglycine I A
- Benz(a)anthracene cysteinylglycine I B
- Benz(a)anthracene cysteinylglycine II
- Benz(a)anthracene cysteinylglycine III
- Benz(a)anthracene cysteinylglycine IV A
- Benz(a)anthracene cysteinylglycine IV B
- Benz(a)anthracene glutathione I A
- Benz(a)anthracene glutathione I B
- Benz(a)anthracene glutathione II
- Benzo(a)pyrene cysteinylglycine A
- Benzo(a)pyrene cysteinylglycine B
- Chrysene cysteine
- Chrysene cysteinylglycine I A
- Chrysene cysteinylglycine I B
- Chrysene cysteinylglycine II
- Chrysene glutathione I A
- Chrysene glutathione I B
- Chrysene glutathione II
- Chrysene mercapturic acid
- Dibenz(a,h)anthracene glutathione I A
- Dibenz(a,h)anthracene glutathione I B
- Dibenz(a,h)anthracene glutathione II
- Phenanthrene cysteinylglycine
- Phenanthrene glutathione
- Mol files
  - 1,4-Dimethylphenanthrene cysteinylglycine A
  - 1,4-Dimethylphenanthrene cysteinylglycine B
  - 1,4-Dimethylphenanthrene glutathione I
  - 1,4-Dimethylphenanthrene glutathione II
  - 1-Methylphenanthrene cysteinylglycine A
  - 1-Methylphenanthrene cysteinylglycine B
  - 1-Methylphenanthrene glutathione I
  - 1-Methylphenanthrene glutathione II
  - Anthracene cysteinylglycine
  - Benz(a)anthracene cysteinylglycine I A
  - Benz(a)anthracene cysteinylglycine I B
  - Benz(a)anthracene cysteinylglycine II
  - Benz(a)anthracene cysteinylglycine III
  - Benz(a)anthracene cysteinylglycine IV A
  - Benz(a)anthracene cysteinylglycine IV B
  - Benz(a)anthracene glutathione I A

Benz(a)anthracene glutathione I B
Benz(a)anthracene glutathione II
Benzo(a)pyrene cysteinylglycine A
Benzo(a)pyrene cysteinylglycine B
Chrysene cysteine
Chrysene cysteinylglycine I A
Chrysene cysteinylglycine I B
Chrysene cysteinylglycine II
Chrysene glutathione I A
Chrysene glutathione I B
Chrysene glutathione II
Chrysene mercapturic acid
Dibenz(a,h)anthracene glutathione I A
Dibenz(a,h)anthracene glutathione I B
Dibenz(a,h)anthracene glutathione II
Phenanthrene cysteinylglycine
Phenanthrene glutathione

1,4-Dimethylphenanthrene cysteinylglycine A

Compound data	Proposed structure
**Conjugate**: cysteinylglycine<br>**Treatment**: 1,4-dimethylphenanthrene<br>**Formula**: $C_{21}H_{22}N_2O_3S$<br>**Neutral mass (Da)**: 382.1351<br>**Adduct**: -H<br>**Expected mass (Da)**: 381.1278<br>**Observed mass (Da)**: 381.1284<br>**Predicted CCS (Å^2)**: 197.24<br>**Observed CCS (Å^2)**: 200.21<br>**Δ CCS (%)**: 1.5<br>**Observed drift time (ms)**: 6.73<br>**Observed retention time (min)**: 5.5	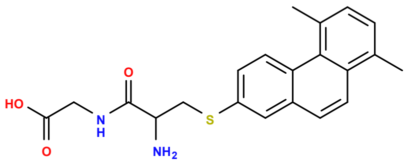

`SMILES: OC(=O)CNC(=O)C(CSc1ccc2c(c1)ccc1c2c(C)ccc1C)N` mol

Mass spectra:

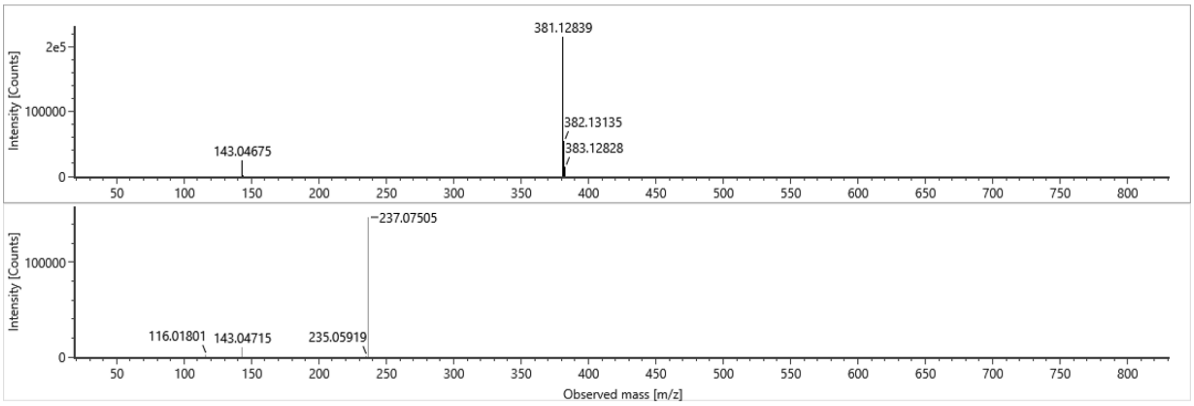

Low energy (top) and high energy (bottom) mass spectra

1,4-Dimethylphenanthrene cysteinylglycine B

Compound data	Proposed structure
**Conjugate**: cysteinylglycine<br>**Treatment**: 1,4-dimethylphenanthrene<br>**Formula**: $C_{21}H_{22}N_2O_3S$<br>**Neutral mass (Da)**: 382.1351<br>**Adduct**: -H<br>**Expected mass (Da)**: 381.1278<br>**Observed mass (Da)**: 381.1281<br>**Predicted CCS (Å^2)**: 192.67<br>**Observed CCS (Å^2)**: 194.36<br>**Δ CCS (%)**: 0.9<br>**Observed drift time (ms)**: 6.50<br>**Observed retention time (min)**: 7.5	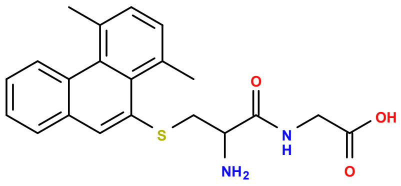

`SMILES: OC(=O)CNC(=O)C(CSc1cc2ccccc2c2c1c(C)ccc2C)N` mol

Mass spectra:

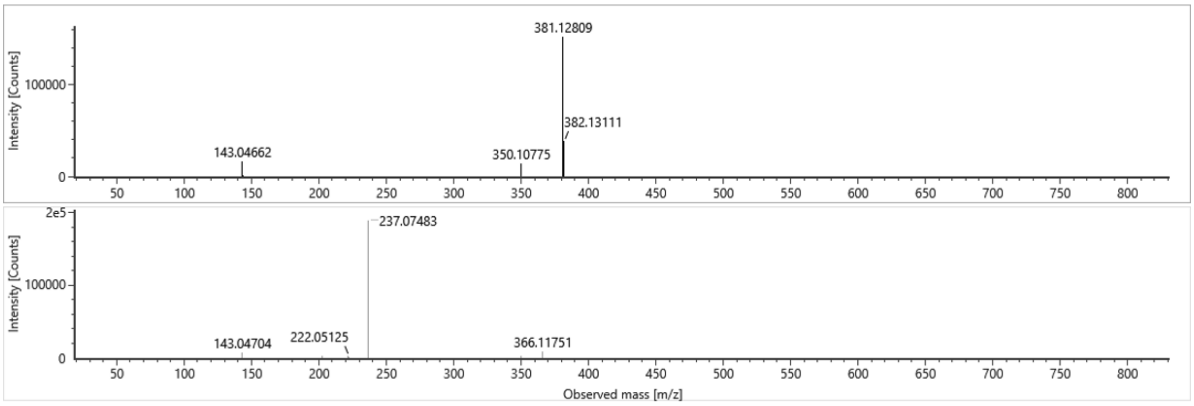

Low energy (top) and high energy (bottom) mass spectra

1,4-Dimethylphenanthrene glutathione I

Compound data	Proposed structure
**Conjugate**: glutathione<br>**Treatment**: 1,4-dimethylphenanthrene<br>**Formula**: $C_{26}H_{29}N_3O_6S$<br>**Neutral mass (Da)**: 511.1777<br>**Adduct**: -H<br>**Expected mass (Da)**: 510.1704<br>**Observed mass (Da)**: 510.1708<br>**Predicted CCS (Å^2)**: 222.69<br>**Observed CCS (Å^2)**: 217.24<br>**Δ CCS (%)**: -2.4<br>**Observed drift time (ms)**: 7.42<br>**Observed retention time (min)**: 7.6	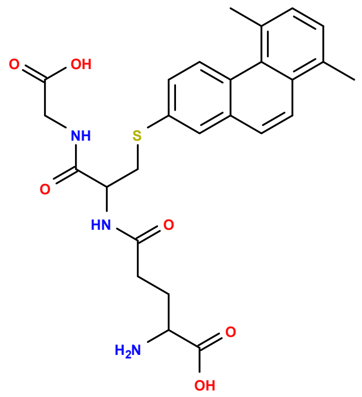

`SMILES: O=C(NC(C(=O)NCC(=O)O)CSc1ccc2c(c1)ccc1c2c(C)ccc1C)CCC(C(=O)O)N` mol

Mass spectra:

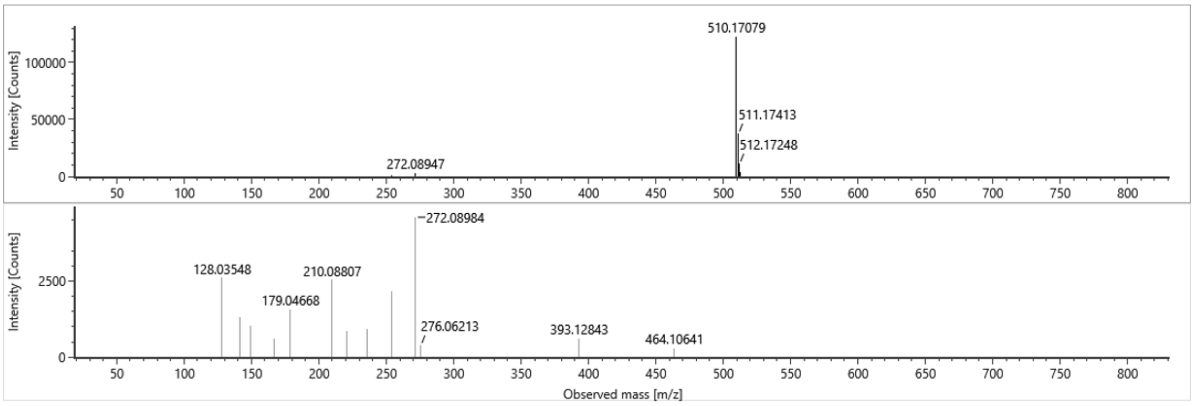

Low energy (top) and high energy (bottom) mass spectra

1,4-Dimethylphenanthrene glutathione II

Compound data	Proposed structure
**Conjugate**: glutathione<br>**Treatment**: 1,4-dimethylphenanthrene<br>**Formula**: $C_{26}H_{31}N_3O_7S$<br>**Neutral mass (Da)**: 529.1883<br>**Adduct**: -H<br>**Expected mass (Da)**: 528.1810<br>**Observed mass (Da)**: 528.1816<br>**Predicted CCS (Å^2)**: 226.54<br>**Observed CCS (Å^2)**: 219.22<br>**Δ CCS (%)**: -3.2<br>**Observed drift time (ms)**: 7.51<br>**Observed retention time (min)**: 5.3	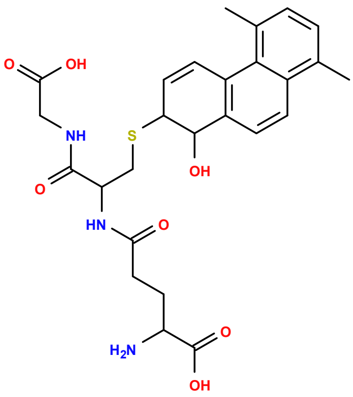

`SMILES: O=C(NC(C(=O)NCC(=O)O)CSC1C=Cc2c(C1O)ccc1c2c(C)ccc1C)CCC(C(=O)O)N` mol

Mass spectra:

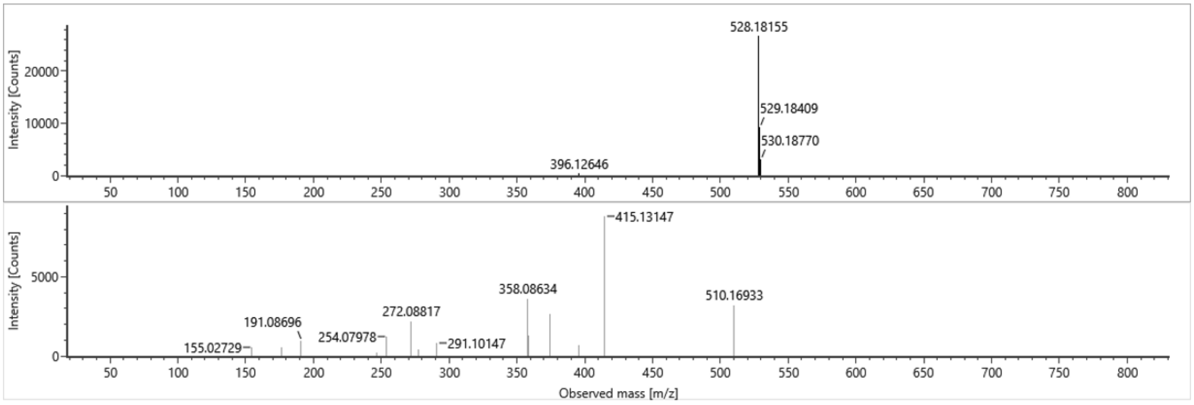

Low energy (top) and high energy (bottom) mass spectra

1-Methylphenanthrene cysteinylglycine A

Compound data	Proposed structure
**Conjugate**: cysteinylglycine<br>**Treatment**: 1-methylphenanthrene<br>**Formula**: $C_{20}H_{20}N_2O_3S$<br>**Neutral mass (Da)**: 368.1195<br>**Adduct**: -H<br>**Expected mass (Da)**: 367.1122<br>**Observed mass (Da)**: 367.1121<br>**Predicted CCS (Å^2)**: 189.01<br>**Observed CCS (Å^2)**: 191.70<br>**Δ CCS (%)**: 1.4<br>**Observed drift time (ms)**: 6.39<br>**Observed retention time (min)**: 5.0	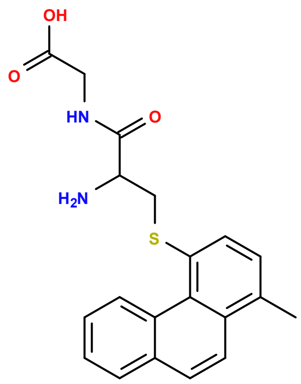

`SMILES: OC(=O)CNC(=O)C(CSc1ccc(c2c1c1ccccc1cc2)C)N` mol

Mass spectra:

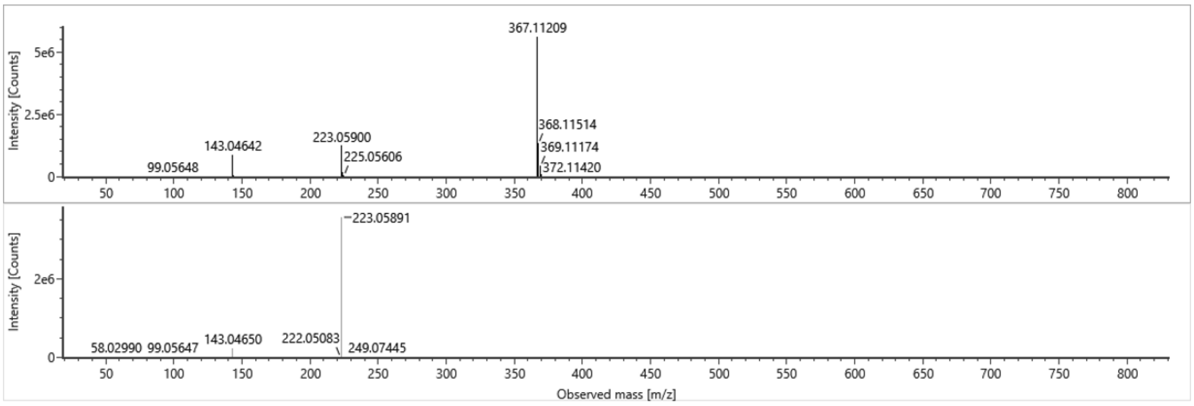

Low energy (top) and high energy (bottom) mass spectra

1-Methylphenanthrene cysteinylglycine B

Compound data	Proposed structure
**Conjugate**: cysteinylglycine<br>**Treatment**: 1-methylphenanthrene<br>**Formula**: $C_{20}H_{20}N_2O_3S$<br>**Neutral mass (Da)**: 368.1195<br>**Adduct**: -H<br>**Expected mass (Da)**: 367.1122<br>**Observed mass (Da)**: 367.1127<br>**Predicted CCS (Å^2)**: 188.80<br>**Observed CCS (Å^2)**: 192.08<br>**Δ CCS (%)**: 1.7<br>**Observed drift time (ms)**: 6.40<br>**Observed retention time (min)**: 8.5	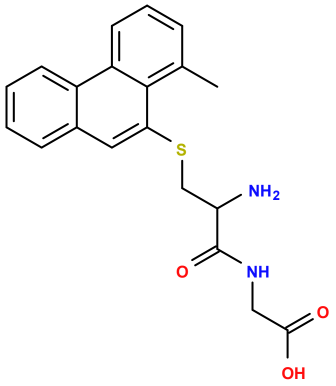

`SMILES: OC(=O)CNC(=O)C(CSc1cc2ccccc2c2c1c(C)ccc2)N` mol

Mass spectra:

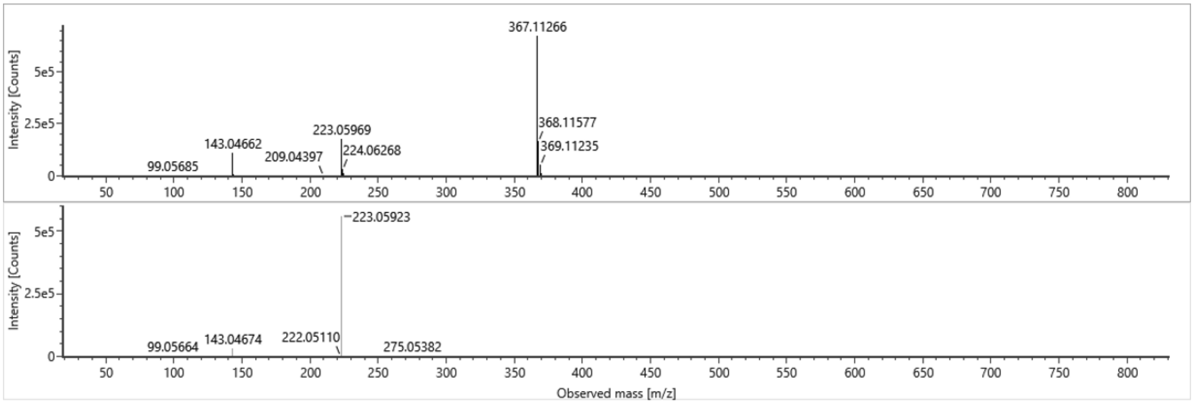

Low energy (top) and high energy (bottom) mass spectra

1-Methylphenanthrene glutathione I

Compound data	Proposed structure
**Conjugate**: glutathione<br>**Treatment**: 1-methylphenanthrene<br>**Formula**: $C_{25}H_{27}N_3O_6S$<br>**Neutral mass (Da)**: 497.1621<br>**Adduct**: -H<br>**Expected mass (Da)**: 496.1548<br>**Observed mass (Da)**: 496.1547<br>**Predicted CCS (Å^2)**: 217.56<br>**Observed CCS (Å^2)**: 213.51<br>**Δ CCS (%)**: -1.9<br>**Observed drift time (ms)**: 7.27<br>**Observed retention time (min)**: 5.8	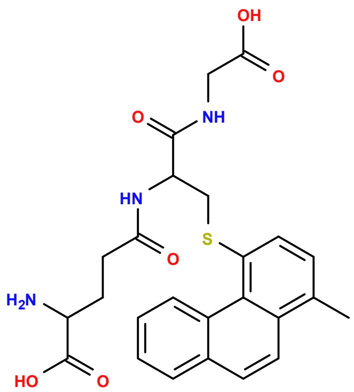

`SMILES: O=C(NC(C(=O)NCC(=O)O)CSc1ccc(c2c1c1ccccc1cc2)C)CCC(C(=O)O)N` mol

Mass spectra:

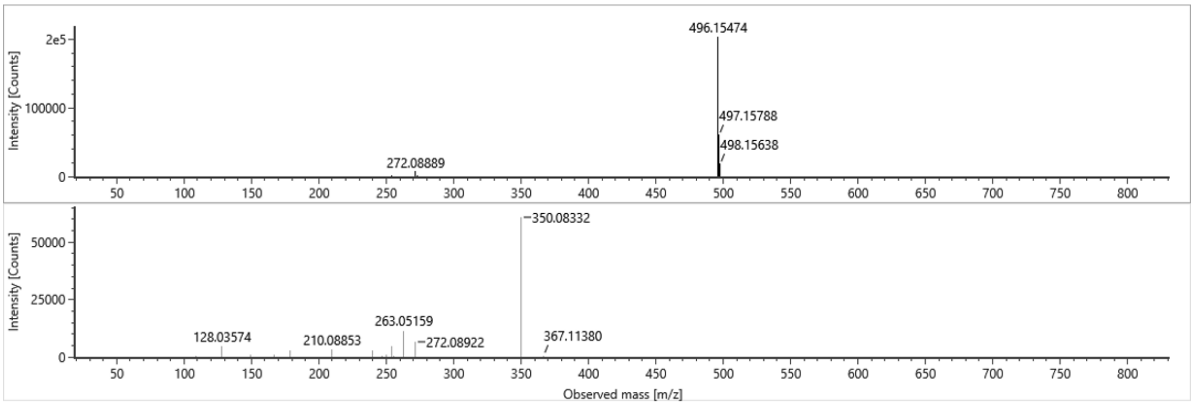

Low energy (top) and high energy (bottom) mass spectra

1-Methylphenanthrene glutathione II

Compound data	Proposed structure
**Conjugate**: glutathione<br>**Treatment**: 1-methylphenanthrene<br>**Formula**: $C_{25}H_{29}N_3O_7S$<br>**Neutral mass (Da)**: 515.1726<br>**Adduct**: -H<br>**Expected mass (Da)**: 514.1653<br>**Observed mass (Da)**: 514.1657<br>**Predicted CCS (Å^2)**: 221.32<br>**Observed CCS (Å^2)**: 218.20<br>**Δ CCS (%)**: -1.4<br>**Observed drift time (ms)**: 7.46<br>**Observed retention time (min)**: 2.2	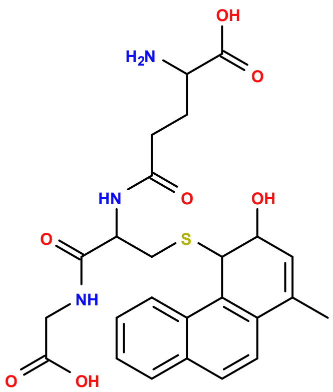

SMILES: O=C(NC(C(=O)NCC(=O)O)CSC1C(O)C=C(c2c1c1ccccc1cc2)C)CCC(C(=O)O)N mol

Mass spectra:

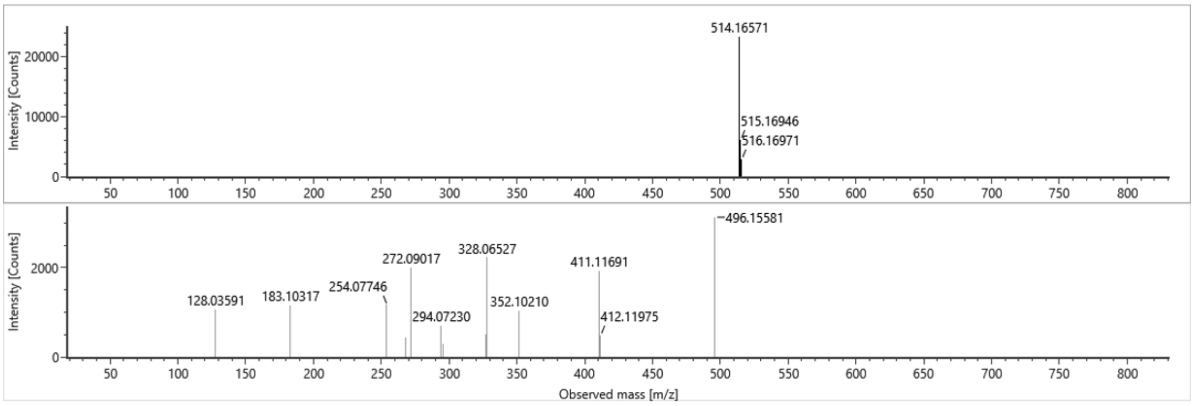

Low energy (top) and high energy (bottom) mass spectra

Anthracene cysteinylglycine

Compound data	Proposed structure
**Conjugate**: cysteinylglycine<br>**Treatment**: anthracene<br>**Formula**: $C_{19}H_{18}N_2O_3S$<br>**Neutral mass (Da)**: 354.1038<br>**Adduct**: -H<br>**Expected mass (Da)**: 353.0965<br>**Observed mass (Da)**: 353.0972<br>**Predicted CCS (Å^2)**: 185.41<br>**Observed CCS (Å^2)**: 189.08<br>**Δ CCS (%)**: 2.0<br>**Observed drift time (ms)**: 6.27<br>**Observed retention time (min)**: 2.5	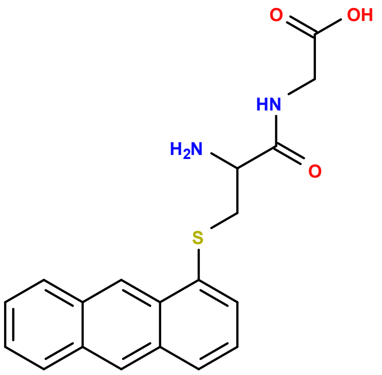

`SMILES: OC(=O)CNC(=O)C(CSc1cccc2c1cc1ccccc1c2)N` mol

Mass spectra:

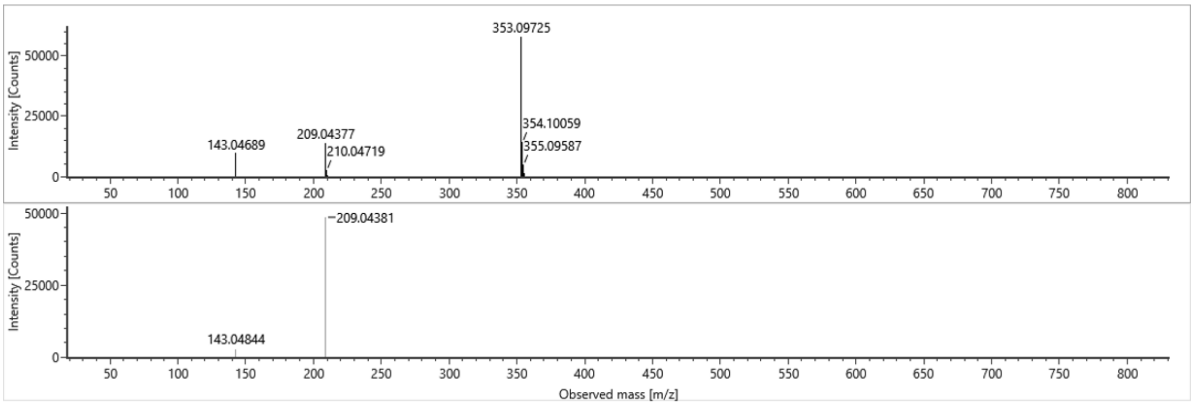

Low energy (top) and high energy (bottom) mass spectra

Benz(a)anthracene cysteinylglycine I A

Compound data	Proposed structure
**Conjugate**: cysteinylglycine<br>**Treatment**: benz(a)anthracene<br>**Formula**: $C_{23}H_{20}N_2O_3S$<br>**Neutral mass (Da)**: 404.1195<br>**Adduct**: -H<br>**Expected mass (Da)**: 403.1122<br>**Observed mass (Da)**: 403.1123<br>**Predicted CCS (Å^2)**: 200.23<br>**Observed CCS (Å^2)**: 203.38<br>**Δ CCS (%)**: 1.6<br>**Observed drift time (ms)**: 6.86<br>**Observed retention time (min)**: 6.2	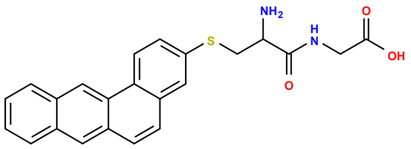

`SMILES: OC(=O)CNC(=O)C(CSc1ccc2c(c1)ccc1c2cc2ccccc2c1)N` mol

Mass spectra:

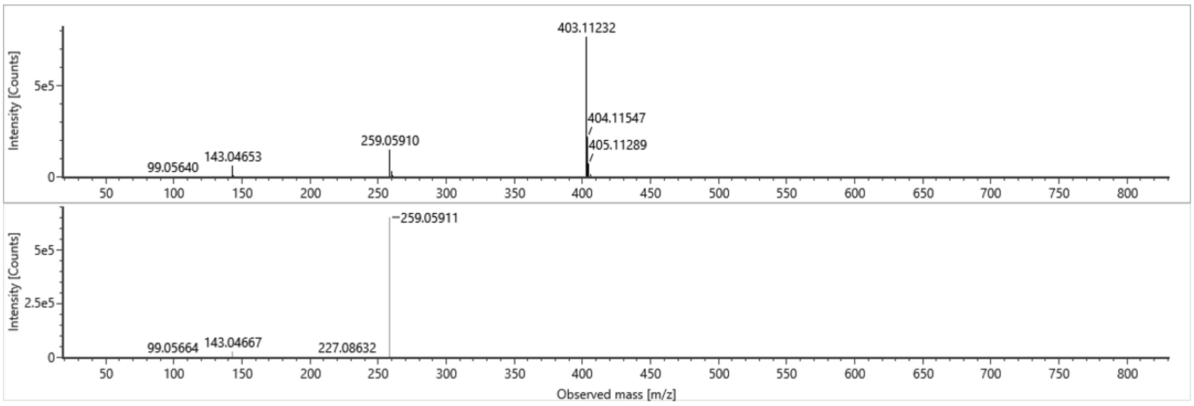

Low energy (top) and high energy (bottom) mass spectra

Benz(a)anthracene cysteinylglycine I B

Compound data	Proposed structure
**Conjugate**: cysteinylglycine<br>**Treatment**: benz(a)anthracene<br>**Formula**: $C_{23}H_{20}N_2O_3S$<br>**Neutral mass (Da)**: 404.1195<br>**Adduct**: -H<br>**Expected mass (Da)**: 403.1122<br>**Observed mass (Da)**: 403.1122<br>**Predicted CCS (Å^2)**: 200.21<br>**Observed CCS (Å^2)**: 204.83<br>**Δ CCS (%)**: 2.3<br>**Observed drift time (ms)**: 6.92<br>**Observed retention time (min)**: 5.9	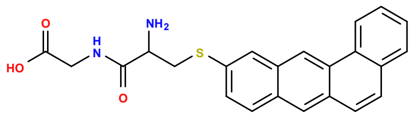

`SMILES: OC(=O)CNC(=O)C(CSc1ccc2c(c1)cc1c(c2)ccc2c1cccc2)N` mol

Mass spectra:

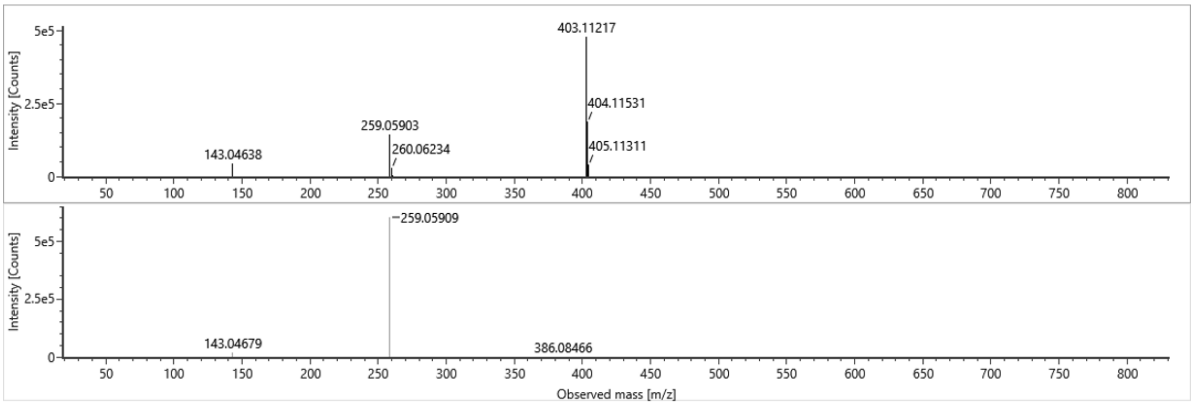

Low energy (top) and high energy (bottom) mass spectra

Benz(a)anthracene cysteinylglycine II

Compound data	Proposed structure
**Conjugate**: cysteinylglycine<br>**Treatment**: benz(a)anthracene<br>**Formula**: $C_{23}H_{22}N_2O_4S$<br>**Neutral mass (Da)**: 422.1300<br>**Adduct**: -H<br>**Expected mass (Da)**: 421.1228<br>**Observed mass (Da)**: 421.1230<br>**Predicted CCS (Å^2)**: 202.97<br>**Observed CCS (Å^2)**: 203.92<br>**Δ CCS (%)**: 0.5<br>**Observed drift time (ms)**: 6.89<br>**Observed retention time (min)**: 4.4	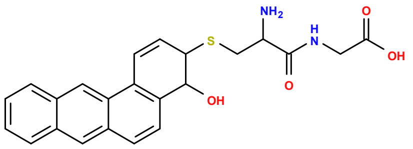

`SMILES: OC(=O)CNC(=O)C(CSC1C=Cc2c(C1O)ccc1c2cc2ccccc2c1)N` mol

Mass spectra:

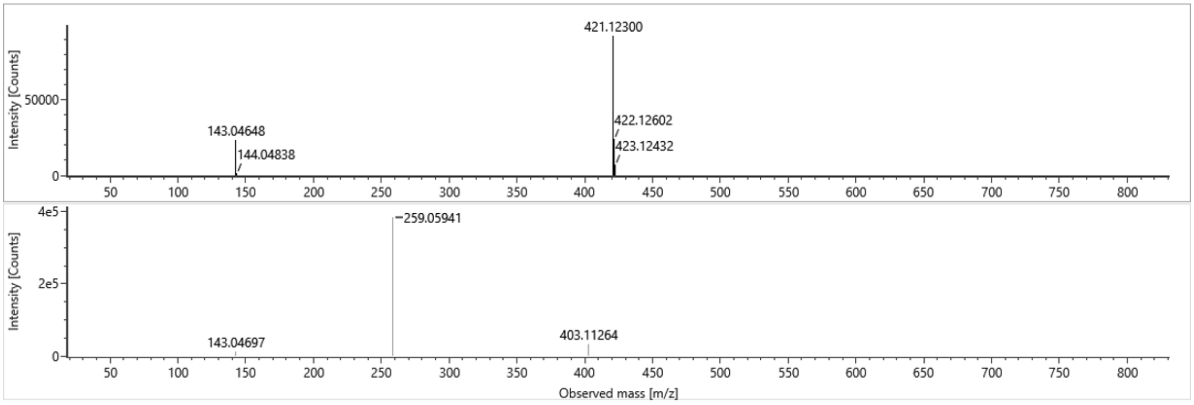

Low energy (top) and high energy (bottom) mass spectra

Benz(a)anthracene cysteinylglycine III

Compound data	Proposed structure
**Conjugate**: cysteinylglycine<br>**Treatment**: benz(a)anthracene<br>**Formula**: $C_{23}H_{22}N_2O_5S$<br>**Neutral mass (Da)**: 438.1249<br>**Adduct**: -H<br>**Expected mass (Da)**: 437.1177<br>**Observed mass (Da)**: 437.1181<br>**Predicted CCS (Å^2)**: 205.70<br>**Observed CCS (Å^2)**: 196.72<br>**Δ CCS (%)**: -4.4<br>**Observed drift time (ms)**: 6.60<br>**Observed retention time (min)**: 2.2	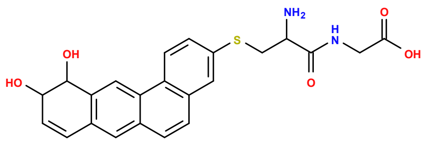

`SMILES: OC(=O)CNC(=O)C(CSc1ccc2c(c1)ccc1c2cc2c(c1)C=CC(C2O)O)N` mol

Mass spectra:

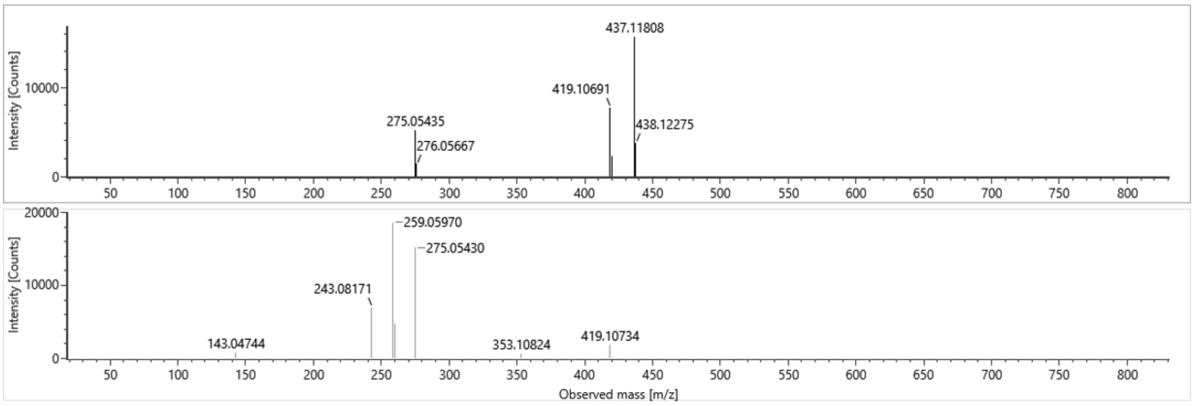

Low energy (top) and high energy (bottom) mass spectra

Benz(a)anthracene cysteinylglycine IV A

Compound data	Proposed structure
**Conjugate**: cysteinylglycine<br>**Treatment**: benz(a)anthracene<br>**Formula**: $C_{23}H_{24}N_2O_6S$<br>**Neutral mass (Da)**: 456.1355<br>**Adduct**: -H<br>**Expected mass (Da)**: 455.1282<br>**Observed mass (Da)**: 455.1289<br>**Predicted CCS (Å^2)**: 208.46<br>**Observed CCS (Å^2)**: 208.64<br>**Δ CCS (%)**: 0.1<br>**Observed drift time (ms)**: 7.08<br>**Observed retention time (min)**: 2.8	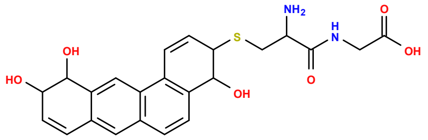

`SMILES: OC(=O)CNC(=O)C(CSC1C=Cc2c(C1O)ccc1c2cc2c(c1)C=CC(C2O)O)N` mol

Mass spectra:

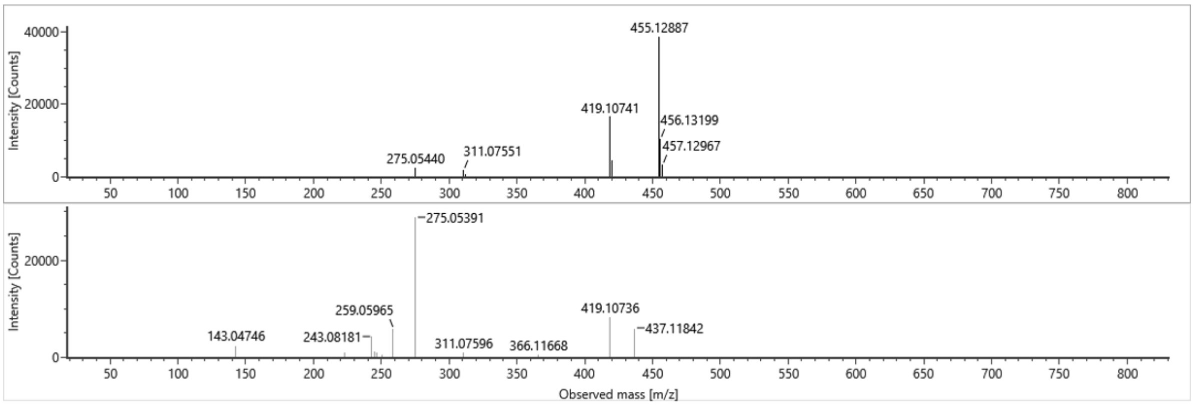

Low energy (top) and high energy (bottom) mass spectra

Benz(a)anthracene cysteinylglycine IV B

Compound data	Proposed structure
**Conjugate**: cysteinylglycine<br>**Treatment**: benz(a)anthracene<br>**Formula**: $C_{23}H_{24}N_2O_6S$<br>**Neutral mass (Da)**: 456.1355<br>**Adduct**: -H<br>**Expected mass (Da)**: 455.1282<br>**Observed mass (Da)**: 455.1283<br>**Predicted CCS (Å^2)**: 208.43<br>**Observed CCS (Å^2)**: 209.13<br>**Δ CCS (%)**: 0.3<br>**Observed drift time (ms)**: 7.10<br>**Observed retention time (min)**: 3.7	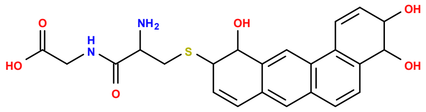

`SMILES: OC(=O)CNC(=O)C(CSC1C=Cc2c(C1O)cc1c(c2)ccc2c1C=CC(C2O)O)N` mol

Mass spectra:

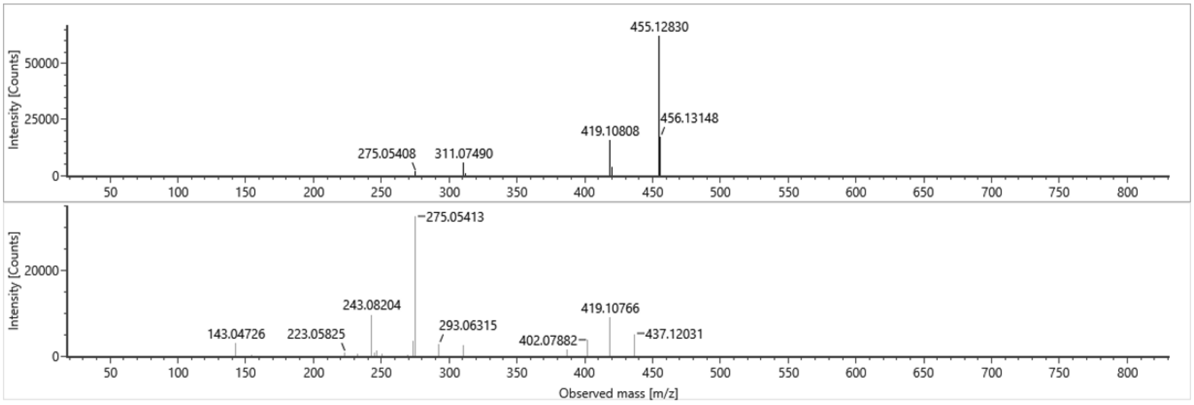

Low energy (top) and high energy (bottom) mass spectra

Benz(a)anthracene glutathione I A

Compound data	Proposed structure
**Conjugate**: glutathione<br>**Treatment**: benz(a)anthracene<br>**Formula**: $C_{28}H_{29}N_3O_7S$<br>**Neutral mass (Da)**: 551.1726<br>**Adduct**: -H<br>**Expected mass (Da)**: 550.1653<br>**Observed mass (Da)**: 550.1654<br>**Predicted CCS (Å^2)**: 231.64<br>**Observed CCS (Å^2)**: 232.77<br>**Δ CCS (%)**: 0.5<br>**Observed drift time (ms)**: 8.03<br>**Observed retention time (min)**: 3.2	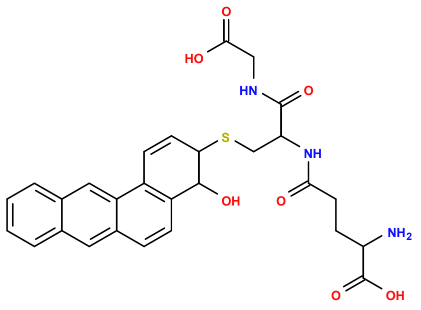

`SMILES: O=C(NC(C(=O)NCC(=O)O)CSC1C=Cc2c(C1O)ccc1c2cc2ccccc2c1)CCC(C(=O)O)N` mol

Mass spectra:

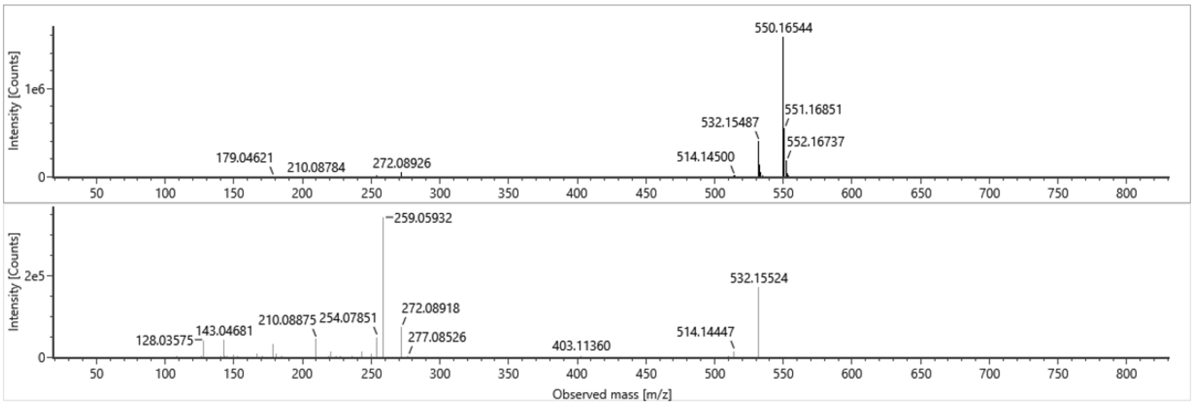

Low energy (top) and high energy (bottom) mass spectra

Benz(a)anthracene glutathione I B

Compound data	Proposed structure
**Conjugate**: glutathione<br>**Treatment**: benz(a)anthracene<br>**Formula**: $C_{28}H_{29}N_3O_7S$<br>**Neutral mass (Da)**: 551.1726<br>**Adduct**: -H<br>**Expected mass (Da)**: 550.1653<br>**Observed mass (Da)**: 550.1656<br>**Predicted CCS (Å^2)**: 231.53<br>**Observed CCS (Å^2)**: 227.05<br>**Δ CCS (%)**: -1.9<br>**Observed drift time (ms)**: 7.81<br>**Observed retention time (min)**: 2.5	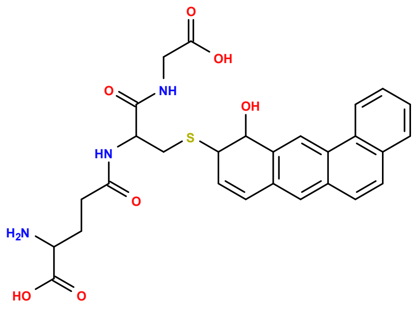

`SMILES: O=C(NC(C(=O)NCC(=O)O)CSC1C=Cc2c(C1O)cc1c(c2)ccc2c1cccc2)CCC(C(=O)O)N` mol

Mass spectra:

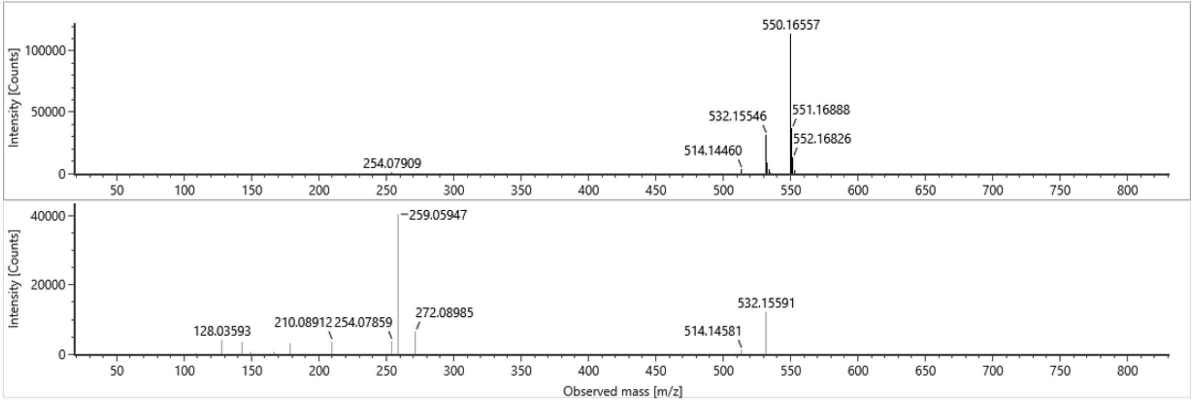

Low energy (top) and high energy (bottom) mass spectra

Benz(a)anthracene glutathione II

Compound data	Proposed structure
**Conjugate**: glutathione<br>**Treatment**: benz(a)anthracene<br>**Formula**: $C_{28}H_{29}N_3O_8S$<br>**Neutral mass (Da)**: 567.1675<br>**Adduct**: -H<br>**Expected mass (Da)**: 566.1603<br>**Observed mass (Da)**: 566.1601<br>**Predicted CCS (Å^2)**: 234.52<br>**Observed CCS (Å^2)**: 235.78<br>**Δ CCS (%)**: 0.5<br>**Observed drift time (ms)**: 8.15<br>**Observed retention time (min)**: 2.2	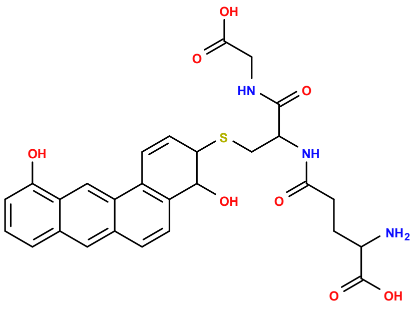

`SMILES: O=C(NC(C(=O)NCC(=O)O)CSC1C=Cc2c(C1O)ccc1c2cc2c(O)cccc2c1)CCC(C(=O)O)N` mol

Mass spectra:

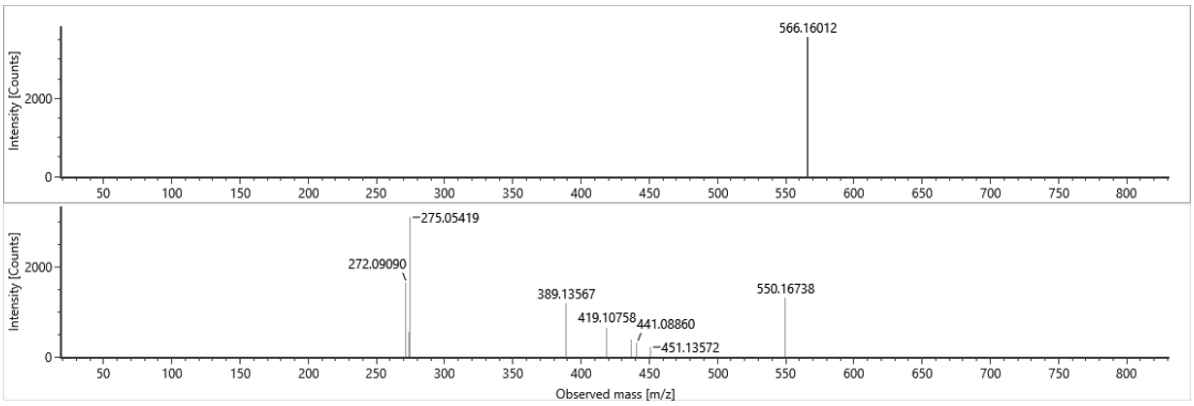

Low energy (top) and high energy (bottom) mass spectra

Benzo(a)pyrene cysteinylglycine A

Compound data	Proposed structure
**Conjugate**: cysteinylglycine<br>**Treatment**: benzo(a)pyrene<br>**Formula**: $C_{25}H_{24}N_2O_6S$<br>**Neutral mass (Da)**: 480.1355<br>**Adduct**: -H<br>**Expected mass (Da)**: 479.1282<br>**Observed mass (Da)**: 479.1288<br>**Predicted CCS (Å^2)**: 213.38<br>**Observed CCS (Å^2)**: 211.86<br>**Δ CCS (%)**: -0.7<br>**Observed drift time (ms)**: 7.21<br>**Observed retention time (min)**: 4.2	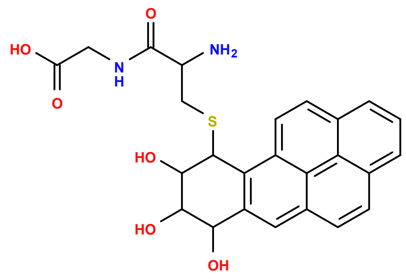

`SMILES: OC(=O)CNC(=O)C(CSC1C(O)C(O)C(c2c1c1ccc3c4c1c(c2)ccc4ccc3)O)N` mol

Mass spectra:

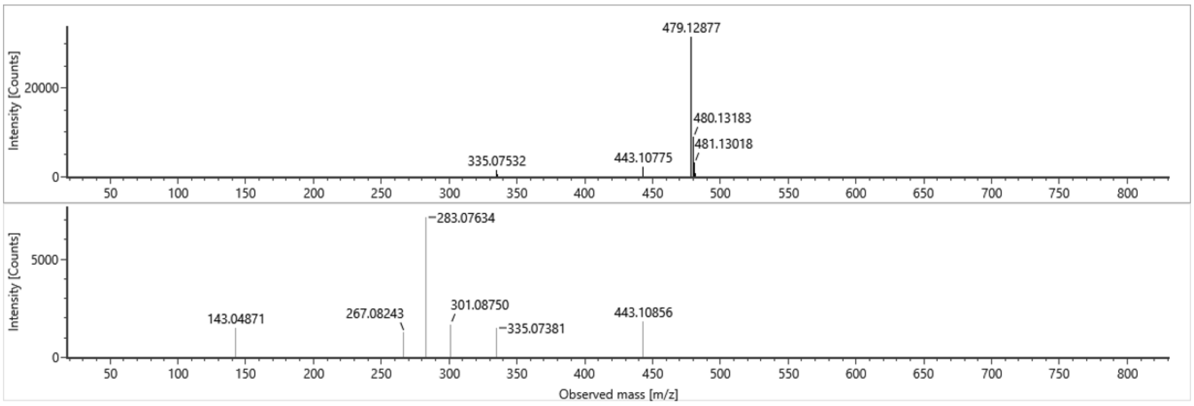

Low energy (top) and high energy (bottom) mass spectra

Benzo(a)pyrene cysteinylglycine B

Compound data	Proposed structure
**Conjugate**: cysteinylglycine<br>**Treatment**: benzo(a)pyrene<br>**Formula**: $C_{25}H_{24}N_2O_6S$<br>**Neutral mass (Da)**: 480.1355<br>**Adduct**: -H<br>**Expected mass (Da)**: 479.1282<br>**Observed mass (Da)**: 479.1288<br>**Predicted CCS (Å^2)**: 214.66<br>**Observed CCS (Å^2)**: 213.66<br>**Δ CCS (%)**: -0.5<br>**Observed drift time (ms)**: 7.28<br>**Observed retention time (min)**: 5.2	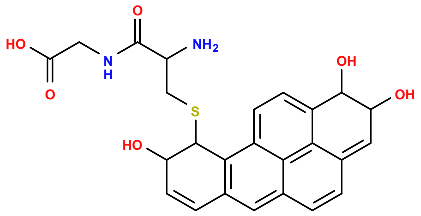

SMILES: `OC(=O)CNC(=O)C(CSC1C(O)C=Cc2c1c1ccc3c4c1c(c2)ccc4=CC(C3O)O)N` mol

Mass spectra:

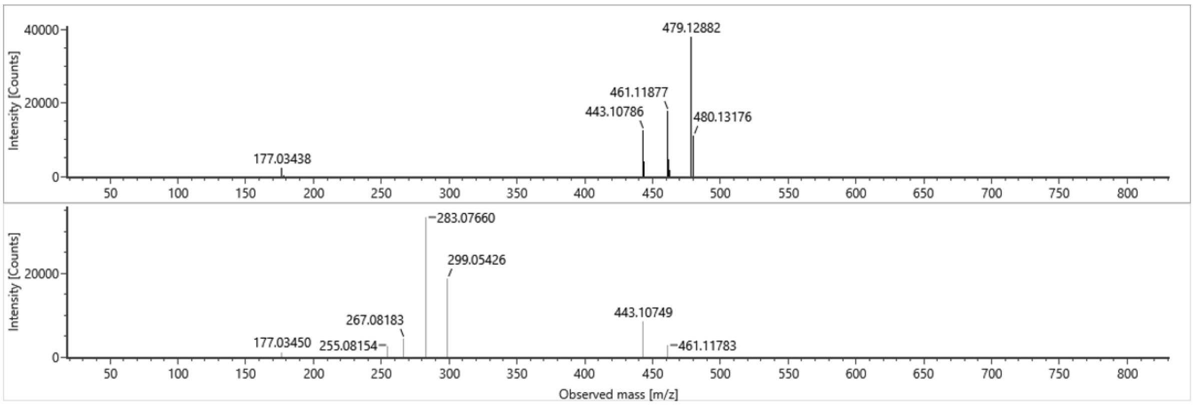

Low energy (top) and high energy (bottom) mass spectra

Chrysene cysteine

Compound data	Proposed structure
**Conjugate**: cysteine<br>**Treatment**: chrysene<br>**Formula**: $C_{21}H_{21}NO_5S$<br>**Neutral mass (Da)**: 399.1140<br>**Adduct**: -H<br>**Expected mass (Da)**: 398.1068<br>**Observed mass (Da)**: 398.1076<br>**Predicted CCS (Å^2)**: 199.66<br>**Observed CCS (Å^2)**: 194.41<br>**Δ CCS (%)**: -2.6<br>**Observed drift time (ms)**: 6.49<br>**Observed retention time (min)**: 4.1	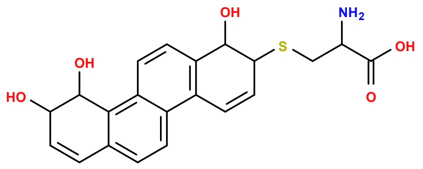

`SMILES: OC(=O)C(CSC1C=Cc2c(C1O)ccc1c2ccc2c1C(O)C(C=C2)O)N` mol

Mass spectra:

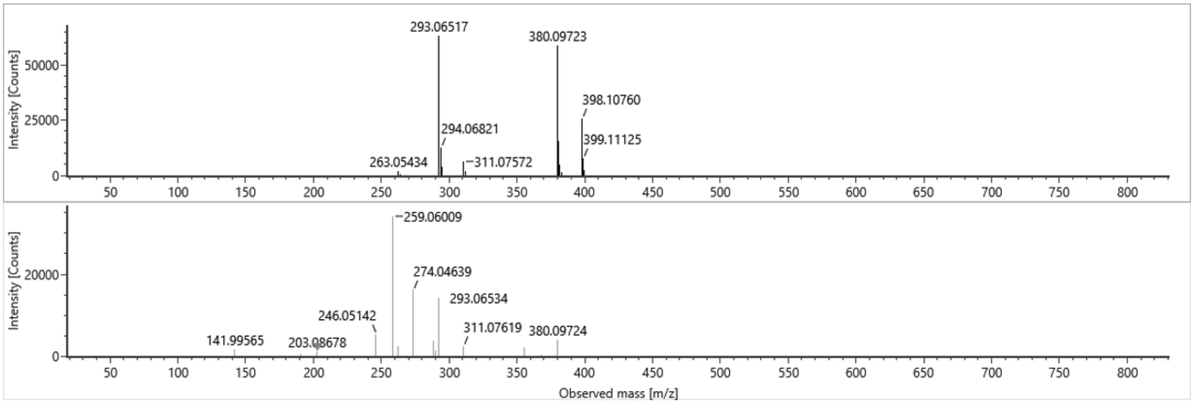

Low energy (top) and high energy (bottom) mass spectra

Chrysene cysteinylglycine I A

Compound data	Proposed structure
**Conjugate**: cysteinylglycine<br>**Treatment**: chrysene<br>**Formula**: $C_{23}H_{20}N_2O_3S$<br>**Neutral mass (Da)**: 404.1195<br>**Adduct**: -H<br>**Expected mass (Da)**: 403.1122<br>**Observed mass (Da)**: 403.1128<br>**Predicted CCS (Å^2)**: 200.23<br>**Observed CCS (Å^2)**: 202.05<br>**Δ CCS (%)**: 0.9<br>**Observed drift time (ms)**: 6.81<br>**Observed retention time (min)**: 4.8	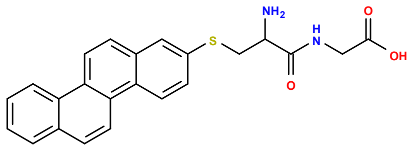

`SMILES: OC(=O)CNC(=O)C(CSc1ccc2c(c1)ccc1c2ccc2c1cccc2)N` mol

Mass spectra:

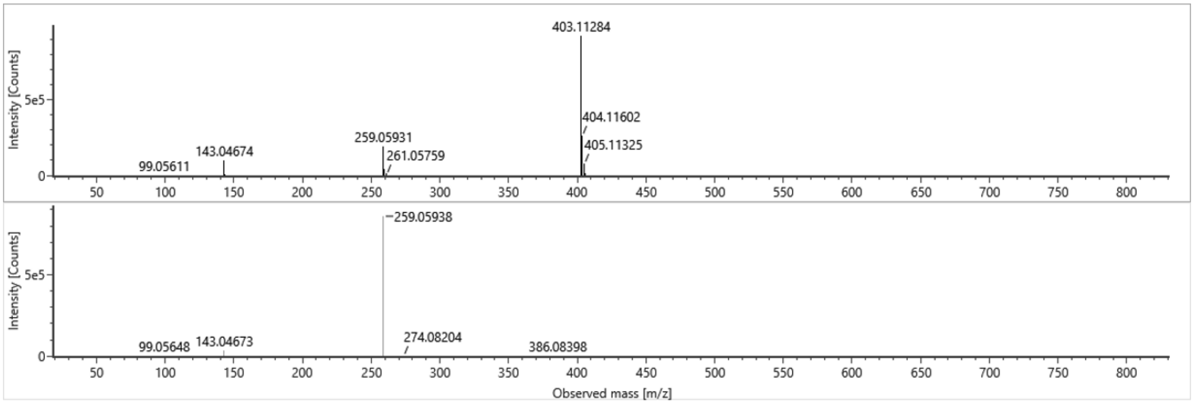

Low energy (top) and high energy (bottom) mass spectra

Chrysene cysteinylglycine I B

Compound data	Proposed structure
**Conjugate**: cysteinylglycine<br>**Treatment**: chrysene<br>**Formula**: $C_{23}H_{20}N_2O_3S$<br>**Neutral mass (Da)**: 404.1195<br>**Adduct**: -H<br>**Expected mass (Da)**: 403.1122<br>**Observed mass (Da)**: 403.1127<br>**Predicted CCS (Å^2)**: 198.84<br>**Observed CCS (Å^2)**: 204.83<br>**Δ CCS (%)**: 3.0<br>**Observed drift time (ms)**: 6.92<br>**Observed retention time (min)**: 5.8	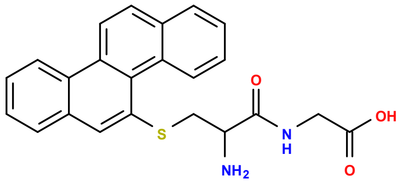

`SMILES: OC(=O)CNC(=O)C(CSc1cc2ccccc2c2c1c1ccccc1cc2)N` mol

Mass spectra:

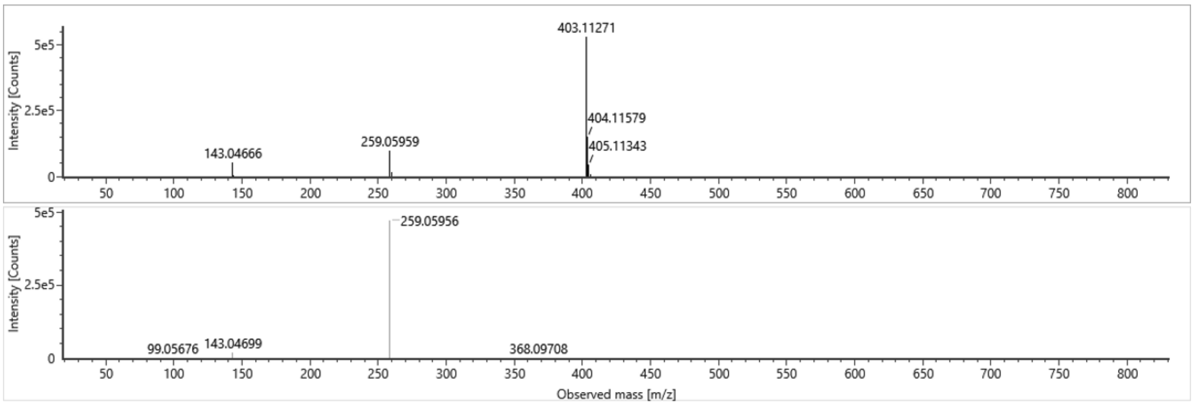

Low energy (top) and high energy (bottom) mass spectra

Chrysene cysteinylglycine II

Compound data	Proposed structure
**Conjugate**: cysteinylglycine<br>**Treatment**: chrysene<br>**Formula**: $C_{23}H_{24}N_2O_6S$<br>**Neutral mass (Da)**: 456.1355<br>**Adduct**: -H<br>**Expected mass (Da)**: 455.1282<br>**Observed mass (Da)**: 455.1290<br>**Predicted CCS (Å^2)**: 208.42<br>**Observed CCS (Å^2)**: 208.01<br>**Δ CCS (%)**: -0.2<br>**Observed drift time (ms)**: 7.04<br>**Observed retention time (min)**: 3.9	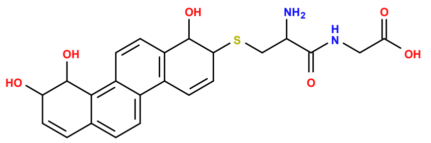

`SMILES: OC(=O)CNC(=O)C(CSC1C=Cc2c(C1O)ccc1c2ccc2c1C(O)C(C=C2)O)N` mol

Mass spectra:

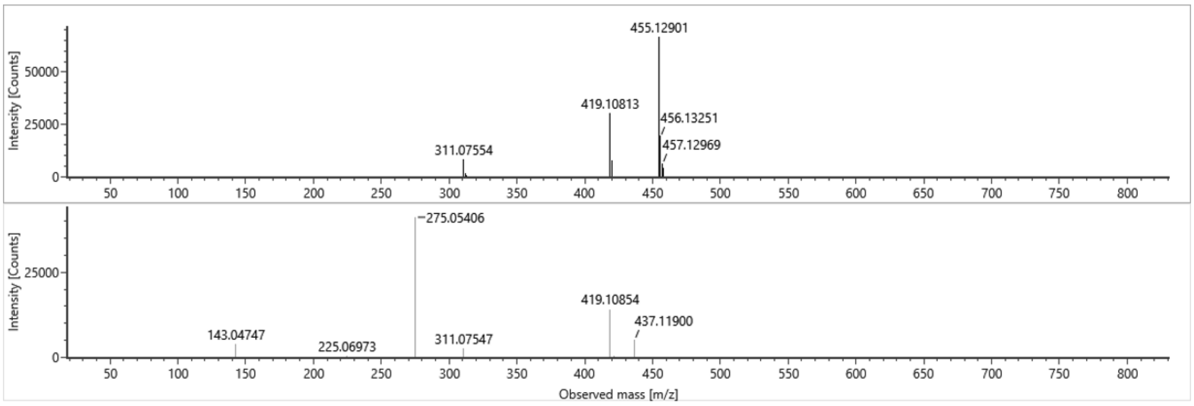

Low energy (top) and high energy (bottom) mass spectra

Chrysene glutathione I A

Compound data	Proposed structure
**Conjugate**: glutathione<br>**Treatment**: chrysene<br>**Formula**: $C_{28}H_{29}N_3O_7S$<br>**Neutral mass (Da)**: 551.1726<br>**Adduct**: -H<br>**Expected mass (Da)**: 550.1653<br>**Observed mass (Da)**: 550.1659<br>**Predicted CCS (Å^2)**: 231.64<br>**Observed CCS (Å^2)**: 230.76<br>**Δ CCS (%)**: -0.4<br>**Observed drift time (ms)**: 7.95<br>**Observed retention time (min)**: 2.3	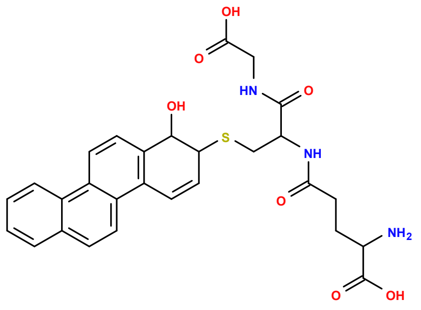

`SMILES: O=C(NC(C(=O)NCC(=O)O)CSC1C=Cc2c(C1O)ccc1c2ccc2c1cccc2)CCC(C(=O)O)N` mol

Mass spectra:

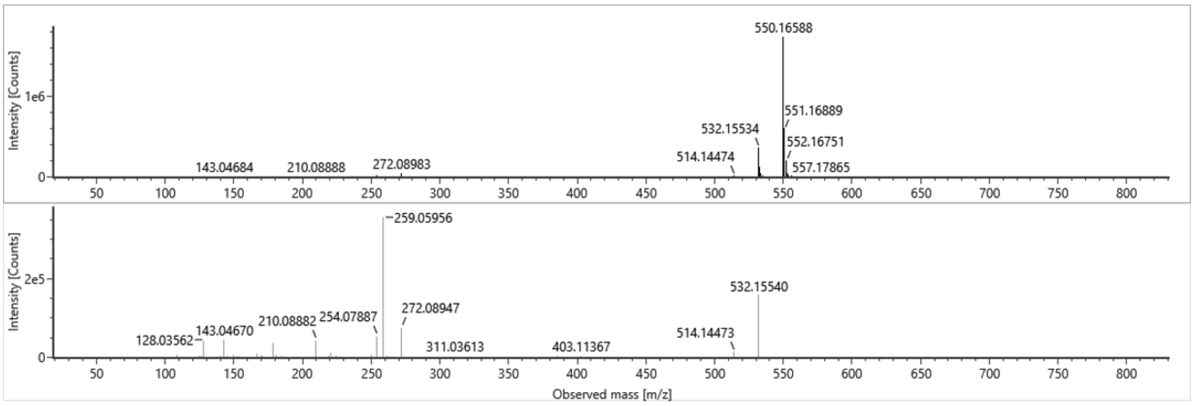

Low energy (top) and high energy (bottom) mass spectra

Chrysene glutathione I B

Compound data	Proposed structure
**Conjugate**: glutathione<br>**Treatment**: chrysene<br>**Formula**: $C_{28}H_{29}N_3O_7S$<br>**Neutral mass (Da)**: 551.1726<br>**Adduct**: -H<br>**Expected mass (Da)**: 550.1653<br>**Observed mass (Da)**: 550.1662<br>**Predicted CCS (Å^2)**: 230.66<br>**Observed CCS (Å^2)**: 232.71<br>**Δ CCS (%)**: 0.9<br>**Observed drift time (ms)**: 8.03<br>**Observed retention time (min)**: 2.9	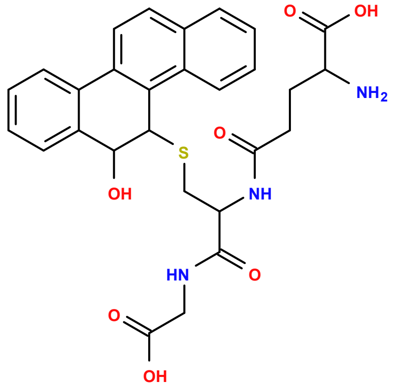

`SMILES: O=C(NC(C(=O)NCC(=O)O)CSC1C(O)c2ccccc2-c2c1c1ccccc1cc2)CCC(C(=O)O)N` mol

Mass spectra:

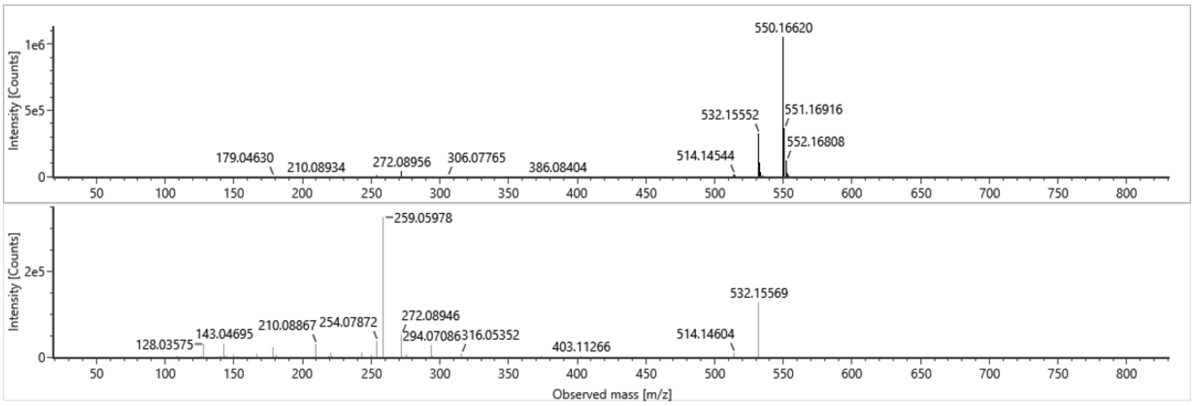

Low energy (top) and high energy (bottom) mass spectra

Chrysene glutathione II

<table>
<tr><th>Compound data</th><th>Proposed structure</th></tr>
<tr><td>

Conjugate: glutathione
Treatment: chrysene
Formula: $C_{28}H_{29}N_3O_8S$
Neutral mass (Da): 567.1675
Adduct: -H
Expected mass (Da): 566.1603
Observed mass (Da): 566.1609
Predicted CCS (Å^2): 234.50
Observed CCS (Å^2): 221.02
Δ CCS (%): -5.7
Observed drift time (ms): 7.58
Observed retention time (min): 1.5

</td><td>

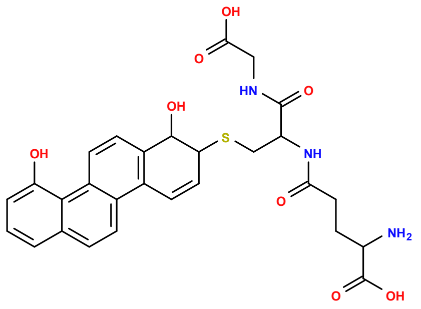

</td></tr>
</table>

`SMILES: O=C(NC(C(=O)NCC(=O)O)CSC1C=Cc2c(C1O)ccc1c2ccc2c1c(O)ccc2)CCC(C(=O)O)N` mol

Mass spectra:

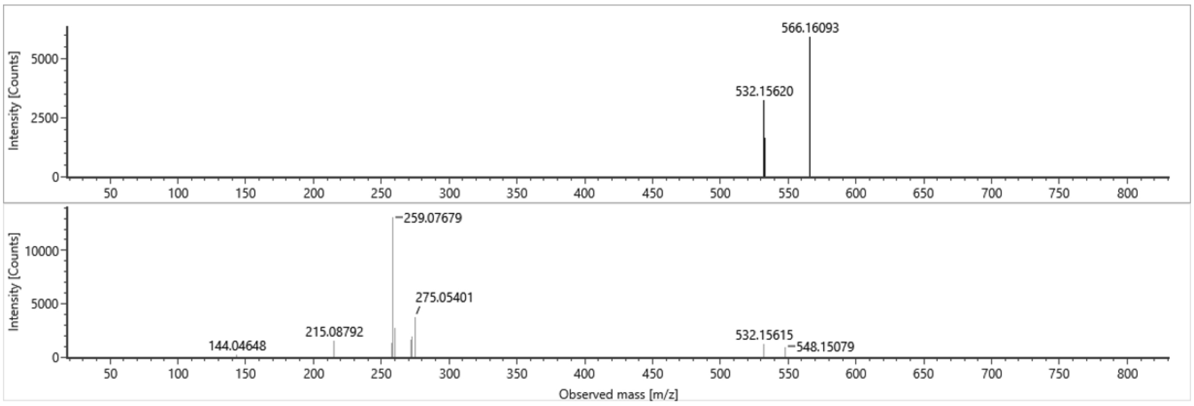

Low energy (top) and high energy (bottom) mass spectra

Chrysene mercapturic acid

Compound data	Proposed structure
**Conjugate**: mercapturic<br>**Treatment**: chrysene<br>**Formula**: $C_{23}H_{19}NO_3S$<br>**Neutral mass (Da)**: 389.1086<br>**Adduct**: -H<br>**Expected mass (Da)**: 388.1013<br>**Observed mass (Da)**: 388.1020<br>**Predicted CCS (Å^2)**: 198.06<br>**Observed CCS (Å^2)**: 204.22<br>**Δ CCS (%)**: 3.1<br>**Observed drift time (ms)**: 6.89<br>**Observed retention time (min)**: 4.5	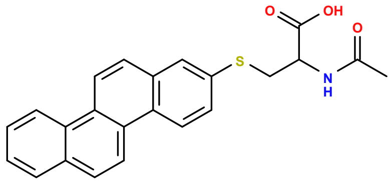

`SMILES: CC(=O)NC(C(=O)O)CSc1ccc2c(c1)ccc1c2ccc2c1cccc2` mol

Mass spectra:

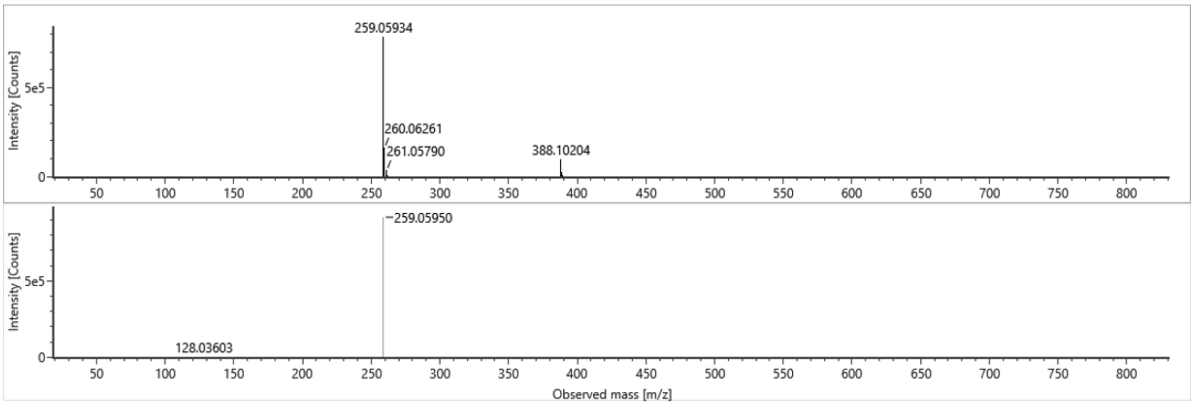

Low energy (top) and high energy (bottom) mass spectra

Dibenz(a,h)anthracene glutathione I A

Compound data	Proposed structure
**Conjugate**: glutathione<br>**Treatment**: dibenz(a,h)anthracene<br>**Formula**: $C_{32}H_{31}N_3O_7S$<br>**Neutral mass (Da)**: 601.1883<br>**Adduct**: -H<br>**Expected mass (Da)**: 600.1810<br>**Observed mass (Da)**: 600.1811<br>**Predicted CCS (Å^2)**: 245.02<br>**Observed CCS (Å^2)**: 243.18<br>**Δ CCS (%)**: -0.7<br>**Observed drift time (ms)**: 8.43<br>**Observed retention time (min)**: 5.8	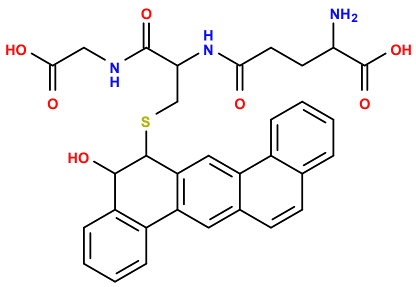

`SMILES: O=C(NC(C(=O)NCC(=O)O)CSC1C(O)c2ccccc2-c2c1cc1c(c2)ccc2c1cccc2)CCC(C(=O)O)N` mol

Mass spectra:

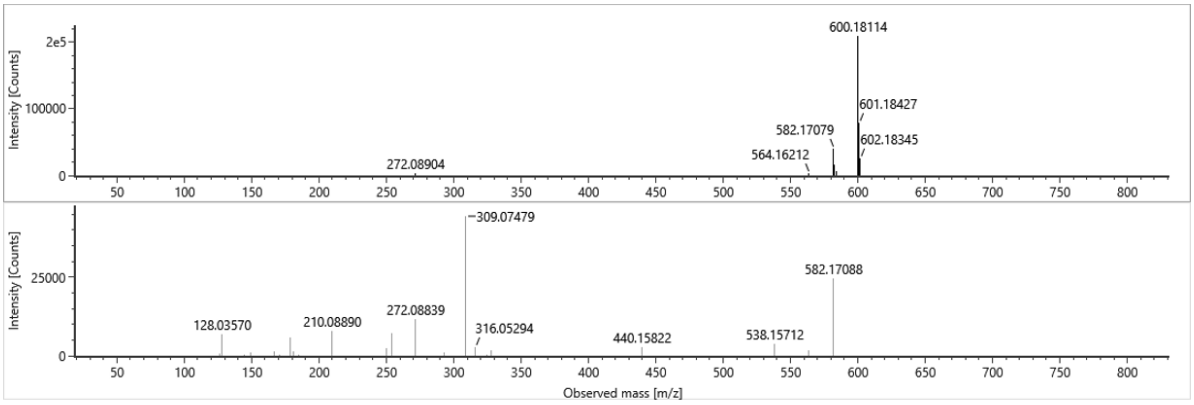

Low energy (top) and high energy (bottom) mass spectra

Dibenz(a,h)anthracene glutathione I B

Compound data	Proposed structure
**Conjugate**: glutathione<br>**Treatment**: dibenz(a,h)anthracene<br>**Formula**: $C_{32}H_{31}N_3O_7S$<br>**Neutral mass (Da)**: 601.1883<br>**Adduct**: -H<br>**Expected mass (Da)**: 600.1810<br>**Observed mass (Da)**: 600.1811<br>**Predicted CCS (Å^2)**: 246.02<br>**Observed CCS (Å^2)**: 248.13<br>**Δ CCS (%)**: 0.9<br>**Observed drift time (ms)**: 8.63<br>**Observed retention time (min)**: 6.7	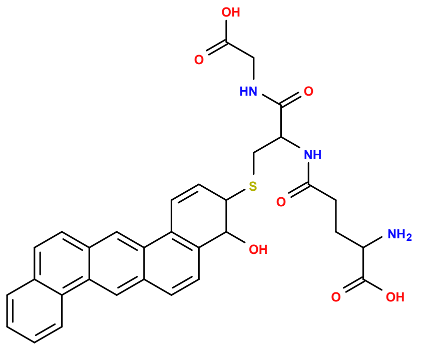

`SMILES: O=C(NC(C(=O)NCC(=O)O)CSC1C=Cc2c(C1O)ccc1c2cc2ccc3c(c2c1)cccc3)CCC(C(=O)O)N` mol

Mass spectra:

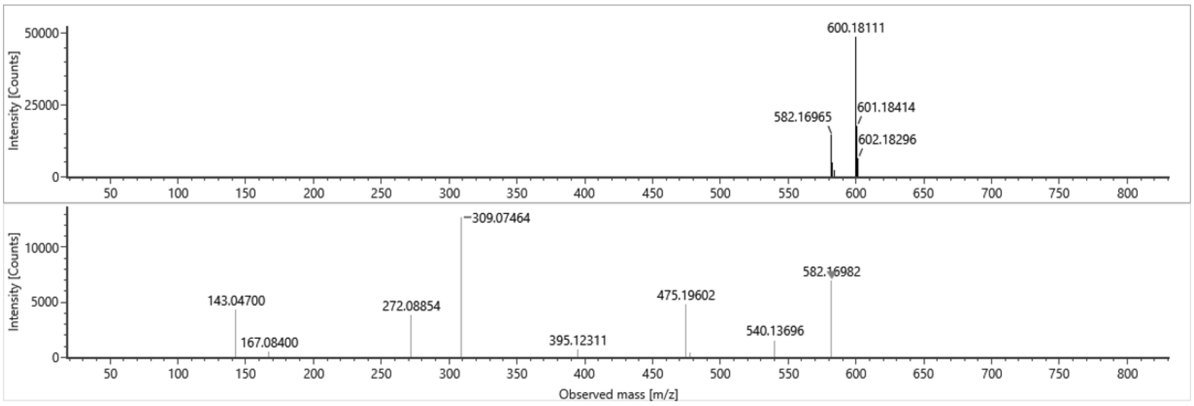

Low energy (top) and high energy (bottom) mass spectra

Dibenz(a,h)anthracene glutathione II

Compound data	Proposed structure
**Conjugate**: glutathione<br>**Treatment**: dibenz(a,h)anthracene<br>**Formula**: $C_{32}H_{33}N_3O_9S$<br>**Neutral mass (Da)**: 635.1938<br>**Adduct**: -H<br>**Expected mass (Da)**: 634.1865<br>**Observed mass (Da)**: 634.1866<br>**Predicted CCS (Å^2)**: 253.09<br>**Observed CCS (Å^2)**: 248.58<br>**Δ CCS (%)**: -1.8<br>**Observed drift time (ms)**: 8.63<br>**Observed retention time (min)**: 3.7	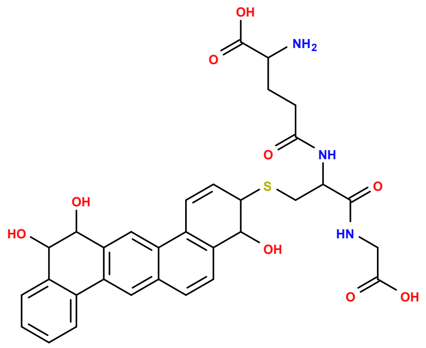

SMILES: O=C(NC(C(=O)NCC(=O)O)CSC1C=Cc2c(C1O)ccc1c2cc2C(O)C(O)c3c(-c2c1)cccc3)CCC(C(=O)O)N mol

Mass spectra:

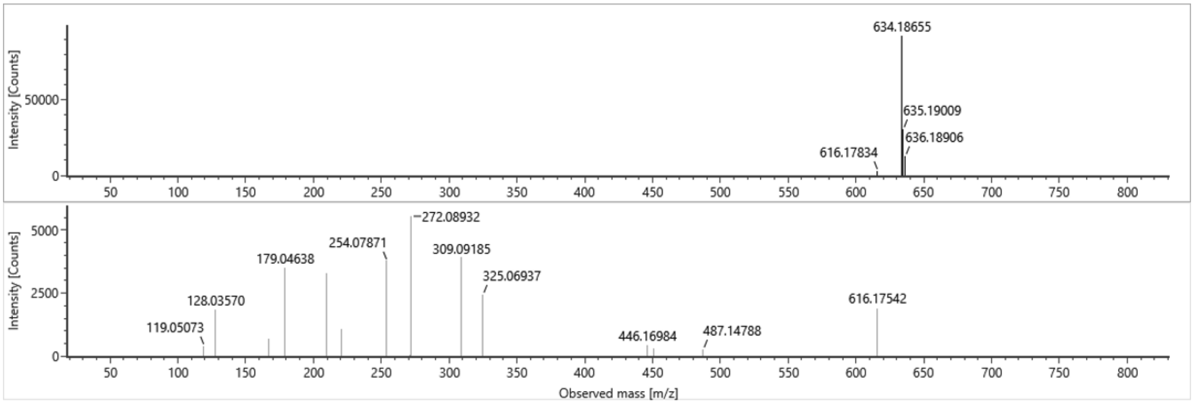

Low energy (top) and high energy (bottom) mass spectra

Phenanthrene cysteinylglycine

Compound data	Proposed structure
**Conjugate**: cysteinylglycine<br>**Treatment**: phenanthrene<br>**Formula**: $C_{19}H_{18}N_2O_3S$<br>**Neutral mass (Da)**: 354.1038<br>**Adduct**: -H<br>**Expected mass (Da)**: 353.0965<br>**Observed mass (Da)**: 353.0974<br>**Predicted CCS (Å^2)**: 183.91<br>**Observed CCS (Å^2)**: 189.75<br>**Δ CCS (%)**: 3.2<br>**Observed drift time (ms)**: 6.30<br>**Observed retention time (min)**: 2.1	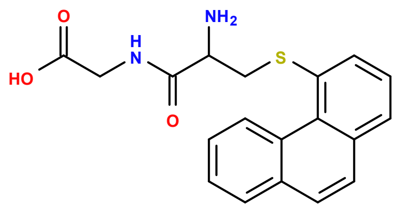

SMILES: OC(=O)CNC(=O)C(CSc1cccc2c1c1ccccc1cc2)N mol

Mass spectra:

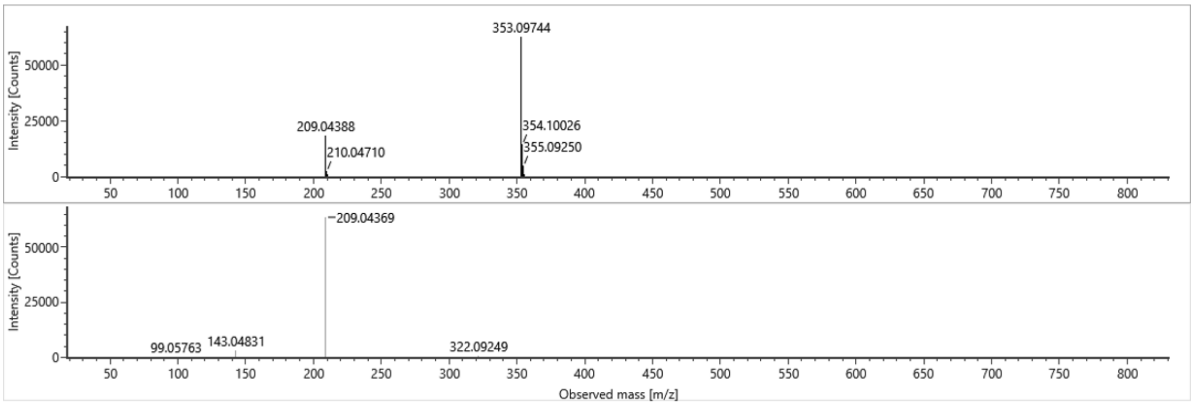

Low energy (top) and high energy (bottom) mass spectra

Phenanthrene glutathione

Compound data	Proposed structure
**Conjugate**: glutathione<br>**Treatment**: phenanthrene<br>**Formula**: $C_{24}H_{23}N_3O_8S$<br>**Neutral mass (Da)**: 513.1206<br>**Adduct**: -H<br>**Expected mass (Da)**: 512.1133<br>**Observed mass (Da)**: 512.1139<br>**Predicted CCS (Å^2)**: 220.27<br>**Observed CCS (Å^2)**: 212.65<br>**Δ CCS (%)**: -3.5<br>**Observed drift time (ms)**: 7.24<br>**Observed retention time (min)**: 2.9	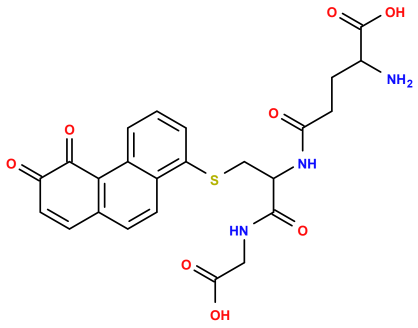

`SMILES: O=C(NC(C(=O)NCC(=O)O)CSc1cccc2c1ccc1c2C(=O)C(=O)C=C1)CCC(C(=O)O)N` mol

Mass spectra:

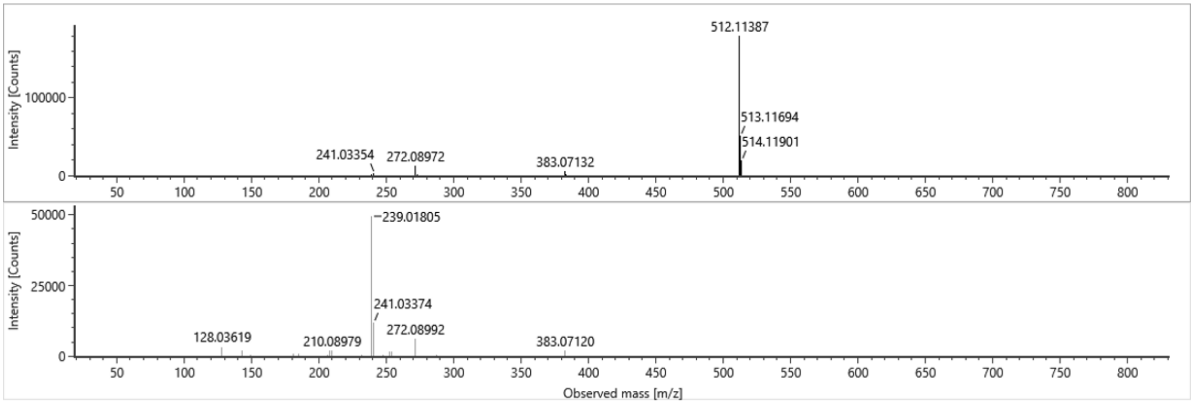

Low energy (top) and high energy (bottom) mass spectra

Mol files

1,4-Dimethylphenanthrene cysteinylglycine A

```
1,4-dimethylphenanthrene cysteinylglycine B.mol.sb-93f19d69-CLNE6i
  ChemDraw04012419592D

  0  0  0     0  0              0 V3000
M  V30 BEGIN CTAB
M  V30 COUNTS 27 29 0 0 0
M  V30 BEGIN ATOM
M  V30 1 C 4.279681 0.617719 0.000000 0
M  V30 2 C 4.279681 1.441344 0.000000 0
M  V30 3 C 3.566401 1.853156 0.000000 0
M  V30 4 C 2.853120 1.441344 0.000000 0
M  V30 5 C 2.853120 0.617719 0.000000 0
M  V30 6 C 2.139840 0.205906 0.000000 0
M  V30 7 C 1.426560 0.617719 0.000000 0
M  V30 8 C 0.713280 0.205906 0.000000 0
M  V30 9 C 0.713280 -0.617719 0.000000 0
M  V30 10 C 1.426560 -1.029531 0.000000 0
M  V30 11 C 2.139840 -0.617719 0.000000 0
M  V30 12 C 2.853120 -1.029531 0.000000 0
M  V30 13 C 3.566401 -0.617719 0.000000 0
M  V30 14 C 3.566401 0.205906 0.000000 0
M  V30 15 C 2.139840 1.853156 0.000000 0
M  V30 16 C 4.992961 0.205906 0.000000 0
M  V30 17 C -4.279681 -1.029531 0.000000 0
M  V30 18 N -2.853121 -1.029531 0.000000 0
M  V30 19 C -3.566401 -0.617719 0.000000 0
M  V30 20 O -4.279681 -1.853156 0.000000 0
M  V30 21 O -4.992961 -0.617719 0.000000 0
M  V30 22 C -2.139841 -0.617719 0.000000 0
M  V30 23 N -1.426560 -1.853156 0.000000 0
M  V30 24 C -1.426560 -1.029531 0.000000 0
M  V30 25 C -0.713280 -0.617719 0.000000 0
M  V30 26 S 0.000000 -1.029531 0.000000 0
M  V30 27 O -2.139841 0.205906 0.000000 0
M  V30 END ATOM
M  V30 BEGIN BOND
M  V30 1 1 1 2
M  V30 2 2 2 3
M  V30 3 1 3 4
M  V30 4 2 4 5
M  V30 5 1 5 6
M  V30 6 2 6 7
M  V30 7 1 7 8
M  V30 8 2 8 9
M  V30 9 1 9 10
M  V30 10 2 10 11
M  V30 11 1 6 11
M  V30 12 1 11 12
M  V30 13 2 12 13
M  V30 14 1 13 14
M  V30 15 2 1 14
M  V30 16 1 5 14
M  V30 17 1 4 15
M  V30 18 1 1 16
M  V30 19 1 18 19
M  V30 20 1 17 19
M  V30 21 2 17 20
M  V30 22 1 17 21
M  V30 23 1 22 24
M  V30 24 1 24 25
M  V30 25 1 25 26
M  V30 26 2 22 27
M  V30 27 1 18 22
M  V30 28 1 24 23
M  V30 29 1 26 9
M  V30 END BOND
M  V30 END CTAB
M  END
```

1,4-Dimethylphenanthrene cysteinylglycine B

```
1,4-dimethylphenanthrene cysteinylglycine A.mol.sb-cf5d4b74-u5Gj9n
  ChemDraw04102408092D

  0  0  0     0  0              0 V3000
M  V30 BEGIN CTAB
M  V30 COUNTS 27 29 0 0 0
M  V30 BEGIN ATOM
M  V30 1 C -0.713280 0.617719 0.000000 0
M  V30 2 C -0.713280 1.441344 0.000000 0
M  V30 3 C -1.426561 1.853156 0.000000 0
M  V30 4 C -2.139841 1.441344 0.000000 0
M  V30 5 C -2.139841 0.617719 0.000000 0
M  V30 6 C -2.853121 0.205906 0.000000 0
M  V30 7 C -3.566401 0.617719 0.000000 0
M  V30 8 C -4.279681 0.205906 0.000000 0
M  V30 9 C -4.279681 -0.617719 0.000000 0
M  V30 10 C -3.566401 -1.029531 0.000000 0
M  V30 11 C -2.853121 -0.617719 0.000000 0
M  V30 12 C -2.139841 -1.029531 0.000000 0
M  V30 13 C -1.426561 -0.617719 0.000000 0
M  V30 14 C -1.426561 0.205906 0.000000 0
M  V30 15 C -2.853121 1.853156 0.000000 0
M  V30 16 C -0.000000 0.205906 0.000000 0
M  V30 17 C 3.566401 -1.029531 0.000000 0
M  V30 18 N 2.139841 -1.029531 0.000000 0
M  V30 19 C 2.853121 -0.617719 0.000000 0
M  V30 20 O 3.566401 -1.853156 0.000000 0
M  V30 21 O 4.279681 -0.617719 0.000000 0
M  V30 22 C 1.426560 -0.617719 0.000000 0
M  V30 23 N 0.713280 -1.853156 0.000000 0
M  V30 24 C 0.713280 -1.029531 0.000000 0
M  V30 25 C -0.000000 -0.617719 0.000000 0
M  V30 26 S -0.713280 -1.029531 0.000000 0
M  V30 27 O 1.426560 0.205906 0.000000 0
M  V30 END ATOM
M  V30 BEGIN BOND
M  V30 1 1 1 2
M  V30 2 2 2 3
M  V30 3 1 3 4
M  V30 4 2 4 5
M  V30 5 1 5 6
M  V30 6 2 6 7
M  V30 7 1 7 8
M  V30 8 2 8 9
M  V30 9 1 9 10
M  V30 10 2 10 11
M  V30 11 1 6 11
M  V30 12 1 11 12
M  V30 13 2 12 13
M  V30 14 1 13 14
M  V30 15 2 1 14
M  V30 16 1 5 14
M  V30 17 1 4 15
M  V30 18 1 1 16
M  V30 19 1 18 19
M  V30 20 1 17 19
M  V30 21 2 17 20
M  V30 22 1 17 21
M  V30 23 1 22 24
M  V30 24 1 24 25
M  V30 25 1 25 26
M  V30 26 2 22 27
M  V30 27 1 18 22
M  V30 28 1 24 23
M  V30 29 1 26 13
M  V30 END BOND
M  V30 END CTAB
M  END
```

1,4-Dimethylphenanthrene glutathione I

```
1,4-dimethylphenanthrene glutathione I B.mol.sb-cf5d4b74-3DNFDk
  ChemDraw04102408122D

  0  0  0     0  0              0 V3000
M  V30 BEGIN CTAB
M  V30 COUNTS 36 38 0 0 0
M  V30 BEGIN ATOM
M  V30 1 C 3.209761 3.088594 0.000000 0
M  V30 2 C 3.209761 3.912219 0.000000 0
M  V30 3 C 2.496481 4.324031 0.000000 0
M  V30 4 C 1.783200 3.912219 0.000000 0
M  V30 5 C 1.783200 3.088594 0.000000 0
M  V30 6 C 1.069920 2.676781 0.000000 0
M  V30 7 C 0.356640 3.088594 0.000000 0
M  V30 8 C -0.356640 2.676781 0.000000 0
M  V30 9 C -0.356640 1.853156 0.000000 0
M  V30 10 C 0.356640 1.441343 0.000000 0
M  V30 11 C 1.069920 1.853156 0.000000 0
M  V30 12 C 1.783200 1.441343 0.000000 0
M  V30 13 C 2.496481 1.853156 0.000000 0
M  V30 14 C 2.496481 2.676781 0.000000 0
M  V30 15 C 1.069920 4.324031 0.000000 0
M  V30 16 C 3.923041 2.676781 0.000000 0
M  V30 17 O -3.209761 0.205906 0.000000 0
M  V30 18 C -2.496481 0.617719 0.000000 0
M  V30 19 N -2.496481 1.441343 0.000000 0
M  V30 20 C -3.209761 1.853156 0.000000 0
M  V30 21 C -3.209761 2.676781 0.000000 0
M  V30 22 O -2.496481 3.088594 0.000000 0
M  V30 23 O -3.923041 3.088594 0.000000 0
M  V30 24 C -1.783201 0.205906 0.000000 0
M  V30 25 C -1.069920 0.617719 0.000000 0
M  V30 26 S -1.069920 1.441343 0.000000 0
M  V30 27 N -1.783201 -0.617719 0.000000 0
M  V30 28 C -1.069920 -1.029531 0.000000 0
M  V30 29 C -1.069920 -1.853156 0.000000 0
M  V30 30 C -0.356640 -2.264969 0.000000 0
M  V30 31 C -0.356640 -3.088594 0.000000 0
M  V30 32 N -1.069920 -3.500406 0.000000 0
M  V30 33 C 0.356640 -3.500406 0.000000 0
M  V30 34 O 0.356640 -4.324031 0.000000 0
M  V30 35 O 1.069920 -3.088594 0.000000 0
M  V30 36 O -0.356640 -0.617719 0.000000 0
M  V30 END ATOM
M  V30 BEGIN BOND
M  V30 1 1 1 2
M  V30 2 2 2 3
M  V30 3 1 3 4
M  V30 4 2 4 5
M  V30 5 1 5 6
M  V30 6 2 6 7
M  V30 7 1 7 8
M  V30 8 2 8 9
M  V30 9 1 9 10
M  V30 10 2 10 11
M  V30 11 1 6 11
M  V30 12 1 11 12
M  V30 13 2 12 13
M  V30 14 1 13 14
M  V30 15 2 1 14
M  V30 16 1 5 14
M  V30 17 1 4 15
M  V30 18 1 1 16
M  V30 19 2 17 18
M  V30 20 1 18 19
M  V30 21 1 19 20
M  V30 22 1 20 21
M  V30 23 1 21 22
M  V30 24 2 21 23
M  V30 25 1 18 24
M  V30 26 1 24 25
M  V30 27 1 25 26
M  V30 28 1 24 27
M  V30 29 1 27 28
M  V30 30 1 28 29
M  V30 31 1 29 30
M  V30 32 1 30 31
M  V30 33 1 31 32
M  V30 34 1 31 33
M  V30 35 1 33 34
M  V30 36 2 33 35
M  V30 37 2 28 36
M  V30 38 1 9 26
M  V30 END BOND
M  V30 END CTAB
M  END
```

1,4-Dimethylphenanthrene glutathione II

```
1,4-dimethylphenanthrene glutathione II A.mol.sb-cf5d4b74-fwhfAM
  ChemDraw04102411362D

  0  0  0     0  0              0 V3000
M  V30 BEGIN CTAB
M  V30 COUNTS 37 39 0 0 0
M  V30 BEGIN ATOM
M  V30 1 C 3.215119 3.093750 0.000000 0
M  V30 2 C 3.215119 3.918749 0.000000 0
M  V30 3 C 2.500648 4.331250 0.000000 0
M  V30 4 C 1.786178 3.918749 0.000000 0
M  V30 5 C 1.786178 3.093750 0.000000 0
M  V30 6 C 1.071707 2.681250 0.000000 0
M  V30 7 C 0.357235 3.093750 0.000000 0
M  V30 8 C -0.357235 2.681250 0.000000 0
M  V30 9 C -0.357236 1.856250 0.000000 0
M  V30 10 C 0.357235 1.443750 0.000000 0
M  V30 11 C 1.071707 1.856250 0.000000 0
M  V30 12 C 1.786178 1.443750 0.000000 0
M  V30 13 C 2.500648 1.856250 0.000000 0
M  V30 14 C 2.500648 2.681250 0.000000 0
M  V30 15 C 1.071707 4.331249 0.000000 0
M  V30 16 C 3.929591 2.681250 0.000000 0
M  V30 17 S -1.071707 1.443750 0.000000 0
M  V30 18 O -3.215119 0.206250 0.000000 0
M  V30 19 C -2.500648 0.618750 0.000000 0
M  V30 20 N -2.500648 1.443750 0.000000 0
M  V30 21 C -3.215119 1.856250 0.000000 0
M  V30 22 C -3.215119 2.681250 0.000000 0
M  V30 23 O -2.500648 3.093750 0.000000 0
M  V30 24 O -3.929591 3.093750 0.000000 0
M  V30 25 C -1.786178 0.206250 0.000000 0
M  V30 26 C -1.071707 0.618750 0.000000 0
M  V30 27 N -1.786178 -0.618750 0.000000 0
M  V30 28 C -1.071707 -1.031250 0.000000 0
M  V30 29 C -1.071707 -1.856250 0.000000 0
M  V30 30 C -0.357236 -2.268750 0.000000 0
M  V30 31 C -0.357236 -3.093750 0.000000 0
M  V30 32 N -1.071707 -3.506250 0.000000 0
M  V30 33 C 0.357235 -3.506250 0.000000 0
M  V30 34 O 0.357235 -4.331250 0.000000 0
M  V30 35 O 1.071706 -3.093750 0.000000 0
M  V30 36 O -0.357236 -0.618750 0.000000 0
M  V30 37 O 0.357234 0.618750 0.000000 0
M  V30 END ATOM
M  V30 BEGIN BOND
M  V30 1 1 1 2
M  V30 2 2 2 3
M  V30 3 1 3 4
M  V30 4 2 4 5
M  V30 5 1 5 6
M  V30 6 1 6 7
M  V30 7 2 7 8
M  V30 8 1 8 9
M  V30 9 1 9 10
M  V30 10 1 10 11
M  V30 11 2 6 11
M  V30 12 1 11 12
M  V30 13 2 12 13
M  V30 14 1 13 14
M  V30 15 2 1 14
M  V30 16 1 5 14
M  V30 17 1 4 15
M  V30 18 1 1 16
M  V30 19 1 26 17
M  V30 20 2 18 19
M  V30 21 1 19 20
M  V30 22 1 20 21
M  V30 23 1 21 22
M  V30 24 1 22 23
M  V30 25 2 22 24
M  V30 26 1 19 25
M  V30 27 1 25 26
M  V30 28 1 25 27
M  V30 29 1 27 28
M  V30 30 1 28 29
M  V30 31 1 29 30
M  V30 32 1 30 31
M  V30 33 1 31 32
M  V30 34 1 31 33
M  V30 35 1 33 34
M  V30 36 2 33 35
M  V30 37 2 28 36
M  V30 38 1 17 9
M  V30 39 1 10 37
M  V30 END BOND
M  V30 END CTAB
M  END
```

1-Methylphenanthrene cysteinylglycine A

```
1-methylphenanthrene cysteinylglycine I A.mol.sb-cf5d4b74-P17ZcV
  ChemDraw04092421022D

  0  0  0     0  0              0 V3000
M  V30 BEGIN CTAB
M  V30 COUNTS 26 28 0 0 0
M  V30 BEGIN ATOM
M  V30 1 C -2.143413 2.887500 0.000000 0
M  V30 2 N -1.428942 1.650000 0.000000 0
M  V30 3 C -1.428942 2.475000 0.000000 0
M  V30 4 O -2.857884 2.475000 0.000000 0
M  V30 5 O -2.143413 3.712500 0.000000 0
M  V30 6 C -0.714471 1.237500 0.000000 0
M  V30 7 C -0.714471 0.412500 0.000000 0
M  V30 8 C -0.000000 -0.000000 0.000000 0
M  V30 9 O -0.000000 1.650000 0.000000 0
M  V30 10 N -1.428942 -0.000000 0.000000 0
M  V30 11 S -0.000000 -0.825000 0.000000 0
M  V30 12 C 2.143413 -2.062500 0.000000 0
M  V30 13 C 2.143414 -1.237501 0.000000 0
M  V30 14 C 1.428942 -0.825000 0.000000 0
M  V30 15 C 0.714471 -1.237500 0.000000 0
M  V30 16 C 0.714470 -2.062499 0.000000 0
M  V30 17 C -0.000000 -2.474998 0.000000 0
M  V30 18 C -0.714471 -2.062498 0.000000 0
M  V30 19 C -1.428941 -2.474998 0.000000 0
M  V30 20 C -1.428942 -3.299998 0.000000 0
M  V30 21 C -0.714471 -3.712499 0.000000 0
M  V30 22 C -0.000000 -3.299999 0.000000 0
M  V30 23 C 0.714470 -3.712500 0.000000 0
M  V30 24 C 1.428941 -3.300000 0.000000 0
M  V30 25 C 1.428941 -2.475000 0.000000 0
M  V30 26 C 2.857884 -2.475000 0.000000 0
M  V30 END ATOM
M  V30 BEGIN BOND
M  V30 1 1 2 3
M  V30 2 1 1 3
M  V30 3 2 1 4
M  V30 4 1 1 5
M  V30 5 1 2 6
M  V30 6 1 6 7
M  V30 7 1 7 8
M  V30 8 2 6 9
M  V30 9 1 7 10
M  V30 10 1 11 8
M  V30 11 1 12 13
M  V30 12 2 13 14
M  V30 13 1 14 15
M  V30 14 2 15 16
M  V30 15 1 16 17
M  V30 16 2 17 18
M  V30 17 1 18 19
M  V30 18 2 19 20
M  V30 19 1 20 21
M  V30 20 2 21 22
M  V30 21 1 17 22
M  V30 22 1 22 23
M  V30 23 2 23 24
M  V30 24 1 24 25
M  V30 25 2 12 25
M  V30 26 1 16 25
M  V30 27 1 12 26
M  V30 28 1 15 11
M  V30 END BOND
M  V30 END CTAB
M  END
```

1-Methylphenanthrene cysteinylglycine B

```
1-methylphenanthrene cysteinylglycine I B.mol.sb-cf5d4b74-Gs2mPt
  ChemDraw04092421012D

  0  0  0     0  0              0 V3000
M  V30 BEGIN CTAB
M  V30 COUNTS 26 28 0 0 0
M  V30 BEGIN ATOM
M  V30 1 C 2.496480 -2.882688 0.000000 0
M  V30 2 N 1.783200 -1.647250 0.000000 0
M  V30 3 C 1.783200 -2.470875 0.000000 0
M  V30 4 O 3.209760 -2.470875 0.000000 0
M  V30 5 O 2.496480 -3.706313 0.000000 0
M  V30 6 C 1.069920 -1.235437 0.000000 0
M  V30 7 C 1.069920 -0.411812 0.000000 0
M  V30 8 C 0.356640 0.000000 0.000000 0
M  V30 9 O 0.356640 -1.647250 0.000000 0
M  V30 10 N 1.783200 0.000000 0.000000 0
M  V30 11 S 0.356640 0.823625 0.000000 0
M  V30 12 C 0.356642 2.470876 0.000000 0
M  V30 13 C 0.356642 3.294499 0.000000 0
M  V30 14 C -0.356639 3.706313 0.000000 0
M  V30 15 C -1.069920 3.294501 0.000000 0
M  V30 16 C -1.069920 2.470876 0.000000 0
M  V30 17 C -1.783199 2.059064 0.000000 0
M  V30 18 C -2.496480 2.470877 0.000000 0
M  V30 19 C -3.209760 2.059064 0.000000 0
M  V30 20 C -3.209760 1.235439 0.000000 0
M  V30 21 C -2.496481 0.823626 0.000000 0
M  V30 22 C -1.783201 1.235437 0.000000 0
M  V30 23 C -1.069921 0.823626 0.000000 0
M  V30 24 C -0.356641 1.235437 0.000000 0
M  V30 25 C -0.356639 2.059063 0.000000 0
M  V30 26 C 1.069922 2.059064 0.000000 0
M  V30 END ATOM
M  V30 BEGIN BOND
M  V30 1 1 2 3
M  V30 2 1 1 3
M  V30 3 2 1 4
M  V30 4 1 1 5
M  V30 5 1 2 6
M  V30 6 1 6 7
M  V30 7 1 7 8
M  V30 8 2 6 9
M  V30 9 1 7 10
M  V30 10 1 11 8
M  V30 11 1 12 13
M  V30 12 2 13 14
M  V30 13 1 14 15
M  V30 14 2 15 16
M  V30 15 1 16 17
M  V30 16 2 17 18
M  V30 17 1 18 19
M  V30 18 2 19 20
M  V30 19 1 20 21
M  V30 20 2 21 22
M  V30 21 1 17 22
M  V30 22 1 22 23
M  V30 23 2 23 24
M  V30 24 1 24 25
M  V30 25 2 12 25
M  V30 26 1 16 25
M  V30 27 1 12 26
M  V30 28 1 11 24
M  V30 END BOND
M  V30 END CTAB
M  END
```

1-Methylphenanthrene glutathione I

```
1-methylphenanthrene glutathione I A.mol.sb-cf5d4b74-rYHPpq
  ChemDraw04092421112D

  0  0  0     0  0              0 V3000
M  V30 BEGIN CTAB
M  V30 COUNTS 35 37 0 0 0
M  V30 BEGIN ATOM
M  V30 1 C 2.500648 -2.062500 0.000000 0
M  V30 2 C 2.500648 -1.237500 0.000000 0
M  V30 3 C 1.786178 -0.825000 0.000000 0
M  V30 4 C 1.071707 -1.237500 0.000000 0
M  V30 5 C 1.071707 -2.062499 0.000000 0
M  V30 6 C 0.357235 -2.474999 0.000000 0
M  V30 7 C -0.357235 -2.062499 0.000000 0
M  V30 8 C -1.071706 -2.474999 0.000000 0
M  V30 9 C -1.071706 -3.299999 0.000000 0
M  V30 10 C -0.357235 -3.712500 0.000000 0
M  V30 11 C 0.357235 -3.299999 0.000000 0
M  V30 12 C 1.071707 -3.712500 0.000000 0
M  V30 13 C 1.786178 -3.300000 0.000000 0
M  V30 14 C 1.786178 -2.475000 0.000000 0
M  V30 15 C 3.215119 -2.475000 0.000000 0
M  V30 16 O -1.071706 1.650000 0.000000 0
M  V30 17 C -0.357235 1.237500 0.000000 0
M  V30 18 N 0.357236 1.650000 0.000000 0
M  V30 19 C 0.357236 2.475000 0.000000 0
M  V30 20 C 1.071707 2.887500 0.000000 0
M  V30 21 O 1.071707 3.712500 0.000000 0
M  V30 22 O 1.786178 2.475000 0.000000 0
M  V30 23 C -0.357235 0.412500 0.000000 0
M  V30 24 C 0.357236 0.000000 0.000000 0
M  V30 25 S 0.357236 -0.825000 0.000000 0
M  V30 26 N -1.071706 0.000000 0.000000 0
M  V30 27 C -1.071706 -0.825000 0.000000 0
M  V30 28 C -1.786177 -1.237500 0.000000 0
M  V30 29 C -1.786177 -2.062500 0.000000 0
M  V30 30 C -2.500648 -2.475000 0.000000 0
M  V30 31 N -3.215119 -2.062500 0.000000 0
M  V30 32 C -2.500648 -3.300000 0.000000 0
M  V30 33 O -3.215119 -3.712500 0.000000 0
M  V30 34 O -1.786177 -3.712500 0.000000 0
M  V30 35 O -0.357235 -1.237500 0.000000 0
M  V30 END ATOM
M  V30 BEGIN BOND
M  V30 1 1 1 2
M  V30 2 2 2 3
M  V30 3 1 3 4
M  V30 4 2 4 5
M  V30 5 1 5 6
M  V30 6 2 6 7
M  V30 7 1 7 8
M  V30 8 2 8 9
M  V30 9 1 9 10
M  V30 10 2 10 11
M  V30 11 1 6 11
M  V30 12 1 11 12
M  V30 13 2 12 13
M  V30 14 1 13 14
M  V30 15 2 1 14
M  V30 16 1 5 14
M  V30 17 1 1 15
M  V30 18 2 16 17
M  V30 19 1 17 18
M  V30 20 1 18 19
M  V30 21 1 19 20
M  V30 22 1 20 21
M  V30 23 2 20 22
M  V30 24 1 17 23
M  V30 25 1 23 24
M  V30 26 1 24 25
M  V30 27 1 23 26
M  V30 28 1 26 27
M  V30 29 1 27 28
M  V30 30 1 28 29
M  V30 31 1 29 30
M  V30 32 1 30 31
M  V30 33 1 30 32
M  V30 34 1 32 33
M  V30 35 2 32 34
M  V30 36 2 27 35
M  V30 37 1 4 25
M  V30 END BOND
M  V30 END CTAB
M  END
```

1-Methylphenanthrene glutathione II

```
1-Methylphenanthrene glutathione II A.mol
  ChemDraw04122413042D

  0  0  0     0  0              0 V3000
M  V30 BEGIN CTAB
M  V30 COUNTS 36 38 0 0 0
M  V30 BEGIN ATOM
M  V30 1 C 2.500648 -2.062500 0.000000 0
M  V30 2 C 2.500648 -1.237499 0.000000 0
M  V30 3 C 1.786178 -0.824999 0.000000 0
M  V30 4 C 1.071706 -1.237499 0.000000 0
M  V30 5 C 1.071706 -2.062499 0.000000 0
M  V30 6 C 0.357234 -2.475000 0.000000 0
M  V30 7 C -0.357235 -2.062499 0.000000 0
M  V30 8 C -1.071706 -2.475000 0.000000 0
M  V30 9 C -1.071706 -3.300000 0.000000 0
M  V30 10 C -0.357237 -3.712497 0.000000 0
M  V30 11 C 0.357234 -3.300000 0.000000 0
M  V30 12 C 1.071706 -3.712497 0.000000 0
M  V30 13 C 1.786177 -3.300000 0.000000 0
M  V30 14 C 1.786177 -2.475000 0.000000 0
M  V30 15 C 3.215119 -2.475000 0.000000 0
M  V30 16 O -2.500648 -0.824999 0.000000 0
M  V30 17 C -1.786177 -1.237499 0.000000 0
M  V30 18 N -1.786177 -2.062499 0.000000 0
M  V30 19 C -2.500648 -2.475000 0.000000 0
M  V30 20 C -2.500648 -3.300000 0.000000 0
M  V30 21 O -1.786177 -3.712500 0.000000 0
M  V30 22 O -3.215119 -3.712500 0.000000 0
M  V30 23 C -1.071706 -0.824999 0.000000 0
M  V30 24 C -0.357235 -1.237499 0.000000 0
M  V30 25 S 0.357234 -0.824999 0.000000 0
M  V30 26 N -1.071706 -0.000000 0.000000 0
M  V30 27 C -0.357235 0.412500 0.000000 0
M  V30 28 C -0.357235 1.237500 0.000000 0
M  V30 29 C 0.357234 1.650000 0.000000 0
M  V30 30 C 0.357234 2.475000 0.000000 0
M  V30 31 N -0.357235 2.887501 0.000000 0
M  V30 32 C 1.071706 2.887501 0.000000 0
M  V30 33 O 1.071706 3.712500 0.000000 0
M  V30 34 O 1.786178 2.475000 0.000000 0
M  V30 35 O 0.357234 -0.000000 0.000000 0
M  V30 36 O 1.786178 -0.000000 0.000000 0
M  V30 END ATOM
M  V30 BEGIN BOND
M  V30 1 2 1 2
M  V30 2 1 2 3
M  V30 3 1 3 4
M  V30 4 1 4 5
M  V30 5 1 5 6
M  V30 6 2 6 7
M  V30 7 1 7 8
M  V30 8 2 8 9
M  V30 9 1 9 10
M  V30 10 2 10 11
M  V30 11 1 6 11
M  V30 12 1 11 12
M  V30 13 2 12 13
M  V30 14 1 13 14
M  V30 15 1 1 14
M  V30 16 2 5 14
M  V30 17 1 1 15
M  V30 18 2 16 17
M  V30 19 1 17 18
M  V30 20 1 18 19
M  V30 21 1 19 20
M  V30 22 1 20 21
M  V30 23 2 20 22
M  V30 24 1 17 23
M  V30 25 1 23 24
M  V30 26 1 24 25
M  V30 27 1 23 26
M  V30 28 1 26 27
M  V30 29 1 27 28
M  V30 30 1 28 29
M  V30 31 1 29 30
M  V30 32 1 30 31
M  V30 33 1 30 32
M  V30 34 1 32 33
M  V30 35 2 32 34
M  V30 36 2 27 35
M  V30 37 1 4 25
M  V30 38 1 3 36
M  V30 END BOND
M  V30 END CTAB
M  END
```

Anthracene cysteinylglycine

```
Anthracene cysteinylglycine.mol.sb-cf5d4b74-z1841g
  ChemDraw04102410372D

  0  0  0     0  0              0 V3000
M  V30 BEGIN CTAB
M  V30 COUNTS 25 27 0 0 0
M  V30 BEGIN ATOM
M  V30 1 O 2.496481 0.411813 0.000000 0
M  V30 2 C 1.783200 0.823625 0.000000 0
M  V30 3 C 1.069920 0.411813 0.000000 0
M  V30 4 N 0.356640 0.823625 0.000000 0
M  V30 5 C 1.069920 -0.411813 0.000000 0
M  V30 6 S 0.356640 -0.823625 0.000000 0
M  V30 7 C 0.356640 -1.647250 0.000000 0
M  V30 8 C 1.069920 -2.059063 0.000000 0
M  V30 9 C 1.069920 -2.882687 0.000000 0
M  V30 10 C 0.356640 -3.294500 0.000000 0
M  V30 11 C -0.356640 -2.882687 0.000000 0
M  V30 12 C -1.069920 -3.294500 0.000000 0
M  V30 13 C -1.783200 -2.882687 0.000000 0
M  V30 14 C -2.496480 -3.294500 0.000000 0
M  V30 15 C -3.209761 -2.882687 0.000000 0
M  V30 16 C -3.209761 -2.059063 0.000000 0
M  V30 17 C -2.496480 -1.647250 0.000000 0
M  V30 18 C -1.783200 -2.059063 0.000000 0
M  V30 19 C -1.069920 -1.647250 0.000000 0
M  V30 20 C -0.356640 -2.059063 0.000000 0
M  V30 21 N 1.783200 1.647250 0.000000 0
M  V30 22 C 2.496481 2.059063 0.000000 0
M  V30 23 C 2.496481 2.882687 0.000000 0
M  V30 24 O 3.209761 3.294500 0.000000 0
M  V30 25 O 1.783200 3.294500 0.000000 0
M  V30 END ATOM
M  V30 BEGIN BOND
M  V30 1 2 1 2
M  V30 2 1 2 3
M  V30 3 1 3 4
M  V30 4 1 3 5
M  V30 5 1 5 6
M  V30 6 1 6 7
M  V30 7 2 7 8
M  V30 8 1 8 9
M  V30 9 2 9 10
M  V30 10 1 10 11
M  V30 11 2 11 12
M  V30 12 1 12 13
M  V30 13 2 13 14
M  V30 14 1 14 15
M  V30 15 2 15 16
M  V30 16 1 16 17
M  V30 17 2 17 18
M  V30 18 1 13 18
M  V30 19 1 18 19
M  V30 20 2 19 20
M  V30 21 1 7 20
M  V30 22 1 11 20
M  V30 23 1 2 21
M  V30 24 1 21 22
M  V30 25 1 22 23
M  V30 26 1 23 24
M  V30 27 2 23 25
M  V30 END BOND
M  V30 END CTAB
M  END
```

Benz(a)anthracene cysteinylglycine I A

```
Benz(a)anthracene cysteinylglycine I A.mol.sb-cf5d4b74-kG3xTe
  ChemDraw04102410102D

  0  0  0     0  0              0 V3000
M  V30 BEGIN CTAB
M  V30 COUNTS 29 32 0 0 0
M  V30 BEGIN ATOM
M  V30 1 C -1.786178 -0.206250 0.000000 0
M  V30 2 C -1.071706 -0.618750 0.000000 0
M  V30 3 C -1.071706 -1.443750 0.000000 0
M  V30 4 C -1.786178 -1.856250 0.000000 0
M  V30 5 C -2.500648 -1.443750 0.000000 0
M  V30 6 C -3.215119 -1.856250 0.000000 0
M  V30 7 C -3.929590 -1.443750 0.000000 0
M  V30 8 C -4.644061 -1.856250 0.000000 0
M  V30 9 C -5.358532 -1.443750 0.000000 0
M  V30 10 C -5.358532 -0.618750 0.000000 0
M  V30 11 C -4.644061 -0.206250 0.000000 0
M  V30 12 C -3.929590 -0.618750 0.000000 0
M  V30 13 C -3.215119 -0.206250 0.000000 0
M  V30 14 C -2.500648 -0.618750 0.000000 0
M  V30 15 C -0.357235 -0.206250 0.000000 0
M  V30 16 C -0.357235 0.618750 0.000000 0
M  V30 17 C -1.071706 1.031250 0.000000 0
M  V30 18 C -1.786178 0.618750 0.000000 0
M  V30 19 O 2.500648 -0.206250 0.000000 0
M  V30 20 C 2.500648 0.618750 0.000000 0
M  V30 21 N 3.215119 1.031250 0.000000 0
M  V30 22 C 3.929590 0.618750 0.000000 0
M  V30 23 C 4.644061 1.031250 0.000000 0
M  V30 24 O 5.358532 0.618750 0.000000 0
M  V30 25 O 4.644061 1.856250 0.000000 0
M  V30 26 C 1.786178 1.031250 0.000000 0
M  V30 27 C 1.071706 0.618750 0.000000 0
M  V30 28 S 0.357235 1.031250 0.000000 0
M  V30 29 N 1.786178 1.856250 0.000000 0
M  V30 END ATOM
M  V30 BEGIN BOND
M  V30 1 1 1 2
M  V30 2 1 2 3
M  V30 3 2 3 4
M  V30 4 1 4 5
M  V30 5 2 5 6
M  V30 6 1 6 7
M  V30 7 2 7 8
M  V30 8 1 8 9
M  V30 9 2 9 10
M  V30 10 1 10 11
M  V30 11 2 11 12
M  V30 12 1 7 12
M  V30 13 1 12 13
M  V30 14 2 13 14
M  V30 15 1 5 14
M  V30 16 1 1 14
M  V30 17 1 15 16
M  V30 18 2 16 17
M  V30 19 1 17 18
M  V30 20 2 18 1
M  V30 21 2 15 2
M  V30 22 2 19 20
M  V30 23 1 20 21
M  V30 24 1 21 22
M  V30 25 1 22 23
M  V30 26 1 23 24
M  V30 27 2 23 25
M  V30 28 1 20 26
M  V30 29 1 26 27
M  V30 30 1 27 28
M  V30 31 1 26 29
M  V30 32 1 28 16
M  V30 END BOND
M  V30 END CTAB
M  END
```

Benz(a)anthracene cysteinylglycine I B

```
Benz(a)anthracene cysteinylglycine I B.mol.sb-cf5d4b74-z8PVmF
  ChemDraw04102410112D

  0  0  0     0  0              0 V3000
M  V30 BEGIN CTAB
M  V30 COUNTS 29 32 0 0 0
M  V30 BEGIN ATOM
M  V30 1 C 3.929590 0.206250 0.000000 0
M  V30 2 C 4.644061 -0.206250 0.000000 0
M  V30 3 C 4.644061 -1.031250 0.000000 0
M  V30 4 C 3.929590 -1.443750 0.000000 0
M  V30 5 C 3.215119 -1.031250 0.000000 0
M  V30 6 C 2.500648 -1.443750 0.000000 0
M  V30 7 C 1.786177 -1.031250 0.000000 0
M  V30 8 C 1.071706 -1.443750 0.000000 0
M  V30 9 C 0.357235 -1.031250 0.000000 0
M  V30 10 C 0.357235 -0.206250 0.000000 0
M  V30 11 C 1.071706 0.206250 0.000000 0
M  V30 12 C 1.786177 -0.206250 0.000000 0
M  V30 13 C 2.500648 0.206250 0.000000 0
M  V30 14 C 3.215119 -0.206250 0.000000 0
M  V30 15 C 5.358532 0.206250 0.000000 0
M  V30 16 C 5.358532 1.031250 0.000000 0
M  V30 17 C 4.644061 1.443750 0.000000 0
M  V30 18 C 3.929590 1.031250 0.000000 0
M  V30 19 O -2.500648 -1.031250 0.000000 0
M  V30 20 C -2.500648 -0.206250 0.000000 0
M  V30 21 N -3.215119 0.206250 0.000000 0
M  V30 22 C -3.929590 -0.206250 0.000000 0
M  V30 23 C -4.644061 0.206250 0.000000 0
M  V30 24 O -5.358532 -0.206250 0.000000 0
M  V30 25 O -4.644061 1.031250 0.000000 0
M  V30 26 C -1.786178 0.206250 0.000000 0
M  V30 27 C -1.071707 -0.206250 0.000000 0
M  V30 28 S -0.357235 0.206250 0.000000 0
M  V30 29 N -1.786178 1.031250 0.000000 0
M  V30 END ATOM
M  V30 BEGIN BOND
M  V30 1 1 1 2
M  V30 2 1 2 3
M  V30 3 2 3 4
M  V30 4 1 4 5
M  V30 5 2 5 6
M  V30 6 1 6 7
M  V30 7 2 7 8
M  V30 8 1 8 9
M  V30 9 2 9 10
M  V30 10 1 10 11
M  V30 11 2 11 12
M  V30 12 1 7 12
M  V30 13 1 12 13
M  V30 14 2 13 14
M  V30 15 1 5 14
M  V30 16 1 1 14
M  V30 17 1 15 16
M  V30 18 2 16 17
M  V30 19 1 17 18
M  V30 20 2 18 1
M  V30 21 2 15 2
M  V30 22 2 19 20
M  V30 23 1 20 21
M  V30 24 1 21 22
M  V30 25 1 22 23
M  V30 26 1 23 24
M  V30 27 2 23 25
M  V30 28 1 20 26
M  V30 29 1 26 27
M  V30 30 1 27 28
M  V30 31 1 26 29
M  V30 32 1 28 10
M  V30 END BOND
M  V30 END CTAB
M  END
```

Benz(a)anthracene cysteinylglycine II

```
Benz(a)anthracene cysteinylglycine II A.mol.sb-cf5d4b74-354Kvh
  ChemDraw04102410122D

  0  0  0     0  0              0 V3000
M  V30 BEGIN CTAB
M  V30 COUNTS 30 33 0 0 0
M  V30 BEGIN ATOM
M  V30 1 C -1.783200 -0.205906 0.000000 0
M  V30 2 C -1.069920 -0.617719 0.000000 0
M  V30 3 C -1.069920 -1.441344 0.000000 0
M  V30 4 C -1.783200 -1.853156 0.000000 0
M  V30 5 C -2.496480 -1.441344 0.000000 0
M  V30 6 C -3.209761 -1.853156 0.000000 0
M  V30 7 C -3.923041 -1.441344 0.000000 0
M  V30 8 C -4.636321 -1.853156 0.000000 0
M  V30 9 C -5.349601 -1.441344 0.000000 0
M  V30 10 C -5.349601 -0.617719 0.000000 0
M  V30 11 C -4.636321 -0.205906 0.000000 0
M  V30 12 C -3.923041 -0.617719 0.000000 0
M  V30 13 C -3.209761 -0.205906 0.000000 0
M  V30 14 C -2.496480 -0.617719 0.000000 0
M  V30 15 C -0.356640 -0.205906 0.000000 0
M  V30 16 C -0.356640 0.617719 0.000000 0
M  V30 17 C -1.069920 1.029531 0.000000 0
M  V30 18 C -1.783200 0.617719 0.000000 0
M  V30 19 O 2.496481 -0.205906 0.000000 0
M  V30 20 C 2.496481 0.617719 0.000000 0
M  V30 21 N 3.209761 1.029531 0.000000 0
M  V30 22 C 3.923041 0.617719 0.000000 0
M  V30 23 C 4.636321 1.029531 0.000000 0
M  V30 24 O 5.349601 0.617719 0.000000 0
M  V30 25 O 4.636321 1.853156 0.000000 0
M  V30 26 C 1.783200 1.029531 0.000000 0
M  V30 27 C 1.069920 0.617719 0.000000 0
M  V30 28 S 0.356640 1.029531 0.000000 0
M  V30 29 N 1.783200 1.853156 0.000000 0
M  V30 30 O 0.356640 -0.617719 0.000000 0
M  V30 END ATOM
M  V30 BEGIN BOND
M  V30 1 2 1 2
M  V30 2 1 2 3
M  V30 3 2 3 4
M  V30 4 1 4 5
M  V30 5 2 5 6
M  V30 6 1 6 7
M  V30 7 2 7 8
M  V30 8 1 8 9
M  V30 9 2 9 10
M  V30 10 1 10 11
M  V30 11 2 11 12
M  V30 12 1 7 12
M  V30 13 1 12 13
M  V30 14 2 13 14
M  V30 15 1 5 14
M  V30 16 1 1 14
M  V30 17 1 15 16
M  V30 18 1 16 17
M  V30 19 2 17 18
M  V30 20 1 18 1
M  V30 21 1 15 2
M  V30 22 2 19 20
M  V30 23 1 20 21
M  V30 24 1 21 22
M  V30 25 1 22 23
M  V30 26 1 23 24
M  V30 27 2 23 25
M  V30 28 1 20 26
M  V30 29 1 26 27
M  V30 30 1 27 28
M  V30 31 1 26 29
M  V30 32 1 15 30
M  V30 33 1 28 16
M  V30 END BOND
M  V30 END CTAB
M  END
```

Benz(a)anthracene cysteinylglycine III

```
Benz(a)anthracene cysteinylglycine III A.mol.sb-cf5d4b74-FMglso
  ChemDraw04102410192D

  0  0  0     0  0              0 V3000
M  V30 BEGIN CTAB
M  V30 COUNTS 31 34 0 0 0
M  V30 BEGIN ATOM
M  V30 1 C -1.428942 -0.206250 0.000000 0
M  V30 2 C -0.714471 -0.618750 0.000000 0
M  V30 3 C -0.714471 -1.443750 0.000000 0
M  V30 4 C -1.428942 -1.856250 0.000000 0
M  V30 5 C -2.143413 -1.443750 0.000000 0
M  V30 6 C -2.857884 -1.856250 0.000000 0
M  V30 7 C -3.572355 -1.443750 0.000000 0
M  V30 8 C -4.286826 -1.856250 0.000000 0
M  V30 9 C -5.001297 -1.443750 0.000000 0
M  V30 10 C -5.001297 -0.618750 0.000000 0
M  V30 11 C -4.286826 -0.206250 0.000000 0
M  V30 12 C -3.572355 -0.618750 0.000000 0
M  V30 13 C -2.857884 -0.206250 0.000000 0
M  V30 14 C -2.143413 -0.618750 0.000000 0
M  V30 15 C 0.000000 -0.206250 0.000000 0
M  V30 16 C 0.000000 0.618750 0.000000 0
M  V30 17 C -0.714471 1.031250 0.000000 0
M  V30 18 C -1.428942 0.618750 0.000000 0
M  V30 19 O 2.857884 -0.206250 0.000000 0
M  V30 20 C 2.857884 0.618750 0.000000 0
M  V30 21 N 3.572355 1.031250 0.000000 0
M  V30 22 C 4.286826 0.618750 0.000000 0
M  V30 23 C 5.001297 1.031250 0.000000 0
M  V30 24 O 5.715768 0.618750 0.000000 0
M  V30 25 O 5.001297 1.856250 0.000000 0
M  V30 26 C 2.143413 1.031250 0.000000 0
M  V30 27 C 1.428942 0.618750 0.000000 0
M  V30 28 S 0.714471 1.031250 0.000000 0
M  V30 29 N 2.143413 1.856250 0.000000 0
M  V30 30 O -4.286826 0.618750 0.000000 0
M  V30 31 O -5.715768 -0.206250 0.000000 0
M  V30 END ATOM
M  V30 BEGIN BOND
M  V30 1 2 1 2
M  V30 2 1 2 3
M  V30 3 2 3 4
M  V30 4 1 4 5
M  V30 5 2 5 6
M  V30 6 1 6 7
M  V30 7 1 7 8
M  V30 8 2 8 9
M  V30 9 1 9 10
M  V30 10 1 10 11
M  V30 11 1 11 12
M  V30 12 2 7 12
M  V30 13 1 12 13
M  V30 14 2 13 14
M  V30 15 1 5 14
M  V30 16 1 1 14
M  V30 17 2 15 16
M  V30 18 1 16 17
M  V30 19 2 17 18
M  V30 20 1 18 1
M  V30 21 1 15 2
M  V30 22 2 19 20
M  V30 23 1 20 21
M  V30 24 1 21 22
M  V30 25 1 22 23
M  V30 26 1 23 24
M  V30 27 2 23 25
M  V30 28 1 20 26
M  V30 29 1 26 27
M  V30 30 1 27 28
M  V30 31 1 26 29
M  V30 32 1 28 16
M  V30 33 1 11 30
M  V30 34 1 10 31
M  V30 END BOND
M  V30 END CTAB
M  END
```

Benz(a)anthracene cysteinylglycine IV A

```
Benz(a)anthracene cysteinylglycine IV A.mol.sb-cf5d4b74-GS6kFf
  ChemDraw04102410222D

  0  0  0     0  0              0 V3000
M  V30 BEGIN CTAB
M  V30 COUNTS 32 35 0 0 0
M  V30 BEGIN ATOM
M  V30 1 C -1.428942 -0.206250 0.000000 0
M  V30 2 C -0.714471 -0.618750 0.000000 0
M  V30 3 C -0.714471 -1.443750 0.000000 0
M  V30 4 C -1.428942 -1.856250 0.000000 0
M  V30 5 C -2.143413 -1.443750 0.000000 0
M  V30 6 C -2.857884 -1.856250 0.000000 0
M  V30 7 C -3.572355 -1.443750 0.000000 0
M  V30 8 C -4.286826 -1.856250 0.000000 0
M  V30 9 C -5.001297 -1.443750 0.000000 0
M  V30 10 C -5.001297 -0.618750 0.000000 0
M  V30 11 C -4.286826 -0.206250 0.000000 0
M  V30 12 C -3.572355 -0.618750 0.000000 0
M  V30 13 C -2.857884 -0.206250 0.000000 0
M  V30 14 C -2.143413 -0.618750 0.000000 0
M  V30 15 C 0.000000 -0.206250 0.000000 0
M  V30 16 C 0.000000 0.618750 0.000000 0
M  V30 17 C -0.714471 1.031250 0.000000 0
M  V30 18 C -1.428942 0.618750 0.000000 0
M  V30 19 O 2.857884 -0.206250 0.000000 0
M  V30 20 C 2.857884 0.618750 0.000000 0
M  V30 21 N 3.572355 1.031250 0.000000 0
M  V30 22 C 4.286826 0.618750 0.000000 0
M  V30 23 C 5.001297 1.031250 0.000000 0
M  V30 24 O 5.715768 0.618750 0.000000 0
M  V30 25 O 5.001297 1.856250 0.000000 0
M  V30 26 C 2.143413 1.031250 0.000000 0
M  V30 27 C 1.428942 0.618750 0.000000 0
M  V30 28 S 0.714471 1.031250 0.000000 0
M  V30 29 N 2.143413 1.856250 0.000000 0
M  V30 30 O 0.714471 -0.618750 0.000000 0
M  V30 31 O -4.286826 0.618750 0.000000 0
M  V30 32 O -5.715768 -0.206250 0.000000 0
M  V30 END ATOM
M  V30 BEGIN BOND
M  V30 1 2 1 2
M  V30 2 1 2 3
M  V30 3 2 3 4
M  V30 4 1 4 5
M  V30 5 1 5 6
M  V30 6 2 6 7
M  V30 7 1 7 8
M  V30 8 2 8 9
M  V30 9 1 9 10
M  V30 10 1 10 11
M  V30 11 1 11 12
M  V30 12 1 7 12
M  V30 13 2 12 13
M  V30 14 1 13 14
M  V30 15 2 5 14
M  V30 16 1 1 14
M  V30 17 1 15 16
M  V30 18 1 16 17
M  V30 19 2 17 18
M  V30 20 1 18 1
M  V30 21 1 15 2
M  V30 22 2 19 20
M  V30 23 1 20 21
M  V30 24 1 21 22
M  V30 25 1 22 23
M  V30 26 1 23 24
M  V30 27 2 23 25
M  V30 28 1 20 26
M  V30 29 1 26 27
M  V30 30 1 27 28
M  V30 31 1 26 29
M  V30 32 1 28 16
M  V30 33 1 15 30
M  V30 34 1 11 31
M  V30 35 1 10 32
M  V30 END BOND
M  V30 END CTAB
M  END
```

Benz(a)anthracene cysteinylglycine IV B

```
Benz(a)anthracene cysteinylglycine IV B.mol.sb-cf5d4b74-SzCHmO
  ChemDraw04102410232D

  0  0  0     0  0              0 V3000
M  V30 BEGIN CTAB
M  V30 COUNTS 32 35 0 0 0
M  V30 BEGIN ATOM
M  V30 1 C 3.572355 0.206250 0.000000 0
M  V30 2 C 4.286826 -0.206250 0.000000 0
M  V30 3 C 4.286826 -1.031250 0.000000 0
M  V30 4 C 3.572355 -1.443750 0.000000 0
M  V30 5 C 2.857884 -1.031250 0.000000 0
M  V30 6 C 2.143413 -1.443750 0.000000 0
M  V30 7 C 1.428942 -1.031250 0.000000 0
M  V30 8 C 0.714471 -1.443750 0.000000 0
M  V30 9 C -0.000000 -1.031250 0.000000 0
M  V30 10 C -0.000000 -0.206250 0.000000 0
M  V30 11 C 0.714471 0.206250 0.000000 0
M  V30 12 C 1.428942 -0.206250 0.000000 0
M  V30 13 C 2.143413 0.206250 0.000000 0
M  V30 14 C 2.857884 -0.206250 0.000000 0
M  V30 15 C 5.001297 0.206250 0.000000 0
M  V30 16 C 5.001297 1.031250 0.000000 0
M  V30 17 C 4.286826 1.443750 0.000000 0
M  V30 18 C 3.572355 1.031250 0.000000 0
M  V30 19 O -2.857884 -1.031250 0.000000 0
M  V30 20 C -2.857884 -0.206250 0.000000 0
M  V30 21 N -3.572355 0.206250 0.000000 0
M  V30 22 C -4.286826 -0.206250 0.000000 0
M  V30 23 C -5.001297 0.206250 0.000000 0
M  V30 24 O -5.715768 -0.206250 0.000000 0
M  V30 25 O -5.001297 1.031250 0.000000 0
M  V30 26 C -2.143413 0.206250 0.000000 0
M  V30 27 C -1.428942 -0.206250 0.000000 0
M  V30 28 S -0.714471 0.206250 0.000000 0
M  V30 29 N -2.143413 1.031250 0.000000 0
M  V30 30 O 5.715768 -0.206250 0.000000 0
M  V30 31 O 0.714471 1.031250 0.000000 0
M  V30 32 O 5.715768 1.443750 0.000000 0
M  V30 END ATOM
M  V30 BEGIN BOND
M  V30 1 2 1 2
M  V30 2 1 2 3
M  V30 3 2 3 4
M  V30 4 1 4 5
M  V30 5 1 5 6
M  V30 6 2 6 7
M  V30 7 1 7 8
M  V30 8 2 8 9
M  V30 9 1 9 10
M  V30 10 1 10 11
M  V30 11 1 11 12
M  V30 12 1 7 12
M  V30 13 2 12 13
M  V30 14 1 13 14
M  V30 15 2 5 14
M  V30 16 1 1 14
M  V30 17 1 15 16
M  V30 18 1 16 17
M  V30 19 2 17 18
M  V30 20 1 18 1
M  V30 21 1 15 2
M  V30 22 2 19 20
M  V30 23 1 20 21
M  V30 24 1 21 22
M  V30 25 1 22 23
M  V30 26 1 23 24
M  V30 27 2 23 25
M  V30 28 1 20 26
M  V30 29 1 26 27
M  V30 30 1 27 28
M  V30 31 1 26 29
M  V30 32 1 15 30
M  V30 33 1 11 31
M  V30 34 1 28 10
M  V30 35 1 16 32
M  V30 END BOND
M  V30 END CTAB
M  END
```

Benz(a)anthracene glutathione I A

```
Benz(a)anthracene glutathione I A.mol.sb-cf5d4b74-Vubpdj
  ChemDraw04102410292D

  0  0  0     0  0              0 V3000
M  V30 BEGIN CTAB
M  V30 COUNTS 39 42 0 0 0
M  V30 BEGIN ATOM
M  V30 1 C -1.428942 -0.825000 0.000000 0
M  V30 2 C -0.714471 -1.237500 0.000000 0
M  V30 3 C -0.714471 -2.062500 0.000000 0
M  V30 4 C -1.428942 -2.475000 0.000000 0
M  V30 5 C -2.143413 -2.062500 0.000000 0
M  V30 6 C -2.857884 -2.475000 0.000000 0
M  V30 7 C -3.572355 -2.062500 0.000000 0
M  V30 8 C -4.286826 -2.475000 0.000000 0
M  V30 9 C -5.001297 -2.062500 0.000000 0
M  V30 10 C -5.001297 -1.237500 0.000000 0
M  V30 11 C -4.286826 -0.825000 0.000000 0
M  V30 12 C -3.572355 -1.237500 0.000000 0
M  V30 13 C -2.857884 -0.825000 0.000000 0
M  V30 14 C -2.143413 -1.237500 0.000000 0
M  V30 15 C 0.000000 -0.825000 0.000000 0
M  V30 16 C 0.000000 0.000000 0.000000 0
M  V30 17 C -0.714471 0.412500 0.000000 0
M  V30 18 C -1.428942 0.000000 0.000000 0
M  V30 19 O 2.857884 1.650000 0.000000 0
M  V30 20 C 2.143413 1.237500 0.000000 0
M  V30 21 N 1.428942 1.650000 0.000000 0
M  V30 22 C 1.428942 2.475000 0.000000 0
M  V30 23 C 0.714471 2.887500 0.000000 0
M  V30 24 O 0.000000 2.475000 0.000000 0
M  V30 25 O 0.714471 3.712500 0.000000 0
M  V30 26 C 2.143413 0.412500 0.000000 0
M  V30 27 C 1.428942 0.000000 0.000000 0
M  V30 28 S 0.714471 0.412500 0.000000 0
M  V30 29 N 2.857884 0.000000 0.000000 0
M  V30 30 C 2.857884 -0.825000 0.000000 0
M  V30 31 C 3.572355 -1.237500 0.000000 0
M  V30 32 C 3.572355 -2.062500 0.000000 0
M  V30 33 C 4.286826 -2.475000 0.000000 0
M  V30 34 N 5.001297 -2.062500 0.000000 0
M  V30 35 C 4.286826 -3.300000 0.000000 0
M  V30 36 O 5.001297 -3.712500 0.000000 0
M  V30 37 O 3.572355 -3.712500 0.000000 0
M  V30 38 O 2.143413 -1.237500 0.000000 0
M  V30 39 O 0.714471 -1.237500 0.000000 0
M  V30 END ATOM
M  V30 BEGIN BOND
M  V30 1 2 1 2
M  V30 2 1 2 3
M  V30 3 2 3 4
M  V30 4 1 4 5
M  V30 5 2 5 6
M  V30 6 1 6 7
M  V30 7 2 7 8
M  V30 8 1 8 9
M  V30 9 2 9 10
M  V30 10 1 10 11
M  V30 11 2 11 12
M  V30 12 1 7 12
M  V30 13 1 12 13
M  V30 14 2 13 14
M  V30 15 1 5 14
M  V30 16 1 1 14
M  V30 17 1 15 16
M  V30 18 1 16 17
M  V30 19 2 17 18
M  V30 20 1 18 1
M  V30 21 1 15 2
M  V30 22 2 19 20
M  V30 23 1 20 21
M  V30 24 1 21 22
M  V30 25 1 22 23
M  V30 26 1 23 24
M  V30 27 2 23 25
M  V30 28 1 20 26
M  V30 29 1 26 27
M  V30 30 1 27 28
M  V30 31 1 26 29
M  V30 32 1 29 30
M  V30 33 1 30 31
M  V30 34 1 31 32
M  V30 35 1 32 33
M  V30 36 1 33 34
M  V30 37 1 33 35
M  V30 38 1 35 36
M  V30 39 2 35 37
M  V30 40 2 30 38
M  V30 41 1 15 39
M  V30 42 1 16 28
M  V30 END BOND
M  V30 END CTAB
M  END
```

Benz(a)anthracene glutathione I B

```
Benz(a)anthracene glutathione I B.mol.sb-cf5d4b74-bREDHh
  ChemDraw04102410312D

  0  0  0     0  0              0 V3000
M  V30 BEGIN CTAB
M  V30 COUNTS 39 42 0 0 0
M  V30 BEGIN ATOM
M  V30 1 C 3.572353 0.412500 0.000000 0
M  V30 2 C 4.286825 0.000000 0.000000 0
M  V30 3 C 4.286825 -0.825000 0.000000 0
M  V30 4 C 3.572353 -1.237500 0.000000 0
M  V30 5 C 2.857884 -0.825000 0.000000 0
M  V30 6 C 2.143413 -1.237500 0.000000 0
M  V30 7 C 1.428941 -0.825000 0.000000 0
M  V30 8 C 0.714471 -1.237500 0.000000 0
M  V30 9 C 0.000000 -0.825000 0.000000 0
M  V30 10 C 0.000000 0.000000 0.000000 0
M  V30 11 C 0.714471 0.412500 0.000000 0
M  V30 12 C 1.428941 0.000000 0.000000 0
M  V30 13 C 2.143413 0.412500 0.000000 0
M  V30 14 C 2.857884 0.000000 0.000000 0
M  V30 15 C 5.001296 0.412500 0.000000 0
M  V30 16 C 5.001296 1.237500 0.000000 0
M  V30 17 C 4.286825 1.650000 0.000000 0
M  V30 18 C 3.572353 1.237500 0.000000 0
M  V30 19 O -2.857883 1.650000 0.000000 0
M  V30 20 C -2.143412 1.237500 0.000000 0
M  V30 21 N -1.428941 1.650000 0.000000 0
M  V30 22 C -1.428941 2.475000 0.000000 0
M  V30 23 C -0.714470 2.887500 0.000000 0
M  V30 24 O 0.000000 2.475000 0.000000 0
M  V30 25 O -0.714470 3.712500 0.000000 0
M  V30 26 C -2.143412 0.412500 0.000000 0
M  V30 27 C -1.428941 0.000000 0.000000 0
M  V30 28 S -0.714470 0.412500 0.000000 0
M  V30 29 N -2.857883 0.000000 0.000000 0
M  V30 30 C -2.857883 -0.825000 0.000000 0
M  V30 31 C -3.572354 -1.237500 0.000000 0
M  V30 32 C -3.572354 -2.062500 0.000000 0
M  V30 33 C -4.286825 -2.475000 0.000000 0
M  V30 34 N -5.001296 -2.062500 0.000000 0
M  V30 35 C -4.286825 -3.300000 0.000000 0
M  V30 36 O -5.001296 -3.712500 0.000000 0
M  V30 37 O -3.572354 -3.712500 0.000000 0
M  V30 38 O -2.143412 -1.237500 0.000000 0
M  V30 39 O 0.714471 1.237500 0.000000 0
M  V30 END ATOM
M  V30 BEGIN BOND
M  V30 1 2 1 2
M  V30 2 1 2 3
M  V30 3 2 3 4
M  V30 4 1 4 5
M  V30 5 2 5 6
M  V30 6 1 6 7
M  V30 7 1 7 8
M  V30 8 2 8 9
M  V30 9 1 9 10
M  V30 10 1 10 11
M  V30 11 1 11 12
M  V30 12 2 7 12
M  V30 13 1 12 13
M  V30 14 2 13 14
M  V30 15 1 5 14
M  V30 16 1 1 14
M  V30 17 2 15 16
M  V30 18 1 16 17
M  V30 19 2 17 18
M  V30 20 1 18 1
M  V30 21 1 15 2
M  V30 22 2 19 20
M  V30 23 1 20 21
M  V30 24 1 21 22
M  V30 25 1 22 23
M  V30 26 1 23 24
M  V30 27 2 23 25
M  V30 28 1 20 26
M  V30 29 1 26 27
M  V30 30 1 27 28
M  V30 31 1 26 29
M  V30 32 1 29 30
M  V30 33 1 30 31
M  V30 34 1 31 32
M  V30 35 1 32 33
M  V30 36 1 33 34
M  V30 37 1 33 35
M  V30 38 1 35 36
M  V30 39 2 35 37
M  V30 40 2 30 38
M  V30 41 1 10 28
M  V30 42 1 11 39
M  V30 END BOND
M  V30 END CTAB
M  END
```

Benz(a)anthracene glutathione II

```
Benz(a)anthracene glutathione II A.mol.sb-cf5d4b74-y6nuw5
  ChemDraw04102410322D

  0  0  0     0  0              0 V3000
M  V30 BEGIN CTAB
M  V30 COUNTS 40 43 0 0 0
M  V30 BEGIN ATOM
M  V30 1 C -1.428942 -0.824999 0.000000 0
M  V30 2 C -0.714471 -1.237499 0.000000 0
M  V30 3 C -0.714471 -2.062499 0.000000 0
M  V30 4 C -1.428942 -2.475000 0.000000 0
M  V30 5 C -2.143413 -2.062499 0.000000 0
M  V30 6 C -2.857884 -2.475000 0.000000 0
M  V30 7 C -3.572355 -2.062499 0.000000 0
M  V30 8 C -4.286825 -2.475000 0.000000 0
M  V30 9 C -5.001297 -2.062499 0.000000 0
M  V30 10 C -5.001297 -1.237499 0.000000 0
M  V30 11 C -4.286825 -0.824999 0.000000 0
M  V30 12 C -3.572355 -1.237499 0.000000 0
M  V30 13 C -2.857884 -0.824999 0.000000 0
M  V30 14 C -2.143413 -1.237499 0.000000 0
M  V30 15 C 0.000000 -0.824999 0.000000 0
M  V30 16 C 0.000000 -0.000000 0.000000 0
M  V30 17 C -0.714471 0.412500 0.000000 0
M  V30 18 C -1.428942 -0.000000 0.000000 0
M  V30 19 O 2.857884 1.650000 0.000000 0
M  V30 20 C 2.143413 1.237500 0.000000 0
M  V30 21 N 1.428942 1.650000 0.000000 0
M  V30 22 C 1.428942 2.475000 0.000000 0
M  V30 23 C 0.714471 2.887501 0.000000 0
M  V30 24 O 0.714471 3.712500 0.000000 0
M  V30 25 O 0.000000 2.475000 0.000000 0
M  V30 26 C 2.143413 0.412500 0.000000 0
M  V30 27 C 1.428942 -0.000000 0.000000 0
M  V30 28 S 0.714471 0.412500 0.000000 0
M  V30 29 N 2.857884 -0.000000 0.000000 0
M  V30 30 C 2.857884 -0.824999 0.000000 0
M  V30 31 C 3.572355 -1.237499 0.000000 0
M  V30 32 C 3.572355 -2.062499 0.000000 0
M  V30 33 C 4.286825 -2.475000 0.000000 0
M  V30 34 N 5.001297 -2.062499 0.000000 0
M  V30 35 C 4.286825 -3.300000 0.000000 0
M  V30 36 O 5.001297 -3.712500 0.000000 0
M  V30 37 O 3.572355 -3.712500 0.000000 0
M  V30 38 O 2.143413 -1.237499 0.000000 0
M  V30 39 O 0.714471 -1.237499 0.000000 0
M  V30 40 O -4.286825 0.000001 0.000000 0
M  V30 END ATOM
M  V30 BEGIN BOND
M  V30 1 2 1 2
M  V30 2 1 2 3
M  V30 3 2 3 4
M  V30 4 1 4 5
M  V30 5 2 5 6
M  V30 6 1 6 7
M  V30 7 2 7 8
M  V30 8 1 8 9
M  V30 9 2 9 10
M  V30 10 1 10 11
M  V30 11 2 11 12
M  V30 12 1 7 12
M  V30 13 1 12 13
M  V30 14 2 13 14
M  V30 15 1 5 14
M  V30 16 1 1 14
M  V30 17 1 15 16
M  V30 18 1 16 17
M  V30 19 2 17 18
M  V30 20 1 18 1
M  V30 21 1 15 2
M  V30 22 2 19 20
M  V30 23 1 20 21
M  V30 24 1 21 22
M  V30 25 1 22 23
M  V30 26 1 23 24
M  V30 27 2 23 25
M  V30 28 1 20 26
M  V30 29 1 26 27
M  V30 30 1 27 28
M  V30 31 1 26 29
M  V30 32 1 29 30
M  V30 33 1 30 31
M  V30 34 1 31 32
M  V30 35 1 32 33
M  V30 36 1 33 34
M  V30 37 1 33 35
M  V30 38 1 35 36
M  V30 39 2 35 37
M  V30 40 2 30 38
M  V30 41 1 28 16
M  V30 42 1 15 39
M  V30 43 1 11 40
M  V30 END BOND
M  V30 END CTAB
M  END
```

Benzo(a)pyrene cysteinylglycine A

```
Benzo(a)pyrene cysteinylglycine.mol.sb-94fd87bf-0a3CDh
  ChemDraw08232321292D

  0  0  0     0  0              0 V3000
M  V30 BEGIN CTAB
M  V30 COUNTS 34 38 0 0 0
M  V30 BEGIN ATOM
M  V30 1 C -3.566401 1.647250 0.000000 0
M  V30 2 N -2.139841 1.647250 0.000000 0
M  V30 3 C -2.853121 2.059062 0.000000 0
M  V30 4 O -3.566401 0.823625 0.000000 0
M  V30 5 O -4.279681 2.059062 0.000000 0
M  V30 6 C -1.426561 2.059062 0.000000 0
M  V30 7 C -0.713280 1.647250 0.000000 0
M  V30 8 C -0.713280 0.823625 0.000000 0
M  V30 9 O -1.426561 2.882688 0.000000 0
M  V30 10 N -0.000000 2.059062 0.000000 0
M  V30 11 C 0.713280 -0.823625 0.000000 0
M  V30 12 C 0.713280 -1.647250 0.000000 0
M  V30 13 C 1.426560 -2.059063 0.000000 0
M  V30 14 C 2.139841 -1.647250 0.000000 0
M  V30 15 C 2.853120 -2.059063 0.000000 0
M  V30 16 C 3.566401 -1.647250 0.000000 0
M  V30 17 C 3.566401 -0.823625 0.000000 0
M  V30 18 C 4.279681 -0.411812 0.000000 0
M  V30 19 C 4.279681 0.411812 0.000000 0
M  V30 20 C 3.566401 0.823625 0.000000 0
M  V30 21 C 2.853121 0.411812 0.000000 0
M  V30 22 C 2.139841 0.823625 0.000000 0
M  V30 23 C 1.426560 0.411812 0.000000 0
M  V30 24 C 1.426560 -0.411812 0.000000 0
M  V30 25 C 2.139841 -0.823625 0.000000 0
M  V30 26 C 2.853121 -0.411812 0.000000 0
M  V30 27 C -0.000000 -2.059063 0.000000 0
M  V30 28 C -0.713280 -1.647250 0.000000 0
M  V30 29 C -0.713280 -0.823625 0.000000 0
M  V30 30 C -0.000000 -0.411812 0.000000 0
M  V30 31 O -1.426561 -0.411812 0.000000 0
M  V30 32 O -1.426561 -2.059063 0.000000 0
M  V30 33 S -0.000000 0.411812 0.000000 0
M  V30 34 O -0.000000 -2.882688 0.000000 0
M  V30 END ATOM
M  V30 BEGIN BOND
M  V30 1 1 2 3
M  V30 2 1 1 3
M  V30 3 2 1 4
M  V30 4 1 1 5
M  V30 5 1 2 6
M  V30 6 1 6 7
M  V30 7 1 7 8
M  V30 8 2 6 9
M  V30 9 1 7 10
M  V30 10 2 11 12
M  V30 11 1 12 13
M  V30 12 2 13 14
M  V30 13 1 14 15
M  V30 14 2 15 16
M  V30 15 1 16 17
M  V30 16 2 17 18
M  V30 17 1 18 19
M  V30 18 2 19 20
M  V30 19 1 20 21
M  V30 20 2 21 22
M  V30 21 1 22 23
M  V30 22 2 23 24
M  V30 23 1 11 24
M  V30 24 1 24 25
M  V30 25 1 14 25
M  V30 26 2 25 26
M  V30 27 1 17 26
M  V30 28 1 21 26
M  V30 29 1 27 28
M  V30 30 1 28 29
M  V30 31 1 29 30
M  V30 32 1 30 11
M  V30 33 1 27 12
M  V30 34 1 29 31
M  V30 35 1 28 32
M  V30 36 1 33 8
M  V30 37 1 30 33
M  V30 38 1 27 34
M  V30 END BOND
M  V30 END CTAB
M  END
```

Benzo(a)pyrene cysteinylglycine B

```
Benzo(a)pyrene cysteinylglycine B.mol.sb-e18dad7b-Qxc8Cp
  ChemDraw03172417562D

  0  0  0     0  0              0 V3000
M  V30 BEGIN CTAB
M  V30 COUNTS 34 38 0 0 0
M  V30 BEGIN ATOM
M  V30 1 C -3.929592 1.237501 0.000000 0
M  V30 2 N -2.500650 1.237501 0.000000 0
M  V30 3 C -3.215120 1.650000 0.000000 0
M  V30 4 O -3.929592 0.412500 0.000000 0
M  V30 5 O -4.644063 1.650000 0.000000 0
M  V30 6 C -1.786179 1.650000 0.000000 0
M  V30 7 C -1.071707 1.237501 0.000000 0
M  V30 8 C -1.071707 0.412500 0.000000 0
M  V30 9 O -1.786179 2.475001 0.000000 0
M  V30 10 N -0.357236 1.650000 0.000000 0
M  V30 11 C 0.357236 -1.237501 0.000000 0
M  V30 12 C 0.357236 -2.062500 0.000000 0
M  V30 13 C 1.071706 -2.475001 0.000000 0
M  V30 14 C 1.786178 -2.062500 0.000000 0
M  V30 15 C 2.500648 -2.475001 0.000000 0
M  V30 16 C 3.215120 -2.062500 0.000000 0
M  V30 17 C 3.215120 -1.237501 0.000000 0
M  V30 18 C 3.929592 -0.825000 0.000000 0
M  V30 19 C 3.929592 -0.000000 0.000000 0
M  V30 20 C 3.215120 0.412500 0.000000 0
M  V30 21 C 2.500649 -0.000000 0.000000 0
M  V30 22 C 1.786178 0.412500 0.000000 0
M  V30 23 C 1.071706 -0.000000 0.000000 0
M  V30 24 C 1.071706 -0.825000 0.000000 0
M  V30 25 C 1.786178 -1.237501 0.000000 0
M  V30 26 C 2.500649 -0.825000 0.000000 0
M  V30 27 C -0.357236 -2.475001 0.000000 0
M  V30 28 C -1.071707 -2.062500 0.000000 0
M  V30 29 C -1.071707 -1.237501 0.000000 0
M  V30 30 C -0.357236 -0.825000 0.000000 0
M  V30 31 S -0.357236 -0.000000 0.000000 0
M  V30 32 O 4.644063 0.412500 0.000000 0
M  V30 33 O -1.786177 -0.825001 0.000000 0
M  V30 34 O 3.215120 1.237500 0.000000 0
M  V30 END ATOM
M  V30 BEGIN BOND
M  V30 1 1 2 3
M  V30 2 1 1 3
M  V30 3 2 1 4
M  V30 4 1 1 5
M  V30 5 1 2 6
M  V30 6 1 6 7
M  V30 7 1 7 8
M  V30 8 2 6 9
M  V30 9 1 7 10
M  V30 10 2 11 12
M  V30 11 1 12 13
M  V30 12 2 13 14
M  V30 13 1 14 15
M  V30 14 2 15 16
M  V30 15 1 16 17
M  V30 16 2 17 18
M  V30 17 1 18 19
M  V30 18 1 19 20
M  V30 19 1 20 21
M  V30 20 2 21 22
M  V30 21 1 22 23
M  V30 22 2 23 24
M  V30 23 1 11 24
M  V30 24 1 24 25
M  V30 25 1 14 25
M  V30 26 2 25 26
M  V30 27 1 17 26
M  V30 28 1 21 26
M  V30 29 2 27 28
M  V30 30 1 28 29
M  V30 31 1 29 30
M  V30 32 1 30 11
M  V30 33 1 27 12
M  V30 34 1 31 8
M  V30 35 1 30 31
M  V30 36 1 19 32
M  V30 37 1 29 33
M  V30 38 1 20 34
M  V30 END BOND
M  V30 END CTAB
M  END
```

Chrysene cysteine

```
Chrysene cysteine II.mol
  ChemDraw04222408202D

  0  0  0     0  0              0 V3000
M  V30 BEGIN CTAB
M  V30 COUNTS 28 31 0 0 0
M  V30 BEGIN ATOM
M  V30 1 C 0.357235 1.031250 0.000000 0
M  V30 2 C 1.071706 0.618750 0.000000 0
M  V30 3 C 1.071706 -0.206250 0.000000 0
M  V30 4 C 0.357235 -0.618750 0.000000 0
M  V30 5 C -0.357235 -0.206250 0.000000 0
M  V30 6 C -1.071706 -0.618750 0.000000 0
M  V30 7 C -1.071706 -1.443750 0.000000 0
M  V30 8 C -1.786177 -1.856250 0.000000 0
M  V30 9 C -2.500649 -1.443750 0.000000 0
M  V30 10 C -3.215118 -1.856250 0.000000 0
M  V30 11 C -3.929591 -1.443750 0.000000 0
M  V30 12 C -3.929591 -0.618750 0.000000 0
M  V30 13 C -3.215118 -0.206250 0.000000 0
M  V30 14 C -2.500649 -0.618750 0.000000 0
M  V30 15 C -1.786177 -0.206250 0.000000 0
M  V30 16 C -1.786177 0.618750 0.000000 0
M  V30 17 C -1.071706 1.031250 0.000000 0
M  V30 18 C -0.357235 0.618750 0.000000 0
M  V30 19 C 3.929590 0.618750 0.000000 0
M  V30 20 N 3.215118 1.856249 0.000000 0
M  V30 21 C 3.215118 1.031250 0.000000 0
M  V30 22 C 2.500648 0.618750 0.000000 0
M  V30 23 S 1.786177 1.031250 0.000000 0
M  V30 24 O 3.929590 -0.206250 0.000000 0
M  V30 25 O 4.644060 1.031250 0.000000 0
M  V30 26 O -3.215118 0.618750 0.000000 0
M  V30 27 O -4.644060 -0.206250 0.000000 0
M  V30 28 O 0.357236 1.856250 0.000000 0
M  V30 END ATOM
M  V30 BEGIN BOND
M  V30 1 1 1 2
M  V30 2 1 2 3
M  V30 3 2 3 4
M  V30 4 1 4 5
M  V30 5 1 5 6
M  V30 6 2 6 7
M  V30 7 1 7 8
M  V30 8 2 8 9
M  V30 9 1 9 10
M  V30 10 2 10 11
M  V30 11 1 11 12
M  V30 12 1 12 13
M  V30 13 1 13 14
M  V30 14 1 9 14
M  V30 15 2 14 15
M  V30 16 1 6 15
M  V30 17 1 15 16
M  V30 18 2 16 17
M  V30 19 1 17 18
M  V30 20 2 5 18
M  V30 21 1 1 18
M  V30 22 1 20 21
M  V30 23 1 19 21
M  V30 24 1 22 23
M  V30 25 2 19 24
M  V30 26 1 19 25
M  V30 27 1 21 22
M  V30 28 1 23 2
M  V30 29 1 13 26
M  V30 30 1 12 27
M  V30 31 1 1 28
M  V30 END BOND
M  V30 END CTAB
M  END
```

Chrysene cysteinylglycine I A

```
Chrysene cysteinylglycine I A.mol.sb-cf5d4b74-iF0kdI
  ChemDraw04102410512D

  0  0  0     0  0              0 V3000
M  V30 BEGIN CTAB
M  V30 COUNTS 29 32 0 0 0
M  V30 BEGIN ATOM
M  V30 1 O 2.496481 -0.205906 0.000000 0
M  V30 2 C 2.496481 0.617719 0.000000 0
M  V30 3 N 3.209761 1.029531 0.000000 0
M  V30 4 C 3.923041 0.617719 0.000000 0
M  V30 5 C 4.636321 1.029531 0.000000 0
M  V30 6 O 5.349601 0.617719 0.000000 0
M  V30 7 O 4.636321 1.853156 0.000000 0
M  V30 8 C 1.783200 1.029531 0.000000 0
M  V30 9 C 1.069920 0.617719 0.000000 0
M  V30 10 S 0.356640 1.029531 0.000000 0
M  V30 11 N 1.783200 1.853156 0.000000 0
M  V30 12 C -4.636321 -1.853156 0.000000 0
M  V30 13 C -5.349601 -1.441344 0.000000 0
M  V30 14 C -5.349601 -0.617719 0.000000 0
M  V30 15 C -4.636321 -0.205906 0.000000 0
M  V30 16 C -3.923041 -0.617719 0.000000 0
M  V30 17 C -3.209761 -0.205906 0.000000 0
M  V30 18 C -3.209761 0.617719 0.000000 0
M  V30 19 C -2.496480 1.029531 0.000000 0
M  V30 20 C -1.783200 0.617719 0.000000 0
M  V30 21 C -1.069920 1.029531 0.000000 0
M  V30 22 C -0.356640 0.617719 0.000000 0
M  V30 23 C -0.356640 -0.205906 0.000000 0
M  V30 24 C -1.069920 -0.617719 0.000000 0
M  V30 25 C -1.783200 -0.205906 0.000000 0
M  V30 26 C -2.496480 -0.617719 0.000000 0
M  V30 27 C -2.496480 -1.441344 0.000000 0
M  V30 28 C -3.209761 -1.853156 0.000000 0
M  V30 29 C -3.923041 -1.441344 0.000000 0
M  V30 END ATOM
M  V30 BEGIN BOND
M  V30 1 2 1 2
M  V30 2 1 2 3
M  V30 3 1 3 4
M  V30 4 1 4 5
M  V30 5 1 5 6
M  V30 6 2 5 7
M  V30 7 1 2 8
M  V30 8 1 8 9
M  V30 9 1 9 10
M  V30 10 1 8 11
M  V30 11 1 12 13
M  V30 12 2 13 14
M  V30 13 1 14 15
M  V30 14 2 15 16
M  V30 15 1 16 17
M  V30 16 2 17 18
M  V30 17 1 18 19
M  V30 18 2 19 20
M  V30 19 1 20 21
M  V30 20 2 21 22
M  V30 21 1 22 23
M  V30 22 2 23 24
M  V30 23 1 24 25
M  V30 24 1 20 25
M  V30 25 2 25 26
M  V30 26 1 17 26
M  V30 27 1 26 27
M  V30 28 2 27 28
M  V30 29 1 28 29
M  V30 30 1 16 29
M  V30 31 2 12 29
M  V30 32 1 22 10
M  V30 END BOND
M  V30 END CTAB
M  END
```

Chrysene cysteinylglycine I B

```
Chrysene cysteinylglycine I B.mol.sb-cf5d4b74-CCMmVX
  ChemDraw04102410522D

  0  0  0     0  0              0 V3000
M  V30 BEGIN CTAB
M  V30 COUNTS 29 32 0 0 0
M  V30 BEGIN ATOM
M  V30 1 O 1.426560 0.205906 0.000000 0
M  V30 2 C 1.426560 -0.617719 0.000000 0
M  V30 3 N 2.139840 -1.029531 0.000000 0
M  V30 4 C 2.853121 -0.617719 0.000000 0
M  V30 5 C 3.566401 -1.029531 0.000000 0
M  V30 6 O 4.279681 -0.617719 0.000000 0
M  V30 7 O 3.566401 -1.853156 0.000000 0
M  V30 8 C 0.713280 -1.029531 0.000000 0
M  V30 9 C 0.000000 -0.617719 0.000000 0
M  V30 10 S -0.713280 -1.029531 0.000000 0
M  V30 11 N 0.713280 -1.853156 0.000000 0
M  V30 12 C -3.566401 -1.029531 0.000000 0
M  V30 13 C -4.279681 -0.617719 0.000000 0
M  V30 14 C -4.279681 0.205906 0.000000 0
M  V30 15 C -3.566401 0.617719 0.000000 0
M  V30 16 C -2.853121 0.205906 0.000000 0
M  V30 17 C -2.139840 0.617719 0.000000 0
M  V30 18 C -2.139840 1.441344 0.000000 0
M  V30 19 C -1.426560 1.853156 0.000000 0
M  V30 20 C -0.713280 1.441344 0.000000 0
M  V30 21 C 0.000000 1.853156 0.000000 0
M  V30 22 C 0.713280 1.441344 0.000000 0
M  V30 23 C 0.713280 0.617719 0.000000 0
M  V30 24 C 0.000000 0.205906 0.000000 0
M  V30 25 C -0.713280 0.617719 0.000000 0
M  V30 26 C -1.426560 0.205906 0.000000 0
M  V30 27 C -1.426560 -0.617719 0.000000 0
M  V30 28 C -2.139840 -1.029531 0.000000 0
M  V30 29 C -2.853121 -0.617719 0.000000 0
M  V30 END ATOM
M  V30 BEGIN BOND
M  V30 1 2 1 2
M  V30 2 1 2 3
M  V30 3 1 3 4
M  V30 4 1 4 5
M  V30 5 1 5 6
M  V30 6 2 5 7
M  V30 7 1 2 8
M  V30 8 1 8 9
M  V30 9 1 9 10
M  V30 10 1 8 11
M  V30 11 1 12 13
M  V30 12 2 13 14
M  V30 13 1 14 15
M  V30 14 2 15 16
M  V30 15 1 16 17
M  V30 16 2 17 18
M  V30 17 1 18 19
M  V30 18 2 19 20
M  V30 19 1 20 21
M  V30 20 2 21 22
M  V30 21 1 22 23
M  V30 22 2 23 24
M  V30 23 1 24 25
M  V30 24 1 20 25
M  V30 25 2 25 26
M  V30 26 1 17 26
M  V30 27 1 26 27
M  V30 28 2 27 28
M  V30 29 1 28 29
M  V30 30 1 16 29
M  V30 31 2 12 29
M  V30 32 1 10 27
M  V30 END BOND
M  V30 END CTAB
M  END
```

Chrysene cysteinylglycine II

```
Chrysene cysteinylglycine IV A.mol.sb-cf5d4b74-O6BQPM
  ChemDraw04102411012D

  0  0  0     0  0              0 V3000
M  V30 BEGIN CTAB
M  V30 COUNTS 32 35 0 0 0
M  V30 BEGIN ATOM
M  V30 1 C -0.714471 1.031250 0.000000 0
M  V30 2 C 0.000000 0.618750 0.000000 0
M  V30 3 C 0.000000 -0.206250 0.000000 0
M  V30 4 C -0.714471 -0.618750 0.000000 0
M  V30 5 C -1.428942 -0.206250 0.000000 0
M  V30 6 C -2.143413 -0.618750 0.000000 0
M  V30 7 C -2.143413 -1.443750 0.000000 0
M  V30 8 C -2.857884 -1.856250 0.000000 0
M  V30 9 C -3.572355 -1.443750 0.000000 0
M  V30 10 C -4.286825 -1.856250 0.000000 0
M  V30 11 C -5.001297 -1.443750 0.000000 0
M  V30 12 C -5.001297 -0.618750 0.000000 0
M  V30 13 C -4.286825 -0.206250 0.000000 0
M  V30 14 C -3.572355 -0.618750 0.000000 0
M  V30 15 C -2.857884 -0.206250 0.000000 0
M  V30 16 C -2.857884 0.618750 0.000000 0
M  V30 17 C -2.143413 1.031250 0.000000 0
M  V30 18 C -1.428942 0.618750 0.000000 0
M  V30 19 O 2.857884 -0.206250 0.000000 0
M  V30 20 C 2.857884 0.618750 0.000000 0
M  V30 21 N 3.572355 1.031250 0.000000 0
M  V30 22 C 4.286825 0.618750 0.000000 0
M  V30 23 C 5.001297 1.031250 0.000000 0
M  V30 24 O 5.715768 0.618750 0.000000 0
M  V30 25 O 5.001297 1.856250 0.000000 0
M  V30 26 C 2.143413 1.031250 0.000000 0
M  V30 27 C 1.428942 0.618750 0.000000 0
M  V30 28 S 0.714471 1.031250 0.000000 0
M  V30 29 N 2.143413 1.856250 0.000000 0
M  V30 30 O -4.286825 0.618750 0.000000 0
M  V30 31 O -5.715768 -0.206250 0.000000 0
M  V30 32 O -0.714471 1.856250 0.000000 0
M  V30 END ATOM
M  V30 BEGIN BOND
M  V30 1 1 1 2
M  V30 2 1 2 3
M  V30 3 2 3 4
M  V30 4 1 4 5
M  V30 5 1 5 6
M  V30 6 2 6 7
M  V30 7 1 7 8
M  V30 8 2 8 9
M  V30 9 1 9 10
M  V30 10 2 10 11
M  V30 11 1 11 12
M  V30 12 1 12 13
M  V30 13 1 13 14
M  V30 14 1 9 14
M  V30 15 2 14 15
M  V30 16 1 6 15
M  V30 17 1 15 16
M  V30 18 2 16 17
M  V30 19 1 17 18
M  V30 20 2 5 18
M  V30 21 1 1 18
M  V30 22 2 19 20
M  V30 23 1 20 21
M  V30 24 1 21 22
M  V30 25 1 22 23
M  V30 26 1 23 24
M  V30 27 2 23 25
M  V30 28 1 20 26
M  V30 29 1 26 27
M  V30 30 1 27 28
M  V30 31 1 26 29
M  V30 32 1 2 28
M  V30 33 1 13 30
M  V30 34 1 12 31
M  V30 35 1 1 32
M  V30 END BOND
M  V30 END CTAB
M  END
```

Chrysene glutathione I A

```
Chrysene glutathione I A.mol.sb-cf5d4b74-OsoEVn
  ChemDraw04102411062D

  0  0  0     0  0              0 V3000
M  V30 BEGIN CTAB
M  V30 COUNTS 39 42 0 0 0
M  V30 BEGIN ATOM
M  V30 1 C -0.714471 0.412500 0.000000 0
M  V30 2 C 0.000000 -0.000000 0.000000 0
M  V30 3 C 0.000000 -0.824999 0.000000 0
M  V30 4 C -0.714471 -1.237499 0.000000 0
M  V30 5 C -1.428942 -0.824999 0.000000 0
M  V30 6 C -2.143413 -1.237499 0.000000 0
M  V30 7 C -2.143413 -2.062499 0.000000 0
M  V30 8 C -2.857884 -2.475000 0.000000 0
M  V30 9 C -3.572355 -2.062499 0.000000 0
M  V30 10 C -4.286825 -2.475000 0.000000 0
M  V30 11 C -5.001297 -2.062499 0.000000 0
M  V30 12 C -5.001297 -1.237499 0.000000 0
M  V30 13 C -4.286825 -0.824999 0.000000 0
M  V30 14 C -3.572355 -1.237499 0.000000 0
M  V30 15 C -2.857884 -0.824999 0.000000 0
M  V30 16 C -2.857884 -0.000000 0.000000 0
M  V30 17 C -2.143413 0.412500 0.000000 0
M  V30 18 C -1.428942 -0.000000 0.000000 0
M  V30 19 O 2.857884 1.650000 0.000000 0
M  V30 20 C 2.143413 1.237500 0.000000 0
M  V30 21 N 1.428942 1.650000 0.000000 0
M  V30 22 C 1.428942 2.475000 0.000000 0
M  V30 23 C 0.714471 2.887501 0.000000 0
M  V30 24 O 0.714471 3.712500 0.000000 0
M  V30 25 O 0.000000 2.475000 0.000000 0
M  V30 26 C 2.143413 0.412500 0.000000 0
M  V30 27 C 1.428942 -0.000000 0.000000 0
M  V30 28 S 0.714471 0.412500 0.000000 0
M  V30 29 N 2.857884 -0.000000 0.000000 0
M  V30 30 C 2.857884 -0.824999 0.000000 0
M  V30 31 C 3.572355 -1.237499 0.000000 0
M  V30 32 C 3.572355 -2.062499 0.000000 0
M  V30 33 C 4.286825 -2.475000 0.000000 0
M  V30 34 N 5.001297 -2.062499 0.000000 0
M  V30 35 C 4.286825 -3.300000 0.000000 0
M  V30 36 O 5.001297 -3.712500 0.000000 0
M  V30 37 O 3.572355 -3.712500 0.000000 0
M  V30 38 O 2.143413 -1.237499 0.000000 0
M  V30 39 O -0.714471 1.237500 0.000000 0
M  V30 END ATOM
M  V30 BEGIN BOND
M  V30 1 1 1 2
M  V30 2 1 2 3
M  V30 3 2 3 4
M  V30 4 1 4 5
M  V30 5 1 5 6
M  V30 6 2 6 7
M  V30 7 1 7 8
M  V30 8 2 8 9
M  V30 9 1 9 10
M  V30 10 2 10 11
M  V30 11 1 11 12
M  V30 12 2 12 13
M  V30 13 1 13 14
M  V30 14 1 9 14
M  V30 15 2 14 15
M  V30 16 1 6 15
M  V30 17 1 15 16
M  V30 18 2 16 17
M  V30 19 1 17 18
M  V30 20 2 5 18
M  V30 21 1 1 18
M  V30 22 2 19 20
M  V30 23 1 20 21
M  V30 24 1 21 22
M  V30 25 1 22 23
M  V30 26 1 23 24
M  V30 27 2 23 25
M  V30 28 1 20 26
M  V30 29 1 26 27
M  V30 30 1 27 28
M  V30 31 1 26 29
M  V30 32 1 29 30
M  V30 33 1 30 31
M  V30 34 1 31 32
M  V30 35 1 32 33
M  V30 36 1 33 34
M  V30 37 1 33 35
M  V30 38 1 35 36
M  V30 39 2 35 37
M  V30 40 2 30 38
M  V30 41 1 2 28
M  V30 42 1 1 39
M  V30 END BOND
M  V30 END CTAB
M  END
```

Chrysene glutathione I B

```
Chrysene glutathione I B.mol.sb-cf5d4b74-UGklUf
  ChemDraw04102411072D

  0  0  0     0  0              0 V3000
M  V30 BEGIN CTAB
M  V30 COUNTS 39 42 0 0 0
M  V30 BEGIN ATOM
M  V30 1 O 1.428942 -1.650000 0.000000 0
M  V30 2 C 0.714471 -1.237500 0.000000 0
M  V30 3 N 0.000000 -1.650000 0.000000 0
M  V30 4 C 0.000000 -2.475000 0.000000 0
M  V30 5 C -0.714471 -2.887500 0.000000 0
M  V30 6 O -0.714471 -3.712500 0.000000 0
M  V30 7 O -1.428942 -2.475000 0.000000 0
M  V30 8 C 0.714471 -0.412500 0.000000 0
M  V30 9 C 0.000000 -0.000000 0.000000 0
M  V30 10 S 0.000000 0.825000 0.000000 0
M  V30 11 N 1.428942 -0.000000 0.000000 0
M  V30 12 C 1.428942 0.825000 0.000000 0
M  V30 13 C 2.143413 1.237500 0.000000 0
M  V30 14 C 2.143413 2.062500 0.000000 0
M  V30 15 C 2.857884 2.475000 0.000000 0
M  V30 16 N 3.572355 2.062500 0.000000 0
M  V30 17 C 2.857884 3.300000 0.000000 0
M  V30 18 O 3.572355 3.712500 0.000000 0
M  V30 19 O 2.143413 3.712500 0.000000 0
M  V30 20 O 0.714471 1.237500 0.000000 0
M  V30 21 C -2.857884 0.825000 0.000000 0
M  V30 22 C -3.572355 1.237500 0.000000 0
M  V30 23 C -3.572355 2.062500 0.000000 0
M  V30 24 C -2.857884 2.475000 0.000000 0
M  V30 25 C -2.143413 2.062500 0.000000 0
M  V30 26 C -1.428942 2.475000 0.000000 0
M  V30 27 C -1.428942 3.300000 0.000000 0
M  V30 28 C -0.714471 3.712500 0.000000 0
M  V30 29 C 0.000000 3.300000 0.000000 0
M  V30 30 C 0.714471 3.712500 0.000000 0
M  V30 31 C 1.428942 3.300000 0.000000 0
M  V30 32 C 1.428942 2.475000 0.000000 0
M  V30 33 C 0.714471 2.062500 0.000000 0
M  V30 34 C 0.000000 2.475000 0.000000 0
M  V30 35 C -0.714471 2.062500 0.000000 0
M  V30 36 C -0.714471 1.237500 0.000000 0
M  V30 37 C -1.428942 0.825000 0.000000 0
M  V30 38 C -2.143413 1.237500 0.000000 0
M  V30 39 O -1.428942 0.000000 0.000000 0
M  V30 END ATOM
M  V30 BEGIN BOND
M  V30 1 2 1 2
M  V30 2 1 2 3
M  V30 3 1 3 4
M  V30 4 1 4 5
M  V30 5 1 5 6
M  V30 6 2 5 7
M  V30 7 1 2 8
M  V30 8 1 8 9
M  V30 9 1 9 10
M  V30 10 1 8 11
M  V30 11 1 11 12
M  V30 12 1 12 13
M  V30 13 1 13 14
M  V30 14 1 14 15
M  V30 15 1 15 16
M  V30 16 1 15 17
M  V30 17 1 17 18
M  V30 18 2 17 19
M  V30 19 2 12 20
M  V30 20 1 21 22
M  V30 21 2 22 23
M  V30 22 1 23 24
M  V30 23 2 24 25
M  V30 24 1 25 26
M  V30 25 2 26 27
M  V30 26 1 27 28
M  V30 27 2 28 29
M  V30 28 1 29 30
M  V30 29 2 30 31
M  V30 30 1 31 32
M  V30 31 2 32 33
M  V30 32 1 33 34
M  V30 33 1 29 34
M  V30 34 2 34 35
M  V30 35 1 26 35
M  V30 36 1 35 36
M  V30 37 1 36 37
M  V30 38 1 37 38
M  V30 39 1 25 38
M  V30 40 2 21 38
M  V30 41 1 10 36
M  V30 42 1 37 39
M  V30 END BOND
M  V30 END CTAB
M  END
```

Chrysene glutathione II

```
Chrysene glutathione II.mol
  ChemDraw04222409042D

  0  0  0     0  0              0 V3000
M  V30 BEGIN CTAB
M  V30 COUNTS 40 43 0 0 0
M  V30 BEGIN ATOM
M  V30 1 C -0.714471 0.412500 0.000000 0
M  V30 2 C 0.000000 -0.000000 0.000000 0
M  V30 3 C 0.000000 -0.824999 0.000000 0
M  V30 4 C -0.714471 -1.237499 0.000000 0
M  V30 5 C -1.428942 -0.824999 0.000000 0
M  V30 6 C -2.143413 -1.237499 0.000000 0
M  V30 7 C -2.143413 -2.062499 0.000000 0
M  V30 8 C -2.857884 -2.475000 0.000000 0
M  V30 9 C -3.572355 -2.062499 0.000000 0
M  V30 10 C -4.286825 -2.475000 0.000000 0
M  V30 11 C -5.001297 -2.062499 0.000000 0
M  V30 12 C -5.001297 -1.237499 0.000000 0
M  V30 13 C -4.286825 -0.824999 0.000000 0
M  V30 14 C -3.572355 -1.237499 0.000000 0
M  V30 15 C -2.857884 -0.824999 0.000000 0
M  V30 16 C -2.857884 -0.000000 0.000000 0
M  V30 17 C -2.143413 0.412500 0.000000 0
M  V30 18 C -1.428942 -0.000000 0.000000 0
M  V30 19 O 2.857884 1.650000 0.000000 0
M  V30 20 C 2.143413 1.237500 0.000000 0
M  V30 21 N 1.428942 1.650000 0.000000 0
M  V30 22 C 1.428942 2.475000 0.000000 0
M  V30 23 C 0.714471 2.887501 0.000000 0
M  V30 24 O 0.714471 3.712500 0.000000 0
M  V30 25 O 0.000000 2.475000 0.000000 0
M  V30 26 C 2.143413 0.412500 0.000000 0
M  V30 27 C 1.428942 -0.000000 0.000000 0
M  V30 28 S 0.714471 0.412500 0.000000 0
M  V30 29 N 2.857884 -0.000000 0.000000 0
M  V30 30 C 2.857884 -0.824999 0.000000 0
M  V30 31 C 3.572355 -1.237499 0.000000 0
M  V30 32 C 3.572355 -2.062499 0.000000 0
M  V30 33 C 4.286825 -2.475000 0.000000 0
M  V30 34 N 5.001297 -2.062499 0.000000 0
M  V30 35 C 4.286825 -3.300000 0.000000 0
M  V30 36 O 5.001297 -3.712500 0.000000 0
M  V30 37 O 3.572355 -3.712500 0.000000 0
M  V30 38 O 2.143413 -1.237499 0.000000 0
M  V30 39 O -4.286825 0.000001 0.000000 0
M  V30 40 O -0.714471 1.237500 0.000000 0
M  V30 END ATOM
M  V30 BEGIN BOND
M  V30 1 1 1 2
M  V30 2 1 2 3
M  V30 3 2 3 4
M  V30 4 1 4 5
M  V30 5 1 5 6
M  V30 6 2 6 7
M  V30 7 1 7 8
M  V30 8 2 8 9
M  V30 9 1 9 10
M  V30 10 2 10 11
M  V30 11 1 11 12
M  V30 12 2 12 13
M  V30 13 1 13 14
M  V30 14 1 9 14
M  V30 15 2 14 15
M  V30 16 1 6 15
M  V30 17 1 15 16
M  V30 18 2 16 17
M  V30 19 1 17 18
M  V30 20 2 5 18
M  V30 21 1 1 18
M  V30 22 2 19 20
M  V30 23 1 20 21
M  V30 24 1 21 22
M  V30 25 1 22 23
M  V30 26 1 23 24
M  V30 27 2 23 25
M  V30 28 1 20 26
M  V30 29 1 26 27
M  V30 30 1 27 28
M  V30 31 1 26 29
M  V30 32 1 29 30
M  V30 33 1 30 31
M  V30 34 1 31 32
M  V30 35 1 32 33
M  V30 36 1 33 34
M  V30 37 1 33 35
M  V30 38 1 35 36
M  V30 39 2 35 37
M  V30 40 2 30 38
M  V30 41 1 2 28
M  V30 42 1 13 39
M  V30 43 1 1 40
M  V30 END BOND
M  V30 END CTAB
M  END
```

Chrysene mercapturic acid

```
Chrysene mercapturic acid I A.mol.sb-cf5d4b74-O3Siwp
  ChemDraw04102411192D

  0  0  0     0  0              0 V3000
M  V30 BEGIN CTAB
M  V30 COUNTS 28 31 0 0 0
M  V30 BEGIN ATOM
M  V30 1 C -0.357235 0.825000 0.000000 0
M  V30 2 C 0.357235 0.412500 0.000000 0
M  V30 3 C 0.357235 -0.412500 0.000000 0
M  V30 4 C -0.357235 -0.825000 0.000000 0
M  V30 5 C -1.071706 -0.412500 0.000000 0
M  V30 6 C -1.786177 -0.825000 0.000000 0
M  V30 7 C -1.786177 -1.650000 0.000000 0
M  V30 8 C -2.500648 -2.062500 0.000000 0
M  V30 9 C -3.215118 -1.650000 0.000000 0
M  V30 10 C -3.929590 -2.062500 0.000000 0
M  V30 11 C -4.644060 -1.650000 0.000000 0
M  V30 12 C -4.644060 -0.825000 0.000000 0
M  V30 13 C -3.929590 -0.412500 0.000000 0
M  V30 14 C -3.215118 -0.825000 0.000000 0
M  V30 15 C -2.500648 -0.412500 0.000000 0
M  V30 16 C -2.500648 0.412500 0.000000 0
M  V30 17 C -1.786177 0.825000 0.000000 0
M  V30 18 C -1.071706 0.412500 0.000000 0
M  V30 19 C 2.500648 1.650000 0.000000 0
M  V30 20 C 2.500648 0.825000 0.000000 0
M  V30 21 C 1.786177 0.412500 0.000000 0
M  V30 22 S 1.071706 0.825000 0.000000 0
M  V30 23 N 3.215118 0.412500 0.000000 0
M  V30 24 C 3.929590 0.825000 0.000000 0
M  V30 25 C 4.644060 0.412500 0.000000 0
M  V30 26 O 3.929590 1.650000 0.000000 0
M  V30 27 O 1.786177 2.062500 0.000000 0
M  V30 28 O 3.215118 2.062500 0.000000 0
M  V30 END ATOM
M  V30 BEGIN BOND
M  V30 1 2 1 2
M  V30 2 1 2 3
M  V30 3 2 3 4
M  V30 4 1 4 5
M  V30 5 1 5 6
M  V30 6 2 6 7
M  V30 7 1 7 8
M  V30 8 2 8 9
M  V30 9 1 9 10
M  V30 10 2 10 11
M  V30 11 1 11 12
M  V30 12 2 12 13
M  V30 13 1 13 14
M  V30 14 1 9 14
M  V30 15 2 14 15
M  V30 16 1 6 15
M  V30 17 1 15 16
M  V30 18 2 16 17
M  V30 19 1 17 18
M  V30 20 2 5 18
M  V30 21 1 1 18
M  V30 22 1 19 20
M  V30 23 1 21 22
M  V30 24 1 20 23
M  V30 25 1 23 24
M  V30 26 1 24 25
M  V30 27 2 24 26
M  V30 28 2 19 27
M  V30 29 1 19 28
M  V30 30 1 22 2
M  V30 31 1 21 20
M  V30 END BOND
M  V30 END CTAB
M  END
```

Dibenz(a,h)anthracene glutathione I A

```
Dibenz(a,h)anthracene glutathione I A.mol.sb-cf5d4b74-ireYuW
  ChemDraw04092420502D

  0  0  0     0  0              0 V3000
M  V30 BEGIN CTAB
M  V30 COUNTS 43 47 0 0 0
M  V30 BEGIN ATOM
M  V30 1 O -1.428942 3.093750 0.000000 0
M  V30 2 C -1.428942 2.268750 0.000000 0
M  V30 3 N -2.143413 1.856250 0.000000 0
M  V30 4 C -2.857884 2.268750 0.000000 0
M  V30 5 C -3.572355 1.856250 0.000000 0
M  V30 6 O -4.286826 2.268750 0.000000 0
M  V30 7 O -3.572355 1.031250 0.000000 0
M  V30 8 C -0.714471 1.856250 0.000000 0
M  V30 9 C -0.714471 1.031250 0.000000 0
M  V30 10 S -1.428942 0.618750 0.000000 0
M  V30 11 N -0.000000 2.268750 0.000000 0
M  V30 12 C 0.714471 1.856250 0.000000 0
M  V30 13 C 1.428942 2.268750 0.000000 0
M  V30 14 C 2.143413 1.856250 0.000000 0
M  V30 15 C 2.857884 2.268750 0.000000 0
M  V30 16 N 2.857884 3.093750 0.000000 0
M  V30 17 C 3.572355 1.856250 0.000000 0
M  V30 18 O 4.286826 2.268750 0.000000 0
M  V30 19 O 3.572355 1.031250 0.000000 0
M  V30 20 O 0.714471 1.031250 0.000000 0
M  V30 21 C 1.428942 -0.206250 0.000000 0
M  V30 22 C 2.143413 -0.618750 0.000000 0
M  V30 23 C 2.143413 -1.443750 0.000000 0
M  V30 24 C 1.428942 -1.856250 0.000000 0
M  V30 25 C 0.714471 -1.443750 0.000000 0
M  V30 26 C -0.000000 -1.856250 0.000000 0
M  V30 27 C -0.714471 -1.443750 0.000000 0
M  V30 28 C -1.428942 -1.856250 0.000000 0
M  V30 29 C -2.143413 -1.443750 0.000000 0
M  V30 30 C -2.143413 -0.618750 0.000000 0
M  V30 31 C -1.428942 -0.206250 0.000000 0
M  V30 32 C -0.714471 -0.618750 0.000000 0
M  V30 33 C -0.000000 -0.206250 0.000000 0
M  V30 34 C 0.714471 -0.618750 0.000000 0
M  V30 35 C 2.857884 -0.206250 0.000000 0
M  V30 36 C 2.857884 0.618750 0.000000 0
M  V30 37 C 2.143413 1.031250 0.000000 0
M  V30 38 C 1.428942 0.618750 0.000000 0
M  V30 39 C -2.857884 -1.856250 0.000000 0
M  V30 40 C -2.857884 -2.681250 0.000000 0
M  V30 41 C -2.143413 -3.093750 0.000000 0
M  V30 42 C -1.428942 -2.681250 0.000000 0
M  V30 43 O -2.857884 -0.206250 0.000000 0
M  V30 END ATOM
M  V30 BEGIN BOND
M  V30 1 2 1 2
M  V30 2 1 2 3
M  V30 3 1 3 4
M  V30 4 1 4 5
M  V30 5 1 5 6
M  V30 6 2 5 7
M  V30 7 1 2 8
M  V30 8 1 8 9
M  V30 9 1 9 10
M  V30 10 1 8 11
M  V30 11 1 11 12
M  V30 12 1 12 13
M  V30 13 1 13 14
M  V30 14 1 14 15
M  V30 15 1 15 16
M  V30 16 1 15 17
M  V30 17 1 17 18
M  V30 18 2 17 19
M  V30 19 2 12 20
M  V30 20 1 21 22
M  V30 21 1 22 23
M  V30 22 2 23 24
M  V30 23 1 24 25
M  V30 24 2 25 26
M  V30 25 1 26 27
M  V30 26 1 27 28
M  V30 27 2 28 29
M  V30 28 1 29 30
M  V30 29 1 30 31
M  V30 30 1 31 32
M  V30 31 2 27 32
M  V30 32 1 32 33
M  V30 33 2 33 34
M  V30 34 1 25 34
M  V30 35 1 21 34
M  V30 36 1 35 36
M  V30 37 2 36 37
M  V30 38 1 37 38
M  V30 39 2 38 21
M  V30 40 2 35 22
M  V30 41 2 39 40
M  V30 42 1 40 41
M  V30 43 2 41 42
M  V30 44 1 42 28
M  V30 45 1 39 29
M  V30 46 1 30 43
M  V30 47 1 31 10
M  V30 END BOND
M  V30 END CTAB
M  END
```

Dibenz(a,h)anthracene glutathione I B

```
Dibenz(a,h)anthracene glutathione I B.mol.sb-cf5d4b74-NlWliH
  ChemDraw04092420512D

  0  0  0     0  0              0 V3000
M  V30 BEGIN CTAB
M  V30 COUNTS 43 47 0 0 0
M  V30 BEGIN ATOM
M  V30 1 C -0.713280 -1.441343 0.000000 0
M  V30 2 C 0.000000 -1.853156 0.000000 0
M  V30 3 C 0.000000 -2.676782 0.000000 0
M  V30 4 C -0.713280 -3.088594 0.000000 0
M  V30 5 C -1.426560 -2.676782 0.000000 0
M  V30 6 C -2.139840 -3.088594 0.000000 0
M  V30 7 C -2.853121 -2.676782 0.000000 0
M  V30 8 C -3.566401 -3.088594 0.000000 0
M  V30 9 C -4.279681 -2.676782 0.000000 0
M  V30 10 C -4.279681 -1.853156 0.000000 0
M  V30 11 C -3.566401 -1.441343 0.000000 0
M  V30 12 C -2.853121 -1.853156 0.000000 0
M  V30 13 C -2.139840 -1.441344 0.000000 0
M  V30 14 C -1.426560 -1.853156 0.000000 0
M  V30 15 C 0.713280 -1.441343 0.000000 0
M  V30 16 C 0.713280 -0.617719 0.000000 0
M  V30 17 C 0.000000 -0.205907 0.000000 0
M  V30 18 C -0.713280 -0.617719 0.000000 0
M  V30 19 C -4.992961 -3.088594 0.000000 0
M  V30 20 C -4.992961 -3.912219 0.000000 0
M  V30 21 C -4.279681 -4.324031 0.000000 0
M  V30 22 C -3.566401 -3.912219 0.000000 0
M  V30 23 O 2.139840 -0.617719 0.000000 0
M  V30 24 O 3.566400 -3.088594 0.000000 0
M  V30 25 O 4.992961 -3.088594 0.000000 0
M  V30 26 C 4.279680 -2.676781 0.000000 0
M  V30 27 N 4.992961 -1.441344 0.000000 0
M  V30 28 C 4.279680 -1.853156 0.000000 0
M  V30 29 C 3.566400 -1.441344 0.000000 0
M  V30 30 C 3.566400 -0.617719 0.000000 0
M  V30 31 C 2.853120 -0.205907 0.000000 0
M  V30 32 N 2.853120 0.617718 0.000000 0
M  V30 33 S 1.426560 -0.205907 0.000000 0
M  V30 34 C 1.426560 0.617718 0.000000 0
M  V30 35 C 2.139840 1.029531 0.000000 0
M  V30 36 O -0.000000 3.088594 0.000000 0
M  V30 37 O 0.713280 4.324031 0.000000 0
M  V30 38 C 0.713280 3.500406 0.000000 0
M  V30 39 C 1.426560 3.088594 0.000000 0
M  V30 40 N 1.426560 2.264969 0.000000 0
M  V30 41 C 2.139840 1.853156 0.000000 0
M  V30 42 O 2.853120 2.264969 0.000000 0
M  V30 43 O 1.426560 -1.853156 0.000000 0
M  V30 END ATOM
M  V30 BEGIN BOND
M  V30 1 2 31 23
M  V30 2 2 26 24
M  V30 3 1 26 25
M  V30 4 1 28 26
M  V30 5 1 28 27
M  V30 6 1 29 28
M  V30 7 1 30 29
M  V30 8 1 31 30
M  V30 9 1 32 31
M  V30 10 1 35 32
M  V30 11 1 34 33
M  V30 12 1 35 34
M  V30 13 1 41 35
M  V30 14 2 38 36
M  V30 15 1 38 37
M  V30 16 1 39 38
M  V30 17 1 40 39
M  V30 18 1 41 40
M  V30 19 2 42 41
M  V30 20 2 1 2
M  V30 21 1 2 3
M  V30 22 2 3 4
M  V30 23 1 4 5
M  V30 24 2 5 6
M  V30 25 1 6 7
M  V30 26 2 7 8
M  V30 27 1 8 9
M  V30 28 2 9 10
M  V30 29 1 10 11
M  V30 30 2 11 12
M  V30 31 1 7 12
M  V30 32 1 12 13
M  V30 33 2 13 14
M  V30 34 1 5 14
M  V30 35 1 1 14
M  V30 36 1 15 16
M  V30 37 1 16 17
M  V30 38 2 17 18
M  V30 39 1 18 1
M  V30 40 1 15 2
M  V30 41 2 19 20
M  V30 42 1 20 21
M  V30 43 2 21 22
M  V30 44 1 22 8
M  V30 45 1 19 9
M  V30 46 1 15 43
M  V30 47 1 33 16
M  V30 END BOND
M  V30 END CTAB
M  END
```

Dibenz(a,h)anthracene glutathione II

```
Dibenz(a,h)anthracene glutathione III.mol.sb-cf5d4b74-kyMJPJ
  ChemDraw04092420482D

  0  0  0     0  0              0 V3000
M  V30 BEGIN CTAB
M  V30 COUNTS 45 49 0 0 0
M  V30 BEGIN ATOM
M  V30 1 C -0.714471 -1.443750 0.000000 0
M  V30 2 C -0.000001 -1.856250 0.000000 0
M  V30 3 C -0.000001 -2.681251 0.000000 0
M  V30 4 C -0.714471 -3.093751 0.000000 0
M  V30 5 C -1.428943 -2.681251 0.000000 0
M  V30 6 C -2.143412 -3.093751 0.000000 0
M  V30 7 C -2.857883 -2.681251 0.000000 0
M  V30 8 C -3.572354 -3.093751 0.000000 0
M  V30 9 C -4.286825 -2.681251 0.000000 0
M  V30 10 C -4.286825 -1.856250 0.000000 0
M  V30 11 C -3.572354 -1.443750 0.000000 0
M  V30 12 C -2.857883 -1.856250 0.000000 0
M  V30 13 C -2.143412 -1.443750 0.000000 0
M  V30 14 C -1.428943 -1.856250 0.000000 0
M  V30 15 C 0.714469 -1.443750 0.000000 0
M  V30 16 C 0.714469 -0.618750 0.000000 0
M  V30 17 C -0.000001 -0.206251 0.000000 0
M  V30 18 C -0.714471 -0.618750 0.000000 0
M  V30 19 C -5.001296 -3.093751 0.000000 0
M  V30 20 C -5.001296 -3.918749 0.000000 0
M  V30 21 C -4.286825 -4.331250 0.000000 0
M  V30 22 C -3.572354 -3.918749 0.000000 0
M  V30 23 O 4.286825 -0.206250 0.000000 0
M  V30 24 C 3.572354 -0.618750 0.000000 0
M  V30 25 N 3.572354 -1.443750 0.000000 0
M  V30 26 C 4.286825 -1.856250 0.000000 0
M  V30 27 C 4.286825 -2.681250 0.000000 0
M  V30 28 O 5.001296 -3.093750 0.000000 0
M  V30 29 O 3.572354 -3.093750 0.000000 0
M  V30 30 C 2.857882 -0.206250 0.000000 0
M  V30 31 C 2.143411 -0.618750 0.000000 0
M  V30 32 S 1.428940 -0.206250 0.000000 0
M  V30 33 N 2.857882 0.618750 0.000000 0
M  V30 34 C 2.143411 1.031250 0.000000 0
M  V30 35 C 2.143411 1.856250 0.000000 0
M  V30 36 C 1.428940 2.268750 0.000000 0
M  V30 37 C 1.428940 3.093750 0.000000 0
M  V30 38 N 2.143411 3.506250 0.000000 0
M  V30 39 C 0.714469 3.506250 0.000000 0
M  V30 40 O 0.714469 4.331250 0.000000 0
M  V30 41 O -0.000001 3.093750 0.000000 0
M  V30 42 O 1.428940 0.618750 0.000000 0
M  V30 43 O -5.001296 -1.443750 0.000000 0
M  V30 44 O -3.572354 -0.618750 0.000000 0
M  V30 45 O 1.428940 -1.856250 0.000000 0
M  V30 END ATOM
M  V30 BEGIN BOND
M  V30 1 2 1 2
M  V30 2 1 2 3
M  V30 3 2 3 4
M  V30 4 1 4 5
M  V30 5 2 5 6
M  V30 6 1 6 7
M  V30 7 1 7 8
M  V30 8 1 8 9
M  V30 9 1 9 10
M  V30 10 1 10 11
M  V30 11 1 11 12
M  V30 12 2 7 12
M  V30 13 1 12 13
M  V30 14 2 13 14
M  V30 15 1 5 14
M  V30 16 1 1 14
M  V30 17 1 15 16
M  V30 18 1 16 17
M  V30 19 2 17 18
M  V30 20 1 18 1
M  V30 21 1 15 2
M  V30 22 1 19 20
M  V30 23 2 20 21
M  V30 24 1 21 22
M  V30 25 2 22 8
M  V30 26 2 19 9
M  V30 27 2 23 24
M  V30 28 1 24 25
M  V30 29 1 25 26
M  V30 30 1 26 27
M  V30 31 1 27 28
M  V30 32 2 27 29
M  V30 33 1 24 30
M  V30 34 1 30 31
M  V30 35 1 31 32
M  V30 36 1 30 33
M  V30 37 1 33 34
M  V30 38 1 34 35
M  V30 39 1 35 36
M  V30 40 1 36 37
M  V30 41 1 37 38
M  V30 42 1 37 39
M  V30 43 1 39 40
M  V30 44 2 39 41
M  V30 45 2 34 42
M  V30 46 1 10 43
M  V30 47 1 11 44
M  V30 48 1 15 45
M  V30 49 1 16 32
M  V30 END BOND
M  V30 END CTAB
M  END
```

Phenanthrene cysteinylglycine

```
Phenanthrene cysteinylglycine A.mol.sb-cf5d4b74-WSZSWd
  ChemDraw04092420262D

  0  0  0     0  0              0 V3000
M  V30 BEGIN CTAB
M  V30 COUNTS 25 27 0 0 0
M  V30 BEGIN ATOM
M  V30 1 N 0.000000 1.850063 0.000000 0
M  V30 2 S 1.424179 1.027813 0.000000 0
M  V30 3 C 0.712090 0.616688 0.000000 0
M  V30 4 C 0.000000 1.027813 0.000000 0
M  V30 5 O -2.848358 1.850063 0.000000 0
M  V30 6 O -3.560447 0.616688 0.000000 0
M  V30 7 C -2.848358 1.027813 0.000000 0
M  V30 8 C -2.136268 0.616688 0.000000 0
M  V30 9 N -1.424179 1.027813 0.000000 0
M  V30 10 C -0.712090 0.616688 0.000000 0
M  V30 11 O -0.712090 -0.205562 0.000000 0
M  V30 12 C 1.424179 -1.438937 0.000000 0
M  V30 13 C 2.136268 -1.850063 0.000000 0
M  V30 14 C 2.848358 -1.438937 0.000000 0
M  V30 15 C 2.848358 -0.616687 0.000000 0
M  V30 16 C 3.560447 -0.205562 0.000000 0
M  V30 17 C 3.560447 0.616688 0.000000 0
M  V30 18 C 2.848358 1.027813 0.000000 0
M  V30 19 C 2.136268 0.616688 0.000000 0
M  V30 20 C 2.136268 -0.205562 0.000000 0
M  V30 21 C 1.424179 -0.616687 0.000000 0
M  V30 22 C 0.712090 -0.205562 0.000000 0
M  V30 23 C 0.000000 -0.616687 0.000000 0
M  V30 24 C 0.000000 -1.438937 0.000000 0
M  V30 25 C 0.712090 -1.850063 0.000000 0
M  V30 END ATOM
M  V30 BEGIN BOND
M  V30 1 1 4 1
M  V30 2 1 3 2
M  V30 3 1 4 3
M  V30 4 1 10 4
M  V30 5 2 7 5
M  V30 6 1 7 6
M  V30 7 1 8 7
M  V30 8 1 9 8
M  V30 9 1 10 9
M  V30 10 2 11 10
M  V30 11 1 21 12
M  V30 12 2 25 12
M  V30 13 1 13 12
M  V30 14 2 14 13
M  V30 15 1 15 14
M  V30 16 1 20 15
M  V30 17 2 16 15
M  V30 18 1 17 16
M  V30 19 2 18 17
M  V30 20 1 19 18
M  V30 21 2 20 19
M  V30 22 1 21 20
M  V30 23 2 22 21
M  V30 24 1 23 22
M  V30 25 2 24 23
M  V30 26 1 25 24
M  V30 27 1 19 2
M  V30 END BOND
M  V30 END CTAB
M  END
```

Phenanthrene glutathione


```
Phenanthrene glutathione A.mol.sb-cf5d4b74-12V42P
  ChemDraw04092420142D

  0  0  0     0  0              0 V3000
M  V30 BEGIN CTAB
M  V30 COUNTS 36 38 0 0 0
M  V30 BEGIN ATOM
M  V30 1 C -0.357236 0.000000 0.000000 0
M  V30 2 C -0.357236 0.824998 0.000000 0
M  V30 3 C -1.071708 1.237499 0.000000 0
M  V30 4 C -1.786178 0.824998 0.000000 0
M  V30 5 C -1.786178 -0.000002 0.000000 0
M  V30 6 C -2.500650 -0.412500 0.000000 0
M  V30 7 C -3.215118 -0.000001 0.000000 0
M  V30 8 C -3.929590 -0.412500 0.000000 0
M  V30 9 C -3.929590 -1.237501 0.000000 0
M  V30 10 C -3.215119 -1.650001 0.000000 0
M  V30 11 C -2.500650 -1.237501 0.000000 0
M  V30 12 C -1.786178 -1.650001 0.000000 0
M  V30 13 C -1.071708 -1.237501 0.000000 0
M  V30 14 C -1.071708 -0.412500 0.000000 0
M  V30 15 S 0.357235 -0.412500 0.000000 0
M  V30 16 O 2.500648 -1.650000 0.000000 0
M  V30 17 C 1.786177 -1.237500 0.000000 0
M  V30 18 N 1.071706 -1.650000 0.000000 0
M  V30 19 C 1.071706 -2.475000 0.000000 0
M  V30 20 C 0.357235 -2.887500 0.000000 0
M  V30 21 O 0.357235 -3.712500 0.000000 0
M  V30 22 O -0.357236 -2.475000 0.000000 0
M  V30 23 C 1.786177 -0.412500 0.000000 0
M  V30 24 C 1.071706 0.000000 0.000000 0
M  V30 25 N 2.500648 0.000000 0.000000 0
M  V30 26 C 2.500648 0.825000 0.000000 0
M  V30 27 C 3.215119 1.237500 0.000000 0
M  V30 28 C 3.215119 2.062500 0.000000 0
M  V30 29 C 3.929590 2.475000 0.000000 0
M  V30 30 N 4.644061 2.062500 0.000000 0
M  V30 31 C 3.929590 3.300000 0.000000 0
M  V30 32 O 4.644061 3.712500 0.000000 0
M  V30 33 O 3.215119 3.712500 0.000000 0
M  V30 34 O 1.786177 1.237500 0.000000 0
M  V30 35 O -3.215118 0.824999 0.000000 0
M  V30 36 O -4.644061 0.000000 0.000000 0
M  V30 END ATOM
M  V30 BEGIN BOND
M  V30 1 1 1 2
M  V30 2 2 2 3
M  V30 3 1 3 4
M  V30 4 2 4 5
M  V30 5 1 5 6
M  V30 6 1 6 7
M  V30 7 1 7 8
M  V30 8 1 8 9
M  V30 9 2 9 10
M  V30 10 1 10 11
M  V30 11 2 6 11
M  V30 12 1 11 12
M  V30 13 2 12 13
M  V30 14 1 13 14
M  V30 15 2 1 14
M  V30 16 1 5 14
M  V30 17 1 24 15
M  V30 18 2 16 17
M  V30 19 1 17 18
M  V30 20 1 18 19
M  V30 21 1 19 20
M  V30 22 1 20 21
M  V30 23 2 20 22
M  V30 24 1 17 23
M  V30 25 1 23 24
M  V30 26 1 23 25
M  V30 27 1 25 26
M  V30 28 1 26 27
M  V30 29 1 27 28
M  V30 30 1 28 29
M  V30 31 1 29 30
M  V30 32 1 29 31
M  V30 33 1 31 32
M  V30 34 2 31 33
M  V30 35 2 26 34
M  V30 36 2 7 35
M  V30 37 2 8 36
M  V30 38 1 1 15
M  V30 END BOND
M  V30 END CTAB
M  END
```
