# Supplementary material for: Exposure of Polycyclic Aromatic Hydrocarbons (PAHs) and Crude Oil to Atlantic Haddock (Melanogrammus aeglefinus): A Unique Snapshot of the Mercapturic Acid Pathway
Source: Environ Sci Technol. 2024 Aug 5;58(33):14855–63. doi: 10.1021/acs.est.4c05112 (PMC11340023; doi:10.1021/acs.est.4c05112)
Supplement: Supplementary file 2 — es4c05112_si_002.zip [file es4c05112_si_002.zip › library/index.html]

 all\_html


- 1,4-Dimethylphenanthrene cysteinylglycine A
- 1,4-Dimethylphenanthrene cysteinylglycine B
- 1,4-Dimethylphenanthrene glutathione I
- 1,4-Dimethylphenanthrene glutathione II
- 1-Methylphenanthrene cysteinylglycine A
- 1-Methylphenanthrene cysteinylglycine B
- 1-Methylphenanthrene glutathione I
- 1-Methylphenanthrene glutathione II
- Anthracene cysteinylglycine
- Benz(a)anthracene cysteinylglycine I A
- Benz(a)anthracene cysteinylglycine I B
- Benz(a)anthracene cysteinylglycine II
- Benz(a)anthracene cysteinylglycine III
- Benz(a)anthracene cysteinylglycine IV A
- Benz(a)anthracene cysteinylglycine IV B
- Benz(a)anthracene glutathione I A
- Benz(a)anthracene glutathione I B
- Benz(a)anthracene glutathione II
- Benzo(a)pyrene cysteinylglycine A
- Benzo(a)pyrene cysteinylglycine B
- Chrysene cysteine
- Chrysene cysteinylglycine I A
- Chrysene cysteinylglycine I B
- Chrysene cysteinylglycine II
- Chrysene glutathione I A
- Chrysene glutathione I B
- Chrysene glutathione II
- Chrysene mercapturic acid
- Dibenz(a,h)anthracene glutathione I A
- Dibenz(a,h)anthracene glutathione I B
- Dibenz(a,h)anthracene glutathione II
- Phenanthrene cysteinylglycine
- Phenanthrene glutathione

Metabolite library

  

---

## 1,4-Dimethylphenanthrene cysteinylglycine A

  

| Compound data | Proposed structure |
| --- | --- |
| **Conjugate**: cysteinylglycine **Treatment**: 1,4-dimethylphenanthrene **Formula**: C21H22N2O3S **Neutral mass (Da)**: 382.1351 **Adduct**: -H **Expected mass (Da)**: 381.1278 **Observed mass (Da)**: 381.1284 **Predicted CCS (Å2)**: 197.24 **Observed CCS (Å2)**: 200.21 **Δ CCS (%)**: 1.5 **Observed drift time (ms)**: 6.73 **Observed retention time (min)**: 5.5 |  |

SMILES: OC(=O)CNC(=O)C(CSc1ccc2c(c1)ccc1c2c(C)ccc1C)N mol

**Mass spectra**:

*Low energy (top) and high energy (bottom) mass spectra*

  

---

## 1,4-Dimethylphenanthrene cysteinylglycine B

  

| Compound data | Proposed structure |
| --- | --- |
| **Conjugate**: cysteinylglycine **Treatment**: 1,4-dimethylphenanthrene **Formula**: C21H22N2O3S **Neutral mass (Da)**: 382.1351 **Adduct**: -H **Expected mass (Da)**: 381.1278 **Observed mass (Da)**: 381.1281 **Predicted CCS (Å2)**: 192.67 **Observed CCS (Å2)**: 194.36 **Δ CCS (%)**: 0.9 **Observed drift time (ms)**: 6.50 **Observed retention time (min)**: 7.5 |  |

SMILES: OC(=O)CNC(=O)C(CSc1cc2ccccc2c2c1c(C)ccc2C)N mol

**Mass spectra**:

*Low energy (top) and high energy (bottom) mass spectra*

  

---

## 1,4-Dimethylphenanthrene glutathione I

  

| Compound data | Proposed structure |
| --- | --- |
| **Conjugate**: glutathione **Treatment**: 1,4-dimethylphenanthrene **Formula**: C26H29N3O6S **Neutral mass (Da)**: 511.1777 **Adduct**: -H **Expected mass (Da)**: 510.1704 **Observed mass (Da)**: 510.1708 **Predicted CCS (Å2)**: 222.69 **Observed CCS (Å2)**: 217.24 **Δ CCS (%)**: -2.4 **Observed drift time (ms)**: 7.42 **Observed retention time (min)**: 7.6 |  |

SMILES: O=C(NC(C(=O)NCC(=O)O)CSc1ccc2c(c1)ccc1c2c(C)ccc1C)CCC(C(=O)O)N mol

**Mass spectra**:

*Low energy (top) and high energy (bottom) mass spectra*

  

---

## 1,4-Dimethylphenanthrene glutathione II

  

| Compound data | Proposed structure |
| --- | --- |
| **Conjugate**: glutathione **Treatment**: 1,4-dimethylphenanthrene **Formula**: C26H31N3O7S **Neutral mass (Da)**: 529.1883 **Adduct**: -H **Expected mass (Da)**: 528.1810 **Observed mass (Da)**: 528.1816 **Predicted CCS (Å2)**: 226.54 **Observed CCS (Å2)**: 219.22 **Δ CCS (%)**: -3.2 **Observed drift time (ms)**: 7.51 **Observed retention time (min)**: 5.3 |  |

SMILES: O=C(NC(C(=O)NCC(=O)O)CSC1C=Cc2c(C1O)ccc1c2c(C)ccc1C)CCC(C(=O)O)N mol

**Mass spectra**:

*Low energy (top) and high energy (bottom) mass spectra*

  

---

## 1-Methylphenanthrene cysteinylglycine A

  

| Compound data | Proposed structure |
| --- | --- |
| **Conjugate**: cysteinylglycine **Treatment**: 1-methylphenanthrene **Formula**: C20H20N2O3S **Neutral mass (Da)**: 368.1195 **Adduct**: -H **Expected mass (Da)**: 367.1122 **Observed mass (Da)**: 367.1121 **Predicted CCS (Å2)**: 189.01 **Observed CCS (Å2)**: 191.70 **Δ CCS (%)**: 1.4 **Observed drift time (ms)**: 6.39 **Observed retention time (min)**: 5.0 |  |

SMILES: OC(=O)CNC(=O)C(CSc1ccc(c2c1c1ccccc1cc2)C)N mol

**Mass spectra**:

*Low energy (top) and high energy (bottom) mass spectra*

  

---

## 1-Methylphenanthrene cysteinylglycine B

  

| Compound data | Proposed structure |
| --- | --- |
| **Conjugate**: cysteinylglycine **Treatment**: 1-methylphenanthrene **Formula**: C20H20N2O3S **Neutral mass (Da)**: 368.1195 **Adduct**: -H **Expected mass (Da)**: 367.1122 **Observed mass (Da)**: 367.1127 **Predicted CCS (Å2)**: 188.80 **Observed CCS (Å2)**: 192.08 **Δ CCS (%)**: 1.7 **Observed drift time (ms)**: 6.40 **Observed retention time (min)**: 8.5 |  |

SMILES: OC(=O)CNC(=O)C(CSc1cc2ccccc2c2c1c(C)ccc2)N mol

**Mass spectra**:

*Low energy (top) and high energy (bottom) mass spectra*

  

---

## 1-Methylphenanthrene glutathione I

  

| Compound data | Proposed structure |
| --- | --- |
| **Conjugate**: glutathione **Treatment**: 1-methylphenanthrene **Formula**: C25H27N3O6S **Neutral mass (Da)**: 497.1621 **Adduct**: -H **Expected mass (Da)**: 496.1548 **Observed mass (Da)**: 496.1547 **Predicted CCS (Å2)**: 217.56 **Observed CCS (Å2)**: 213.51 **Δ CCS (%)**: -1.9 **Observed drift time (ms)**: 7.27 **Observed retention time (min)**: 5.8 |  |

SMILES: O=C(NC(C(=O)NCC(=O)O)CSc1ccc(c2c1c1ccccc1cc2)C)CCC(C(=O)O)N mol

**Mass spectra**:

*Low energy (top) and high energy (bottom) mass spectra*

  

---

## 1-Methylphenanthrene glutathione II

  

| Compound data | Proposed structure |
| --- | --- |
| **Conjugate**: glutathione **Treatment**: 1-methylphenanthrene **Formula**: C25H29N3O7S **Neutral mass (Da)**: 515.1726 **Adduct**: -H **Expected mass (Da)**: 514.1653 **Observed mass (Da)**: 514.1657 **Predicted CCS (Å2)**: 221.32 **Observed CCS (Å2)**: 218.20 **Δ CCS (%)**: -1.4 **Observed drift time (ms)**: 7.46 **Observed retention time (min)**: 2.2 |  |

SMILES: O=C(NC(C(=O)NCC(=O)O)CSC1C(O)C=C(c2c1c1ccccc1cc2)C)CCC(C(=O)O)N mol

**Mass spectra**:

*Low energy (top) and high energy (bottom) mass spectra*

  

---

## Anthracene cysteinylglycine

  

| Compound data | Proposed structure |
| --- | --- |
| **Conjugate**: cysteinylglycine **Treatment**: anthracene **Formula**: C19H18N2O3S **Neutral mass (Da)**: 354.1038 **Adduct**: -H **Expected mass (Da)**: 353.0965 **Observed mass (Da)**: 353.0972 **Predicted CCS (Å2)**: 185.41 **Observed CCS (Å2)**: 189.08 **Δ CCS (%)**: 2.0 **Observed drift time (ms)**: 6.27 **Observed retention time (min)**: 2.5 |  |

SMILES: OC(=O)CNC(=O)C(CSc1cccc2c1cc1ccccc1c2)N mol

**Mass spectra**:

*Low energy (top) and high energy (bottom) mass spectra*

  

---

## Benz(a)anthracene cysteinylglycine I A

  

| Compound data | Proposed structure |
| --- | --- |
| **Conjugate**: cysteinylglycine **Treatment**: benz(a)anthracene **Formula**: C23H20N2O3S **Neutral mass (Da)**: 404.1195 **Adduct**: -H **Expected mass (Da)**: 403.1122 **Observed mass (Da)**: 403.1123 **Predicted CCS (Å2)**: 200.23 **Observed CCS (Å2)**: 203.38 **Δ CCS (%)**: 1.6 **Observed drift time (ms)**: 6.86 **Observed retention time (min)**: 6.2 |  |

SMILES: OC(=O)CNC(=O)C(CSc1ccc2c(c1)ccc1c2cc2ccccc2c1)N mol

**Mass spectra**:

*Low energy (top) and high energy (bottom) mass spectra*

  

---

## Benz(a)anthracene cysteinylglycine I B

  

| Compound data | Proposed structure |
| --- | --- |
| **Conjugate**: cysteinylglycine **Treatment**: benz(a)anthracene **Formula**: C23H20N2O3S **Neutral mass (Da)**: 404.1195 **Adduct**: -H **Expected mass (Da)**: 403.1122 **Observed mass (Da)**: 403.1122 **Predicted CCS (Å2)**: 200.21 **Observed CCS (Å2)**: 204.83 **Δ CCS (%)**: 2.3 **Observed drift time (ms)**: 6.92 **Observed retention time (min)**: 5.9 |  |

SMILES: OC(=O)CNC(=O)C(CSc1ccc2c(c1)cc1c(c2)ccc2c1cccc2)N mol

**Mass spectra**:

*Low energy (top) and high energy (bottom) mass spectra*

  

---

## Benz(a)anthracene cysteinylglycine II

  

| Compound data | Proposed structure |
| --- | --- |
| **Conjugate**: cysteinylglycine **Treatment**: benz(a)anthracene **Formula**: C23H22N2O4S **Neutral mass (Da)**: 422.1300 **Adduct**: -H **Expected mass (Da)**: 421.1228 **Observed mass (Da)**: 421.1230 **Predicted CCS (Å2)**: 202.97 **Observed CCS (Å2)**: 203.92 **Δ CCS (%)**: 0.5 **Observed drift time (ms)**: 6.89 **Observed retention time (min)**: 4.4 |  |

SMILES: OC(=O)CNC(=O)C(CSC1C=Cc2c(C1O)ccc1c2cc2ccccc2c1)N mol

**Mass spectra**:

*Low energy (top) and high energy (bottom) mass spectra*

  

---

## Benz(a)anthracene cysteinylglycine III

  

| Compound data | Proposed structure |
| --- | --- |
| **Conjugate**: cysteinylglycine **Treatment**: benz(a)anthracene **Formula**: C23H22N2O5S **Neutral mass (Da)**: 438.1249 **Adduct**: -H **Expected mass (Da)**: 437.1177 **Observed mass (Da)**: 437.1181 **Predicted CCS (Å2)**: 205.70 **Observed CCS (Å2)**: 196.72 **Δ CCS (%)**: -4.4 **Observed drift time (ms)**: 6.60 **Observed retention time (min)**: 2.2 |  |

SMILES: OC(=O)CNC(=O)C(CSc1ccc2c(c1)ccc1c2cc2c(c1)C=CC(C2O)O)N mol

**Mass spectra**:

*Low energy (top) and high energy (bottom) mass spectra*

  

---

## Benz(a)anthracene cysteinylglycine IV A

  

| Compound data | Proposed structure |
| --- | --- |
| **Conjugate**: cysteinylglycine **Treatment**: benz(a)anthracene **Formula**: C23H24N2O6S **Neutral mass (Da)**: 456.1355 **Adduct**: -H **Expected mass (Da)**: 455.1282 **Observed mass (Da)**: 455.1289 **Predicted CCS (Å2)**: 208.46 **Observed CCS (Å2)**: 208.64 **Δ CCS (%)**: 0.1 **Observed drift time (ms)**: 7.08 **Observed retention time (min)**: 2.8 |  |

SMILES: OC(=O)CNC(=O)C(CSC1C=Cc2c(C1O)ccc1c2cc2c(c1)C=CC(C2O)O)N mol

**Mass spectra**:

*Low energy (top) and high energy (bottom) mass spectra*

  

---

## Benz(a)anthracene cysteinylglycine IV B

  

| Compound data | Proposed structure |
| --- | --- |
| **Conjugate**: cysteinylglycine **Treatment**: benz(a)anthracene **Formula**: C23H24N2O6S **Neutral mass (Da)**: 456.1355 **Adduct**: -H **Expected mass (Da)**: 455.1282 **Observed mass (Da)**: 455.1283 **Predicted CCS (Å2)**: 208.43 **Observed CCS (Å2)**: 209.13 **Δ CCS (%)**: 0.3 **Observed drift time (ms)**: 7.10 **Observed retention time (min)**: 3.7 |  |

SMILES: OC(=O)CNC(=O)C(CSC1C=Cc2c(C1O)cc1c(c2)ccc2c1C=CC(C2O)O)N mol

**Mass spectra**:

*Low energy (top) and high energy (bottom) mass spectra*

  

---

## Benz(a)anthracene glutathione I A

  

| Compound data | Proposed structure |
| --- | --- |
| **Conjugate**: glutathione **Treatment**: benz(a)anthracene **Formula**: C28H29N3O7S **Neutral mass (Da)**: 551.1726 **Adduct**: -H **Expected mass (Da)**: 550.1653 **Observed mass (Da)**: 550.1654 **Predicted CCS (Å2)**: 231.64 **Observed CCS (Å2)**: 232.77 **Δ CCS (%)**: 0.5 **Observed drift time (ms)**: 8.03 **Observed retention time (min)**: 3.2 |  |

SMILES: O=C(NC(C(=O)NCC(=O)O)CSC1C=Cc2c(C1O)ccc1c2cc2ccccc2c1)CCC(C(=O)O)N mol

**Mass spectra**:

*Low energy (top) and high energy (bottom) mass spectra*

  

---

## Benz(a)anthracene glutathione I B

  

| Compound data | Proposed structure |
| --- | --- |
| **Conjugate**: glutathione **Treatment**: benz(a)anthracene **Formula**: C28H29N3O7S **Neutral mass (Da)**: 551.1726 **Adduct**: -H **Expected mass (Da)**: 550.1653 **Observed mass (Da)**: 550.1656 **Predicted CCS (Å2)**: 231.53 **Observed CCS (Å2)**: 227.05 **Δ CCS (%)**: -1.9 **Observed drift time (ms)**: 7.81 **Observed retention time (min)**: 2.5 |  |

SMILES: O=C(NC(C(=O)NCC(=O)O)CSC1C=Cc2c(C1O)cc1c(c2)ccc2c1cccc2)CCC(C(=O)O)N mol

**Mass spectra**:

*Low energy (top) and high energy (bottom) mass spectra*

  

---

## Benz(a)anthracene glutathione II

  

| Compound data | Proposed structure |
| --- | --- |
| **Conjugate**: glutathione **Treatment**: benz(a)anthracene **Formula**: C28H29N3O8S **Neutral mass (Da)**: 567.1675 **Adduct**: -H **Expected mass (Da)**: 566.1603 **Observed mass (Da)**: 566.1601 **Predicted CCS (Å2)**: 234.52 **Observed CCS (Å2)**: 235.78 **Δ CCS (%)**: 0.5 **Observed drift time (ms)**: 8.15 **Observed retention time (min)**: 2.2 |  |

SMILES: O=C(NC(C(=O)NCC(=O)O)CSC1C=Cc2c(C1O)ccc1c2cc2c(O)cccc2c1)CCC(C(=O)O)N mol

**Mass spectra**:

*Low energy (top) and high energy (bottom) mass spectra*

  

---

## Benzo(a)pyrene cysteinylglycine A

  

| Compound data | Proposed structure |
| --- | --- |
| **Conjugate**: cysteinylglycine **Treatment**: benzo(a)pyrene **Formula**: C25H24N2O6S **Neutral mass (Da)**: 480.1355 **Adduct**: -H **Expected mass (Da)**: 479.1282 **Observed mass (Da)**: 479.1288 **Predicted CCS (Å2)**: 213.38 **Observed CCS (Å2)**: 211.86 **Δ CCS (%)**: -0.7 **Observed drift time (ms)**: 7.21 **Observed retention time (min)**: 4.2 |  |

SMILES: OC(=O)CNC(=O)C(CSC1C(O)C(O)C(c2c1c1ccc3c4c1c(c2)ccc4ccc3)O)N mol

**Mass spectra**:

*Low energy (top) and high energy (bottom) mass spectra*

  

---

## Benzo(a)pyrene cysteinylglycine B

  

| Compound data | Proposed structure |
| --- | --- |
| **Conjugate**: cysteinylglycine **Treatment**: benzo(a)pyrene **Formula**: C25H24N2O6S **Neutral mass (Da)**: 480.1355 **Adduct**: -H **Expected mass (Da)**: 479.1282 **Observed mass (Da)**: 479.1288 **Predicted CCS (Å2)**: 214.66 **Observed CCS (Å2)**: 213.66 **Δ CCS (%)**: -0.5 **Observed drift time (ms)**: 7.28 **Observed retention time (min)**: 5.2 |  |

SMILES: OC(=O)CNC(=O)C(CSC1C(O)C=Cc2c1c1ccc3c4c1c(c2)ccc4=CC(C3O)O)N mol

**Mass spectra**:

*Low energy (top) and high energy (bottom) mass spectra*

  

---

## Chrysene cysteine

  

| Compound data | Proposed structure |
| --- | --- |
| **Conjugate**: cysteine **Treatment**: chrysene **Formula**: C21H21NO5S **Neutral mass (Da)**: 399.1140 **Adduct**: -H **Expected mass (Da)**: 398.1068 **Observed mass (Da)**: 398.1076 **Predicted CCS (Å2)**: 199.66 **Observed CCS (Å2)**: 194.41 **Δ CCS (%)**: -2.6 **Observed drift time (ms)**: 6.49 **Observed retention time (min)**: 4.1 |  |

SMILES: OC(=O)C(CSC1C=Cc2c(C1O)ccc1c2ccc2c1C(O)C(C=C2)O)N mol

**Mass spectra**:

*Low energy (top) and high energy (bottom) mass spectra*

  

---

## Chrysene cysteinylglycine I A

  

| Compound data | Proposed structure |
| --- | --- |
| **Conjugate**: cysteinylglycine **Treatment**: chrysene **Formula**: C23H20N2O3S **Neutral mass (Da)**: 404.1195 **Adduct**: -H **Expected mass (Da)**: 403.1122 **Observed mass (Da)**: 403.1128 **Predicted CCS (Å2)**: 200.23 **Observed CCS (Å2)**: 202.05 **Δ CCS (%)**: 0.9 **Observed drift time (ms)**: 6.81 **Observed retention time (min)**: 4.8 |  |

SMILES: OC(=O)CNC(=O)C(CSc1ccc2c(c1)ccc1c2ccc2c1cccc2)N mol

**Mass spectra**:

*Low energy (top) and high energy (bottom) mass spectra*

  

---

## Chrysene cysteinylglycine I B

  

| Compound data | Proposed structure |
| --- | --- |
| **Conjugate**: cysteinylglycine **Treatment**: chrysene **Formula**: C23H20N2O3S **Neutral mass (Da)**: 404.1195 **Adduct**: -H **Expected mass (Da)**: 403.1122 **Observed mass (Da)**: 403.1127 **Predicted CCS (Å2)**: 198.84 **Observed CCS (Å2)**: 204.83 **Δ CCS (%)**: 3.0 **Observed drift time (ms)**: 6.92 **Observed retention time (min)**: 5.8 |  |

SMILES: OC(=O)CNC(=O)C(CSc1cc2ccccc2c2c1c1ccccc1cc2)N mol

**Mass spectra**:

*Low energy (top) and high energy (bottom) mass spectra*

  

---

## Chrysene cysteinylglycine II

  

| Compound data | Proposed structure |
| --- | --- |
| **Conjugate**: cysteinylglycine **Treatment**: chrysene **Formula**: C23H24N2O6S **Neutral mass (Da)**: 456.1355 **Adduct**: -H **Expected mass (Da)**: 455.1282 **Observed mass (Da)**: 455.1290 **Predicted CCS (Å2)**: 208.42 **Observed CCS (Å2)**: 208.01 **Δ CCS (%)**: -0.2 **Observed drift time (ms)**: 7.04 **Observed retention time (min)**: 3.9 |  |

SMILES: OC(=O)CNC(=O)C(CSC1C=Cc2c(C1O)ccc1c2ccc2c1C(O)C(C=C2)O)N mol

**Mass spectra**:

*Low energy (top) and high energy (bottom) mass spectra*

  

---

## Chrysene glutathione I A

  

| Compound data | Proposed structure |
| --- | --- |
| **Conjugate**: glutathione **Treatment**: chrysene **Formula**: C28H29N3O7S **Neutral mass (Da)**: 551.1726 **Adduct**: -H **Expected mass (Da)**: 550.1653 **Observed mass (Da)**: 550.1659 **Predicted CCS (Å2)**: 231.64 **Observed CCS (Å2)**: 230.76 **Δ CCS (%)**: -0.4 **Observed drift time (ms)**: 7.95 **Observed retention time (min)**: 2.3 |  |

SMILES: O=C(NC(C(=O)NCC(=O)O)CSC1C=Cc2c(C1O)ccc1c2ccc2c1cccc2)CCC(C(=O)O)N mol

**Mass spectra**:

*Low energy (top) and high energy (bottom) mass spectra*

  

---

## Chrysene glutathione I B

  

| Compound data | Proposed structure |
| --- | --- |
| **Conjugate**: glutathione **Treatment**: chrysene **Formula**: C28H29N3O7S **Neutral mass (Da)**: 551.1726 **Adduct**: -H **Expected mass (Da)**: 550.1653 **Observed mass (Da)**: 550.1662 **Predicted CCS (Å2)**: 230.66 **Observed CCS (Å2)**: 232.71 **Δ CCS (%)**: 0.9 **Observed drift time (ms)**: 8.03 **Observed retention time (min)**: 2.9 |  |

SMILES: O=C(NC(C(=O)NCC(=O)O)CSC1C(O)c2ccccc2-c2c1c1ccccc1cc2)CCC(C(=O)O)N mol

**Mass spectra**:

*Low energy (top) and high energy (bottom) mass spectra*

  

---

## Chrysene glutathione II

  

| Compound data | Proposed structure |
| --- | --- |
| **Conjugate**: glutathione **Treatment**: chrysene **Formula**: C28H29N3O8S **Neutral mass (Da)**: 567.1675 **Adduct**: -H **Expected mass (Da)**: 566.1603 **Observed mass (Da)**: 566.1609 **Predicted CCS (Å2)**: 234.50 **Observed CCS (Å2)**: 221.02 **Δ CCS (%)**: -5.7 **Observed drift time (ms)**: 7.58 **Observed retention time (min)**: 1.5 |  |

SMILES: O=C(NC(C(=O)NCC(=O)O)CSC1C=Cc2c(C1O)ccc1c2ccc2c1c(O)ccc2)CCC(C(=O)O)N mol

**Mass spectra**:

*Low energy (top) and high energy (bottom) mass spectra*

  

---

## Chrysene mercapturic acid

  

| Compound data | Proposed structure |
| --- | --- |
| **Conjugate**: mercapturic **Treatment**: chrysene **Formula**: C23H19NO3S **Neutral mass (Da)**: 389.1086 **Adduct**: -H **Expected mass (Da)**: 388.1013 **Observed mass (Da)**: 388.1020 **Predicted CCS (Å2)**: 198.06 **Observed CCS (Å2)**: 204.22 **Δ CCS (%)**: 3.1 **Observed drift time (ms)**: 6.89 **Observed retention time (min)**: 4.5 |  |

SMILES: CC(=O)NC(C(=O)O)CSc1ccc2c(c1)ccc1c2ccc2c1cccc2 mol

**Mass spectra**:

*Low energy (top) and high energy (bottom) mass spectra*

  

---

## Dibenz(a,h)anthracene glutathione I A

  

| Compound data | Proposed structure |
| --- | --- |
| **Conjugate**: glutathione **Treatment**: dibenz(a,h)anthracene **Formula**: C32H31N3O7S **Neutral mass (Da)**: 601.1883 **Adduct**: -H **Expected mass (Da)**: 600.1810 **Observed mass (Da)**: 600.1811 **Predicted CCS (Å2)**: 245.02 **Observed CCS (Å2)**: 243.18 **Δ CCS (%)**: -0.7 **Observed drift time (ms)**: 8.43 **Observed retention time (min)**: 5.8 |  |

SMILES: O=C(NC(C(=O)NCC(=O)O)CSC1C(O)c2ccccc2-c2c1cc1c(c2)ccc2c1cccc2)CCC(C(=O)O)N mol

**Mass spectra**:

*Low energy (top) and high energy (bottom) mass spectra*

  

---

## Dibenz(a,h)anthracene glutathione I B

  

| Compound data | Proposed structure |
| --- | --- |
| **Conjugate**: glutathione **Treatment**: dibenz(a,h)anthracene **Formula**: C32H31N3O7S **Neutral mass (Da)**: 601.1883 **Adduct**: -H **Expected mass (Da)**: 600.1810 **Observed mass (Da)**: 600.1811 **Predicted CCS (Å2)**: 246.02 **Observed CCS (Å2)**: 248.13 **Δ CCS (%)**: 0.9 **Observed drift time (ms)**: 8.63 **Observed retention time (min)**: 6.7 |  |

SMILES: O=C(NC(C(=O)NCC(=O)O)CSC1C=Cc2c(C1O)ccc1c2cc2ccc3c(c2c1)cccc3)CCC(C(=O)O)N mol

**Mass spectra**:

*Low energy (top) and high energy (bottom) mass spectra*

  

---

## Dibenz(a,h)anthracene glutathione II

  

| Compound data | Proposed structure |
| --- | --- |
| **Conjugate**: glutathione **Treatment**: dibenz(a,h)anthracene **Formula**: C32H33N3O9S **Neutral mass (Da)**: 635.1938 **Adduct**: -H **Expected mass (Da)**: 634.1865 **Observed mass (Da)**: 634.1866 **Predicted CCS (Å2)**: 253.09 **Observed CCS (Å2)**: 248.58 **Δ CCS (%)**: -1.8 **Observed drift time (ms)**: 8.63 **Observed retention time (min)**: 3.7 |  |

SMILES: O=C(NC(C(=O)NCC(=O)O)CSC1C=Cc2c(C1O)ccc1c2cc2C(O)C(O)c3c(-c2c1)cccc3)CCC(C(=O)O)N mol

**Mass spectra**:

*Low energy (top) and high energy (bottom) mass spectra*

  

---

## Phenanthrene cysteinylglycine

  

| Compound data | Proposed structure |
| --- | --- |
| **Conjugate**: cysteinylglycine **Treatment**: phenanthrene **Formula**: C19H18N2O3S **Neutral mass (Da)**: 354.1038 **Adduct**: -H **Expected mass (Da)**: 353.0965 **Observed mass (Da)**: 353.0974 **Predicted CCS (Å2)**: 183.91 **Observed CCS (Å2)**: 189.75 **Δ CCS (%)**: 3.2 **Observed drift time (ms)**: 6.30 **Observed retention time (min)**: 2.1 |  |

SMILES: OC(=O)CNC(=O)C(CSc1cccc2c1c1ccccc1cc2)N mol

**Mass spectra**:

*Low energy (top) and high energy (bottom) mass spectra*

  

---

## Phenanthrene glutathione

  

| Compound data | Proposed structure |
| --- | --- |
| **Conjugate**: glutathione **Treatment**: phenanthrene **Formula**: C24H23N3O8S **Neutral mass (Da)**: 513.1206 **Adduct**: -H **Expected mass (Da)**: 512.1133 **Observed mass (Da)**: 512.1139 **Predicted CCS (Å2)**: 220.27 **Observed CCS (Å2)**: 212.65 **Δ CCS (%)**: -3.5 **Observed drift time (ms)**: 7.24 **Observed retention time (min)**: 2.9 |  |

SMILES: O=C(NC(C(=O)NCC(=O)O)CSc1cccc2c1ccc1c2C(=O)C(=O)C=C1)CCC(C(=O)O)N mol

**Mass spectra**:

*Low energy (top) and high energy (bottom) mass spectra*
