# Supplementary figures and images for: Exposure of Polycyclic Aromatic Hydrocarbons (PAHs) and Crude Oil to Atlantic Haddock (Melanogrammus aeglefinus): A Unique Snapshot of the Mercapturic Acid Pathway
Source: Environ Sci Technol. 2024 Aug 5;58(33):14855–63. doi: 10.1021/acs.est.4c05112 (PMC11340023; doi:10.1021/acs.est.4c05112)

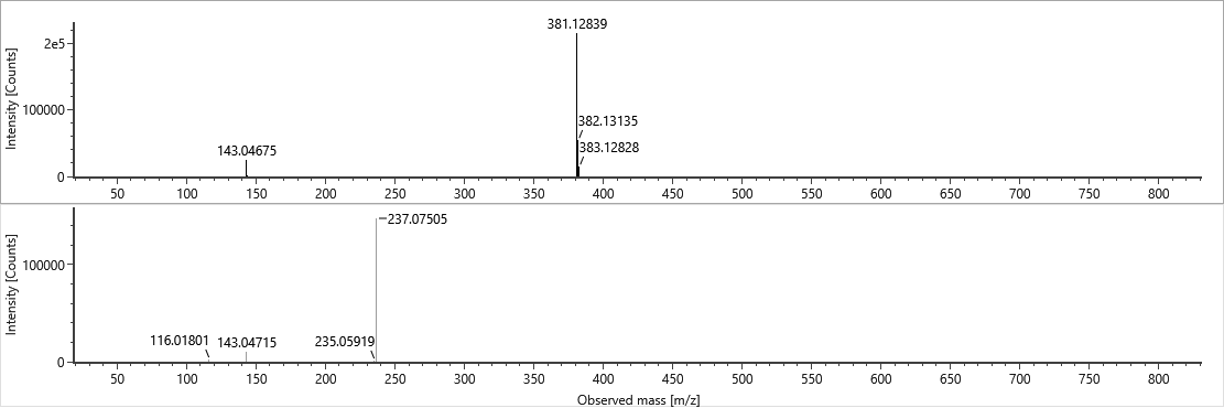

Supplement: Supplementary file 2 — es4c05112_si_002.zip [file es4c05112_si_002.zip › library/png/1,4-dimethylphenanthrene cysteinylglycine A.png]

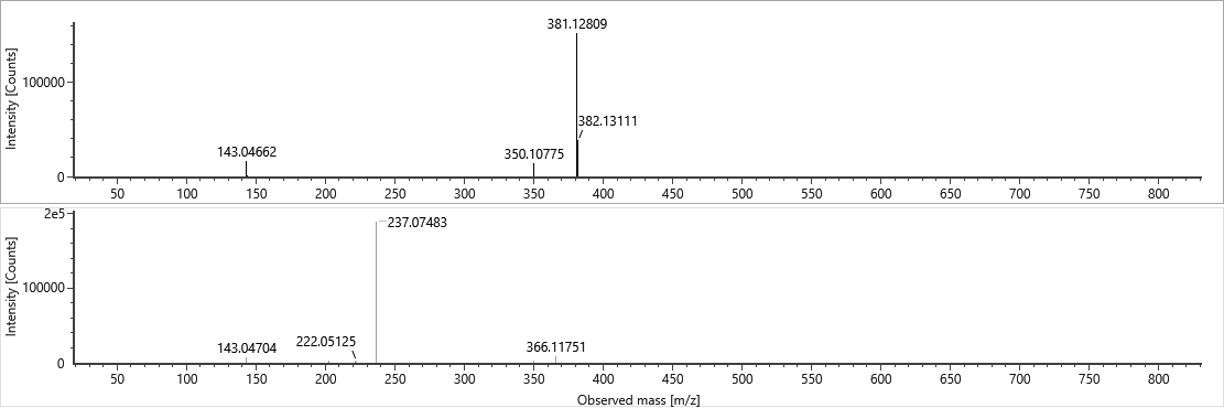

Supplement: Supplementary file 2 — es4c05112_si_002.zip [file es4c05112_si_002.zip › library/png/1,4-dimethylphenanthrene cysteinylglycine B.png]

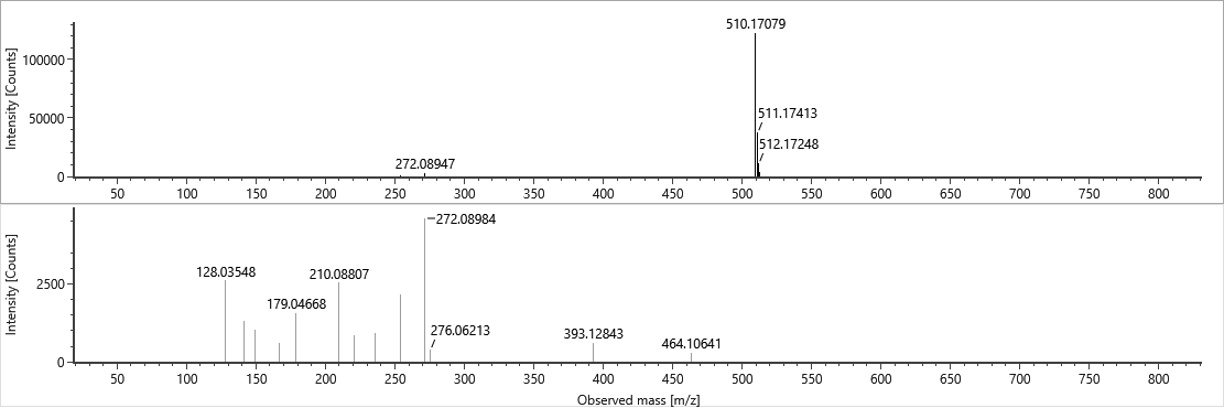

Supplement: Supplementary file 2 — es4c05112_si_002.zip [file es4c05112_si_002.zip › library/png/1,4-dimethylphenanthrene glutathione I.png]

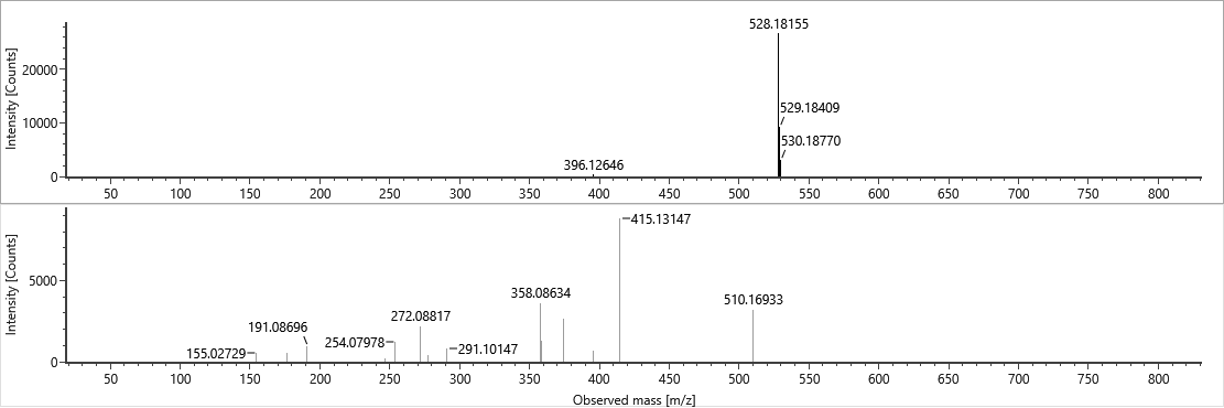

Supplement: Supplementary file 2 — es4c05112_si_002.zip [file es4c05112_si_002.zip › library/png/1,4-dimethylphenanthrene glutathione II.png]

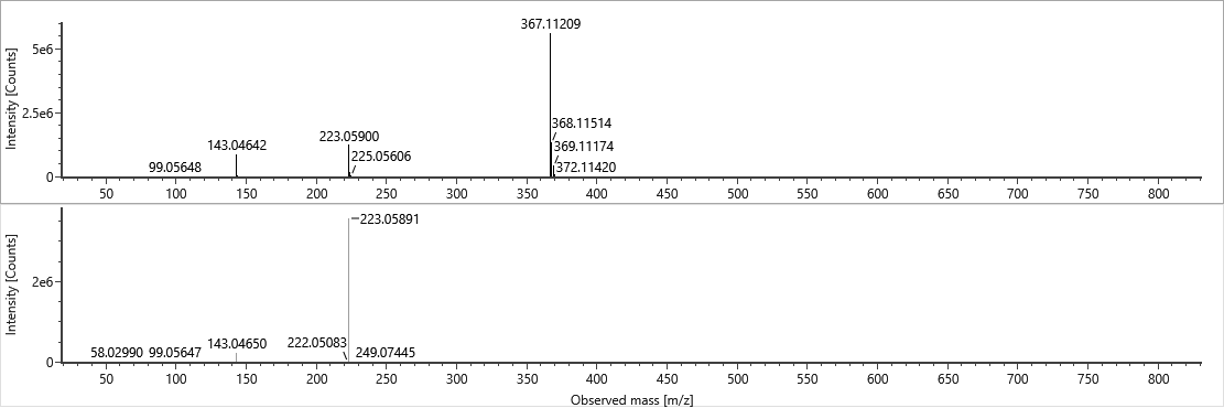

Supplement: Supplementary file 2 — es4c05112_si_002.zip [file es4c05112_si_002.zip › library/png/1-methylphenanthrene cysteinylglycine A.png]

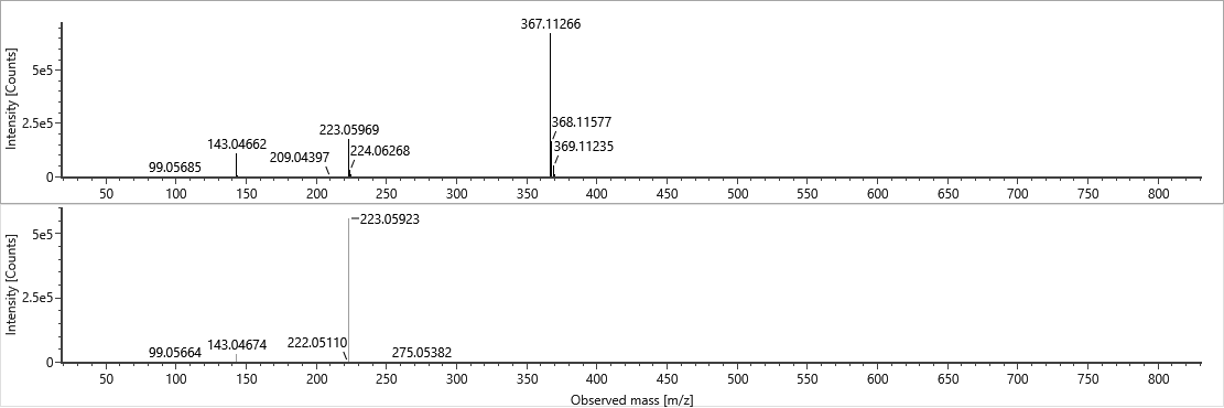

Supplement: Supplementary file 2 — es4c05112_si_002.zip [file es4c05112_si_002.zip › library/png/1-methylphenanthrene cysteinylglycine B.png]

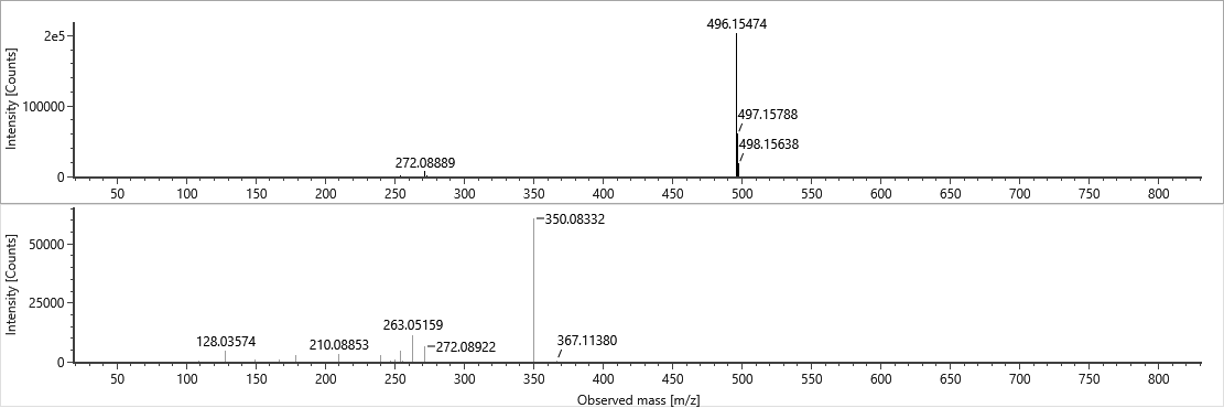

Supplement: Supplementary file 2 — es4c05112_si_002.zip [file es4c05112_si_002.zip › library/png/1-Methylphenanthrene glutathione I.png]

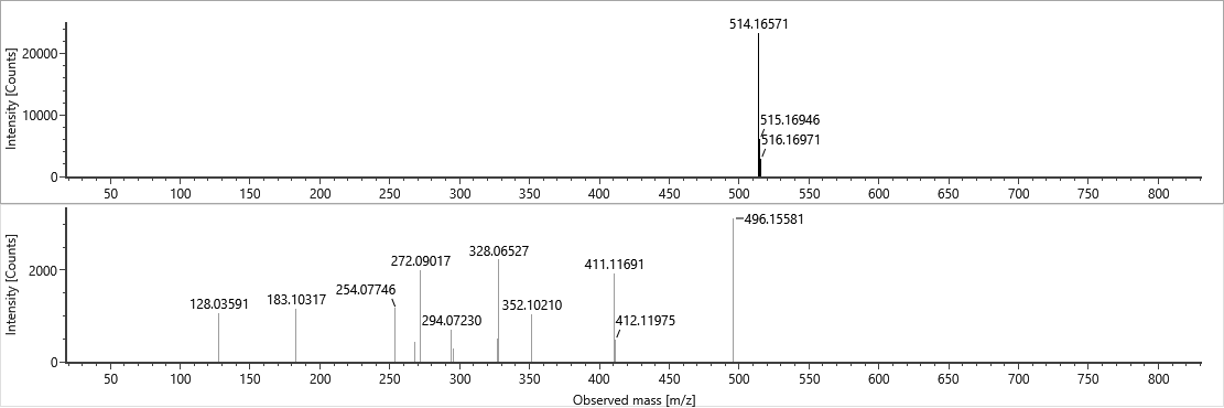

Supplement: Supplementary file 2 — es4c05112_si_002.zip [file es4c05112_si_002.zip › library/png/1-Methylphenanthrene glutathione II.png]

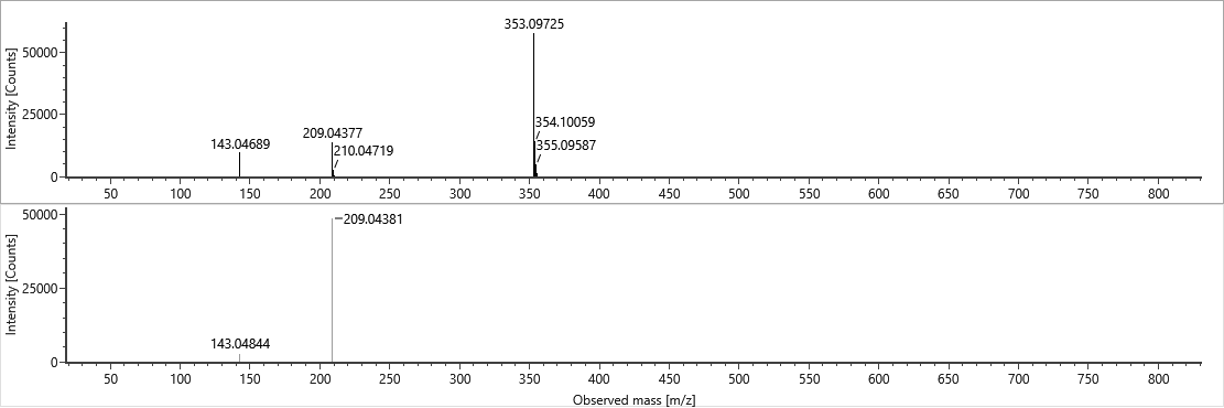

Supplement: Supplementary file 2 — es4c05112_si_002.zip [file es4c05112_si_002.zip › library/png/Anthracene cysteinylglycine.png]

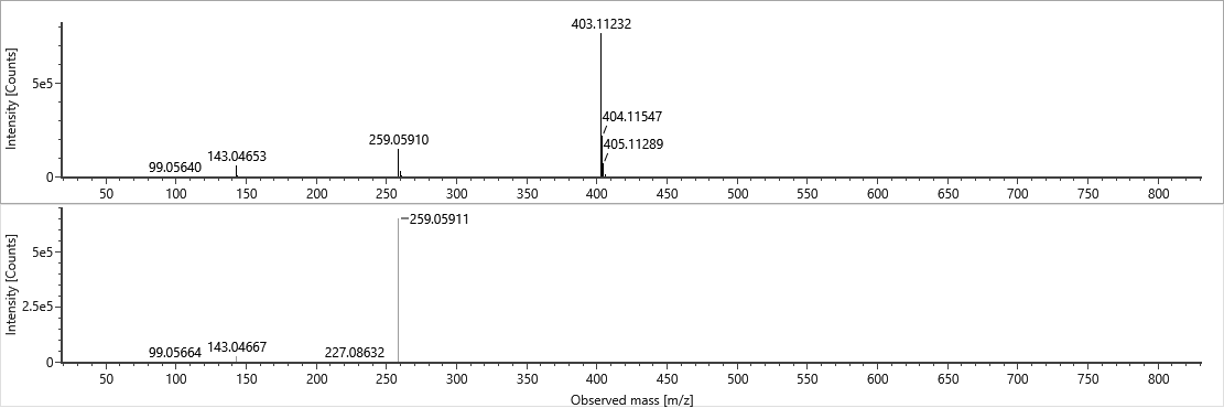

Supplement: Supplementary file 2 — es4c05112_si_002.zip [file es4c05112_si_002.zip › library/png/Benz(a)anthracene cysteinylglycine I A.png]

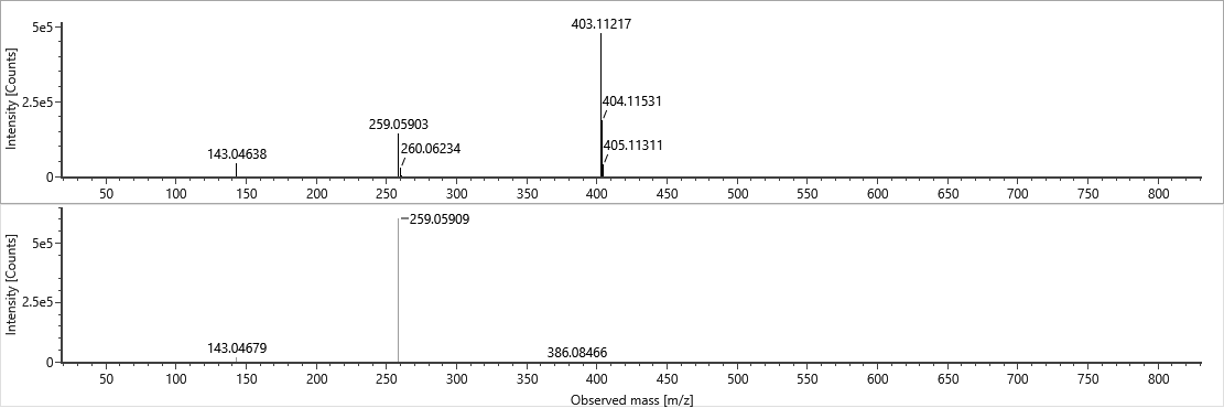

Supplement: Supplementary file 2 — es4c05112_si_002.zip [file es4c05112_si_002.zip › library/png/Benz(a)anthracene cysteinylglycine I B.png]

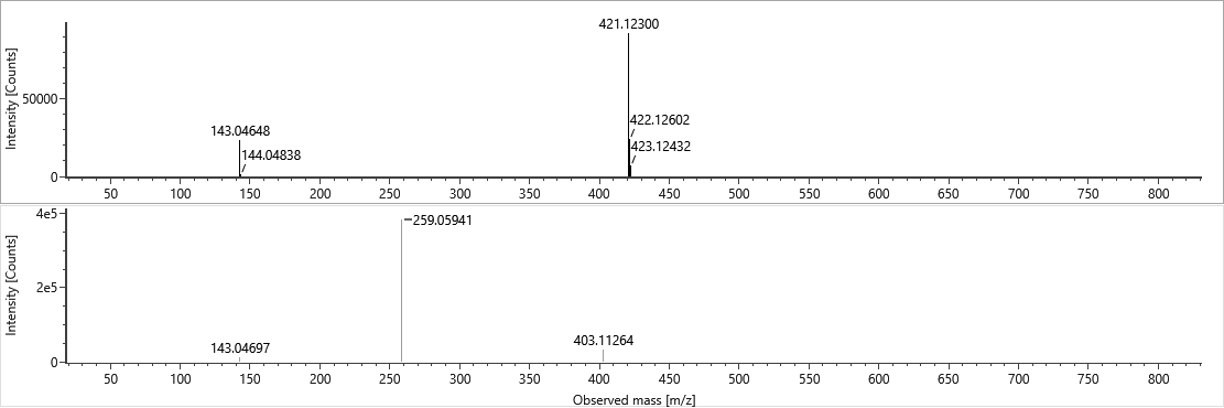

Supplement: Supplementary file 2 — es4c05112_si_002.zip [file es4c05112_si_002.zip › library/png/Benz(a)anthracene cysteinylglycine II.png]

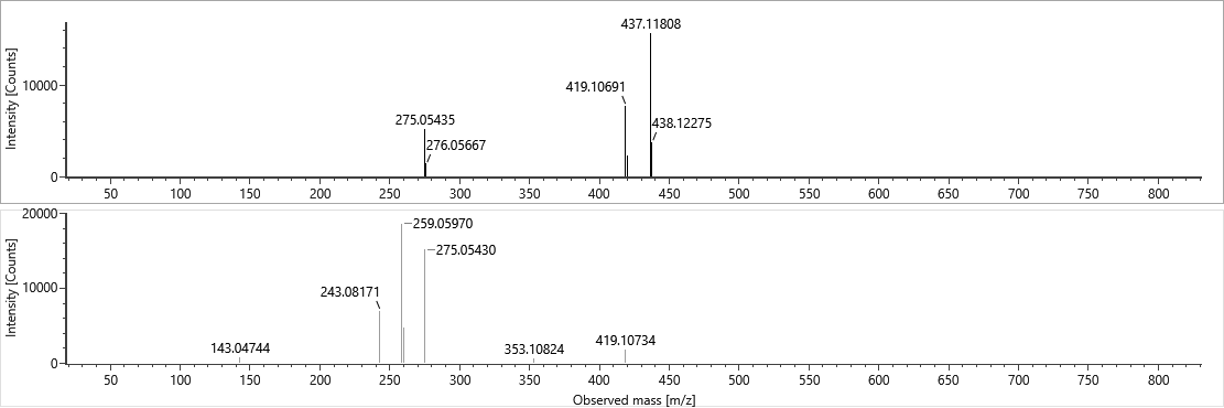

Supplement: Supplementary file 2 — es4c05112_si_002.zip [file es4c05112_si_002.zip › library/png/Benz(a)anthracene cysteinylglycine III.png]

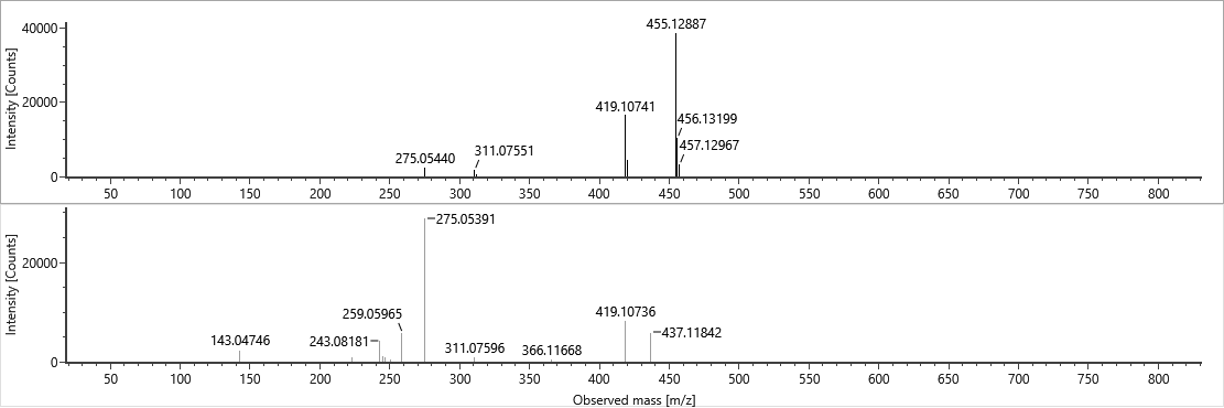

Supplement: Supplementary file 2 — es4c05112_si_002.zip [file es4c05112_si_002.zip › library/png/Benz(a)anthracene cysteinylglycine IV A.png]

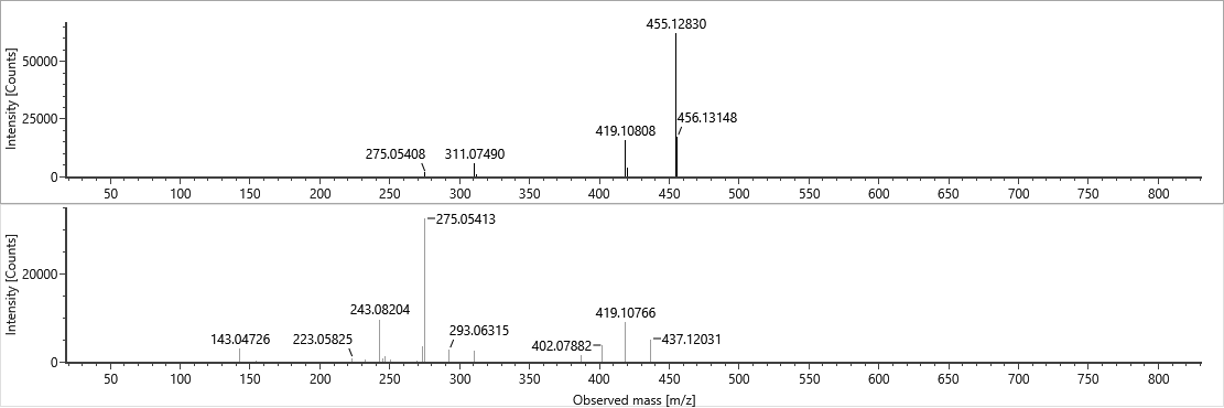

Supplement: Supplementary file 2 — es4c05112_si_002.zip [file es4c05112_si_002.zip › library/png/Benz(a)anthracene cysteinylglycine IV B.png]

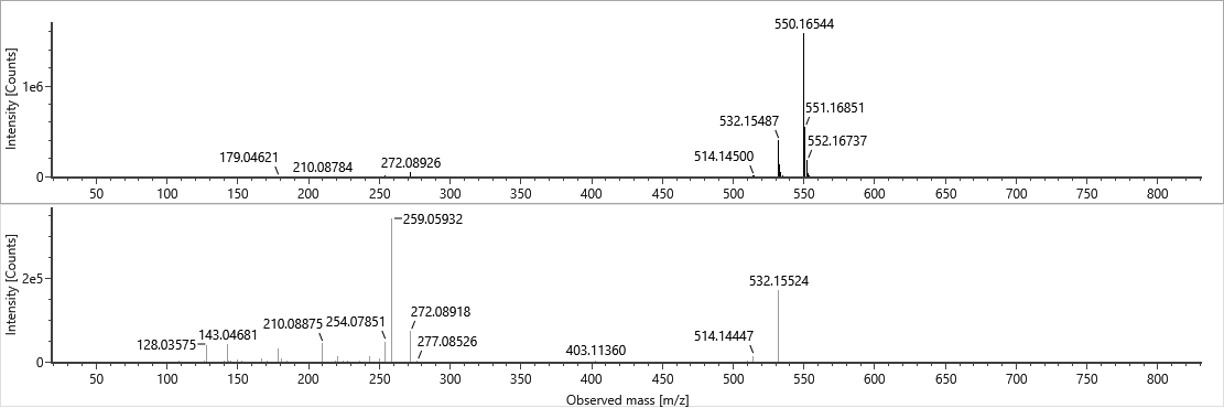

Supplement: Supplementary file 2 — es4c05112_si_002.zip [file es4c05112_si_002.zip › library/png/Benz(a)anthracene glutathione I A.png]

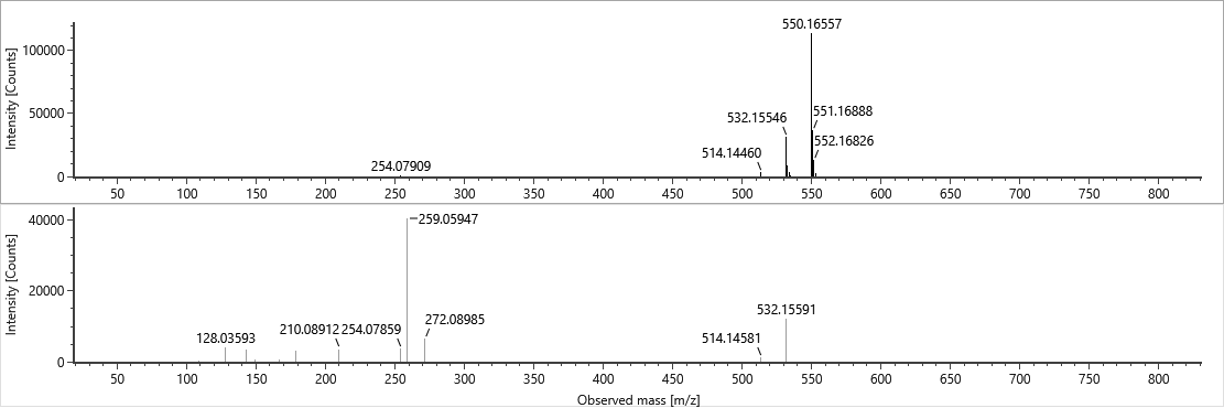

Supplement: Supplementary file 2 — es4c05112_si_002.zip [file es4c05112_si_002.zip › library/png/Benz(a)anthracene glutathione I B.png]

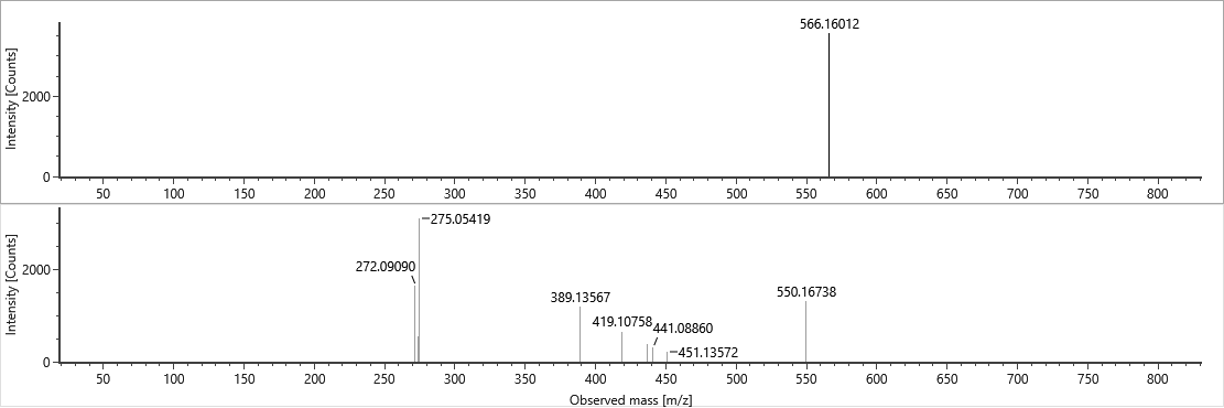

Supplement: Supplementary file 2 — es4c05112_si_002.zip [file es4c05112_si_002.zip › library/png/Benz(a)anthracene glutathione II.png]

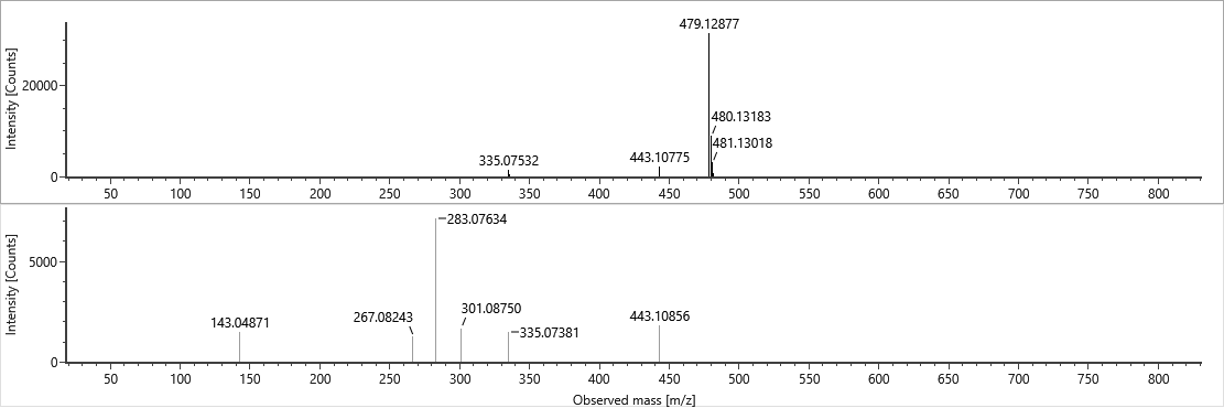

Supplement: Supplementary file 2 — es4c05112_si_002.zip [file es4c05112_si_002.zip › library/png/Benzo(a)pyrene cysteinylglycine A.png]

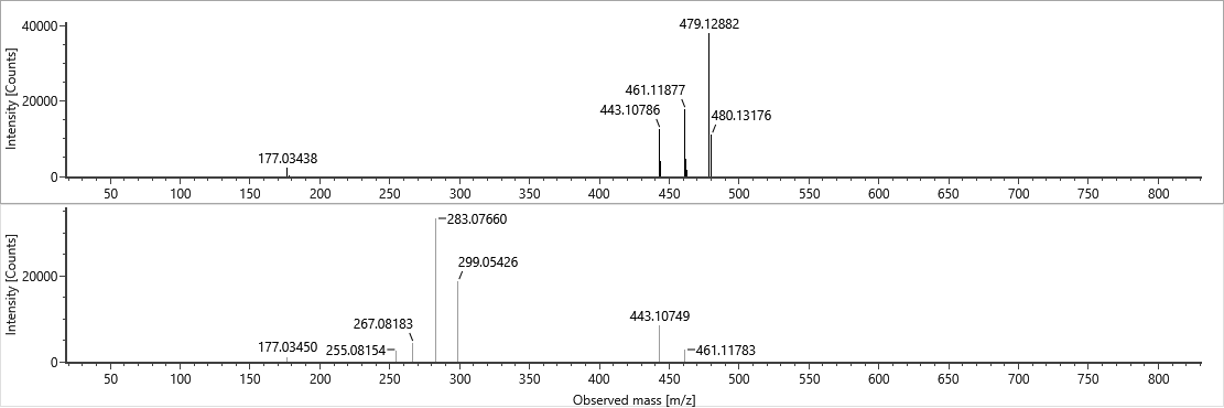

Supplement: Supplementary file 2 — es4c05112_si_002.zip [file es4c05112_si_002.zip › library/png/Benzo(a)pyrene cysteinylglycine B.png]

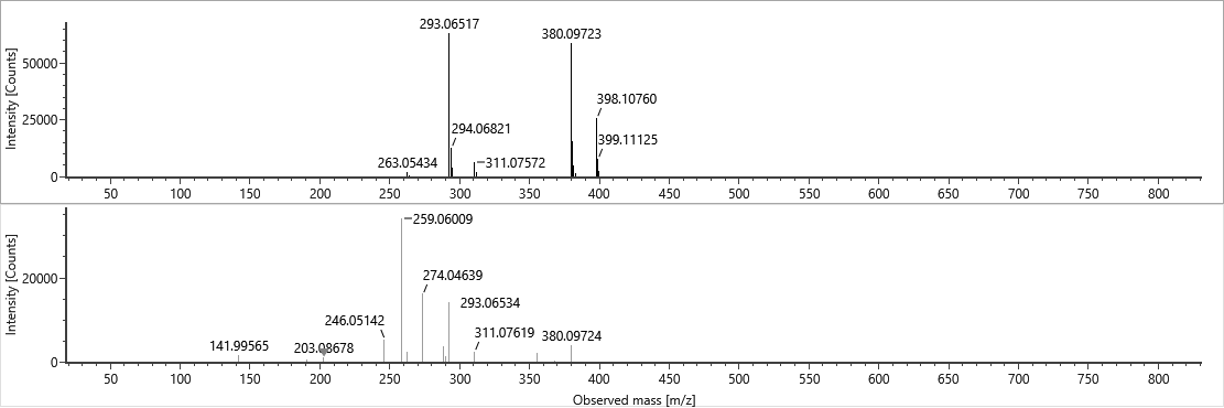

Supplement: Supplementary file 2 — es4c05112_si_002.zip [file es4c05112_si_002.zip › library/png/Chrysene cysteine.png]

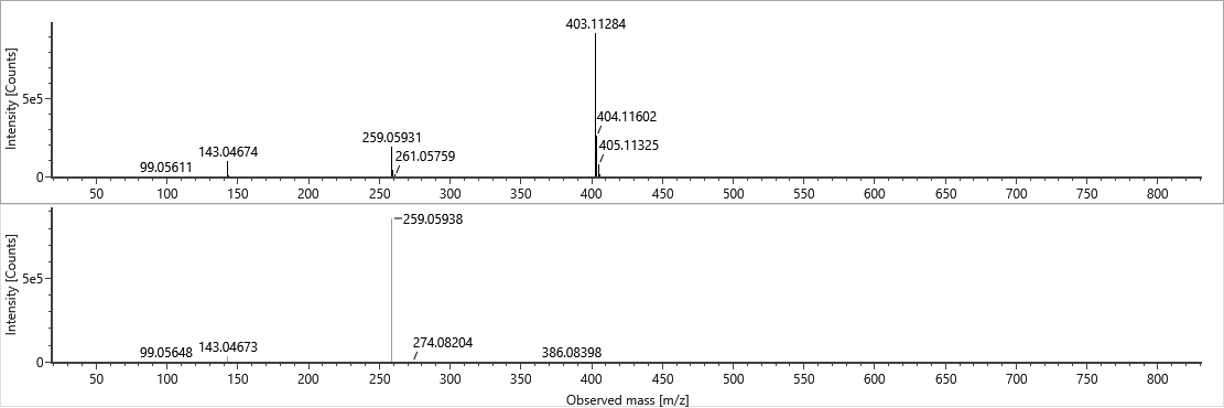

Supplement: Supplementary file 2 — es4c05112_si_002.zip [file es4c05112_si_002.zip › library/png/Chrysene cysteinylglycine I A.png]

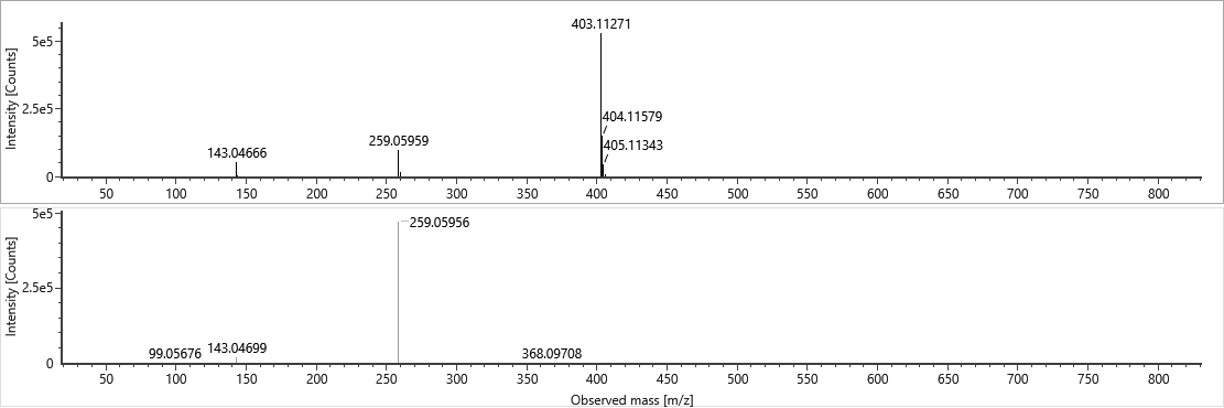

Supplement: Supplementary file 2 — es4c05112_si_002.zip [file es4c05112_si_002.zip › library/png/Chrysene cysteinylglycine I B.png]

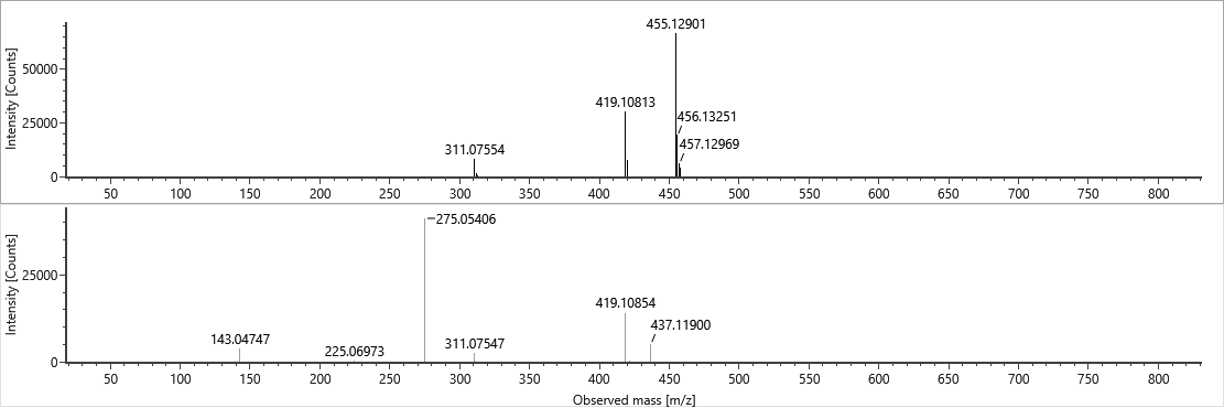

Supplement: Supplementary file 2 — es4c05112_si_002.zip [file es4c05112_si_002.zip › library/png/Chrysene cysteinylglycine II.png]

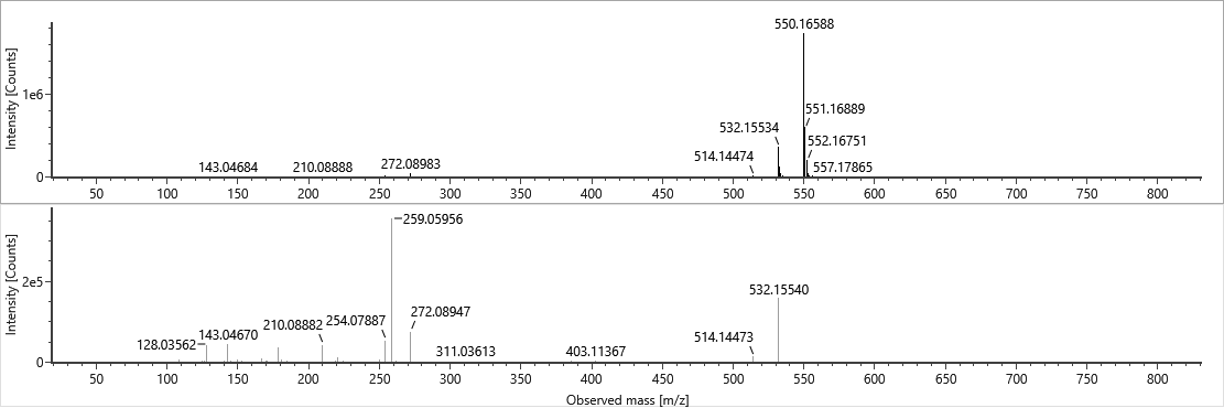

Supplement: Supplementary file 2 — es4c05112_si_002.zip [file es4c05112_si_002.zip › library/png/Chrysene glutathione I A.png]

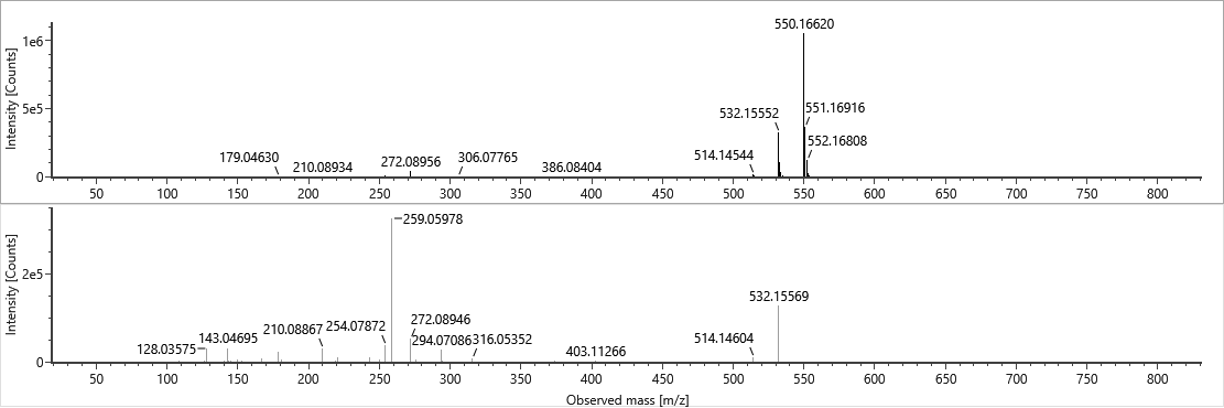

Supplement: Supplementary file 2 — es4c05112_si_002.zip [file es4c05112_si_002.zip › library/png/Chrysene glutathione I B.png]

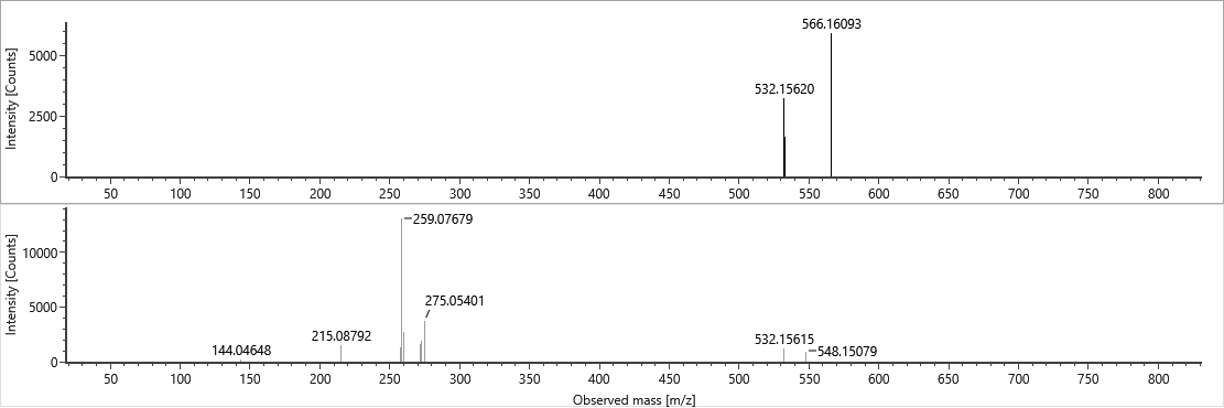

Supplement: Supplementary file 2 — es4c05112_si_002.zip [file es4c05112_si_002.zip › library/png/Chrysene glutathione II.png]

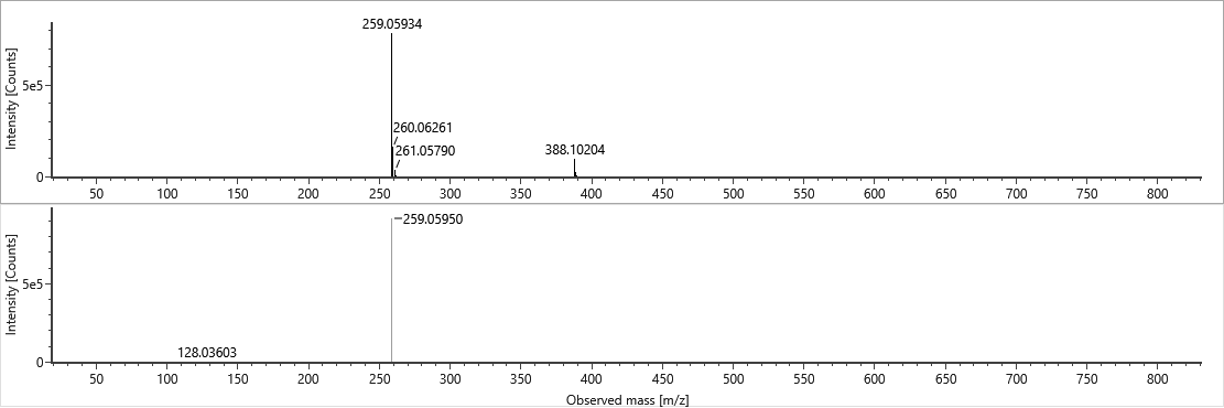

Supplement: Supplementary file 2 — es4c05112_si_002.zip [file es4c05112_si_002.zip › library/png/Chrysene mercapturic acid.png]

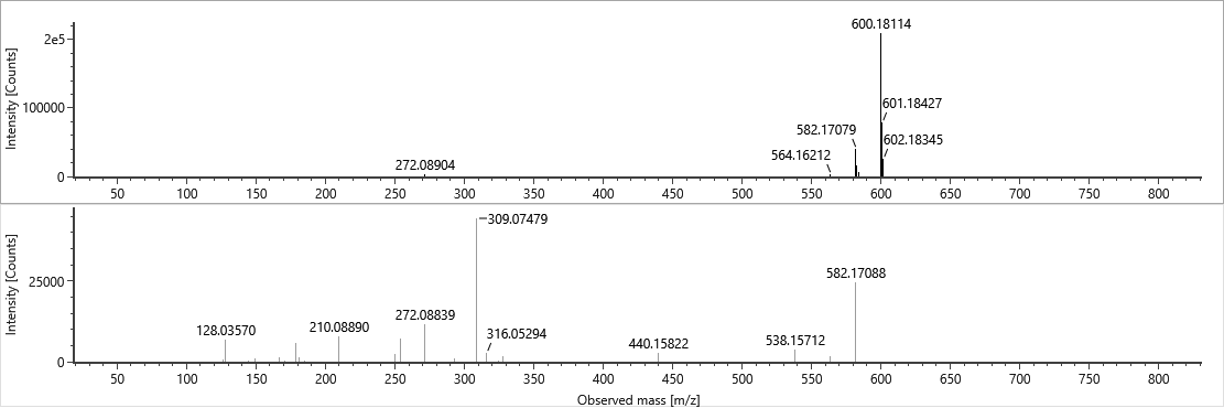

Supplement: Supplementary file 2 — es4c05112_si_002.zip [file es4c05112_si_002.zip › library/png/Dibenz(a,h)anthracene glutathione I A.png]

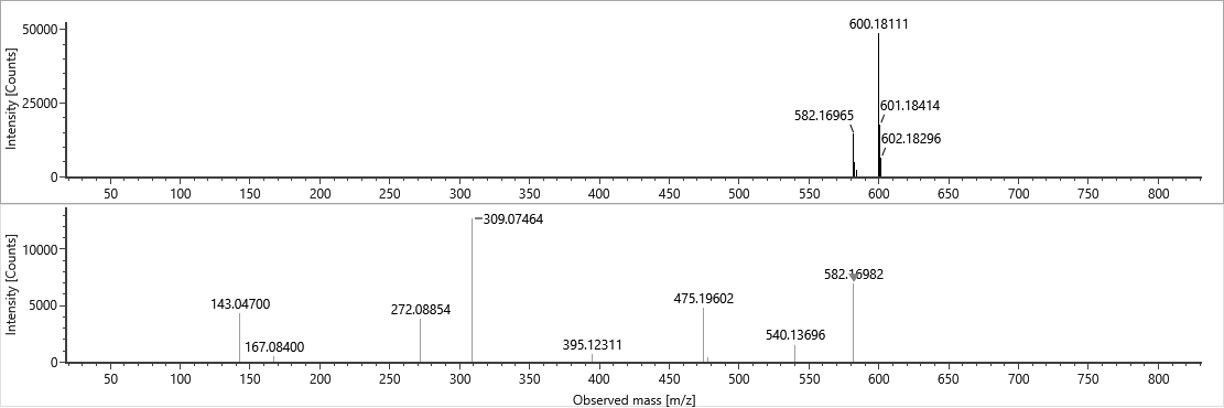

Supplement: Supplementary file 2 — es4c05112_si_002.zip [file es4c05112_si_002.zip › library/png/Dibenz(a,h)anthracene glutathione I B.png]

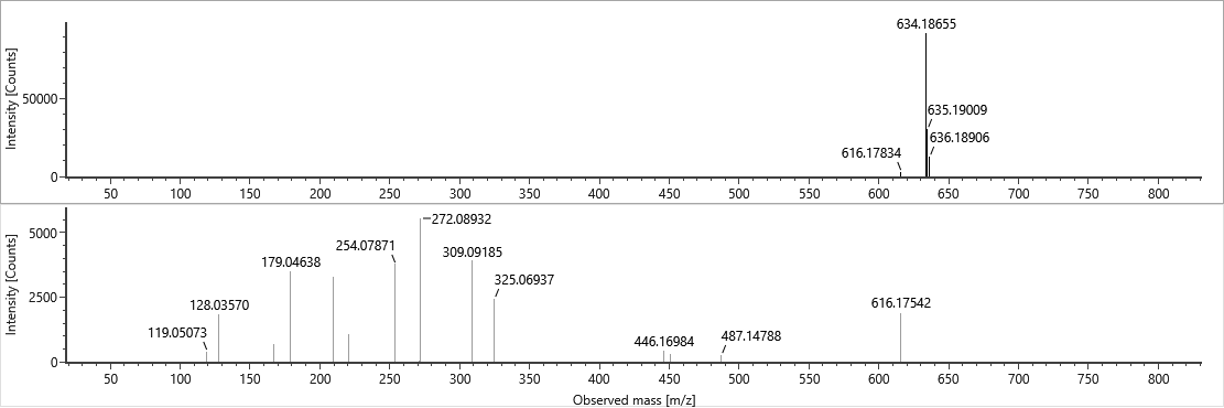

Supplement: Supplementary file 2 — es4c05112_si_002.zip [file es4c05112_si_002.zip › library/png/Dibenz(a,h)anthracene glutathione II.png]

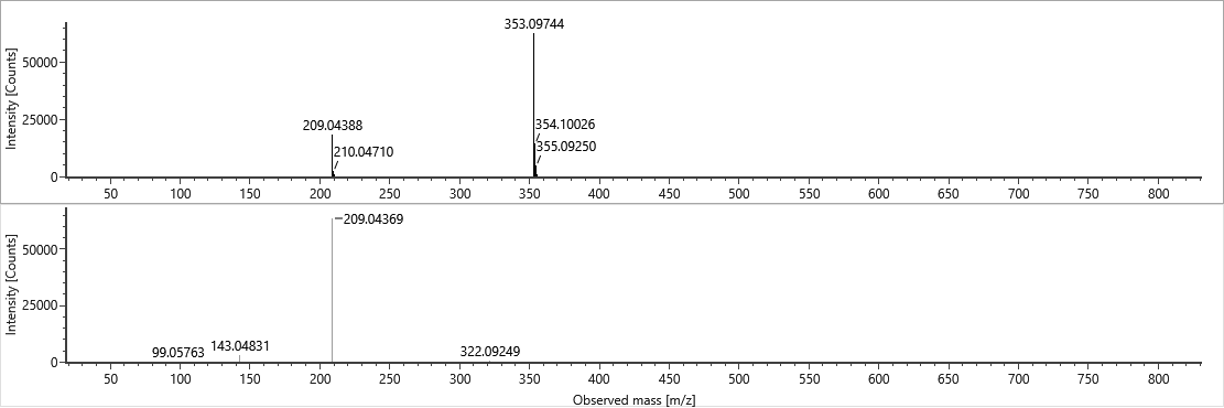

Supplement: Supplementary file 2 — es4c05112_si_002.zip [file es4c05112_si_002.zip › library/png/Phenanthrene cysteinylglycine.png]

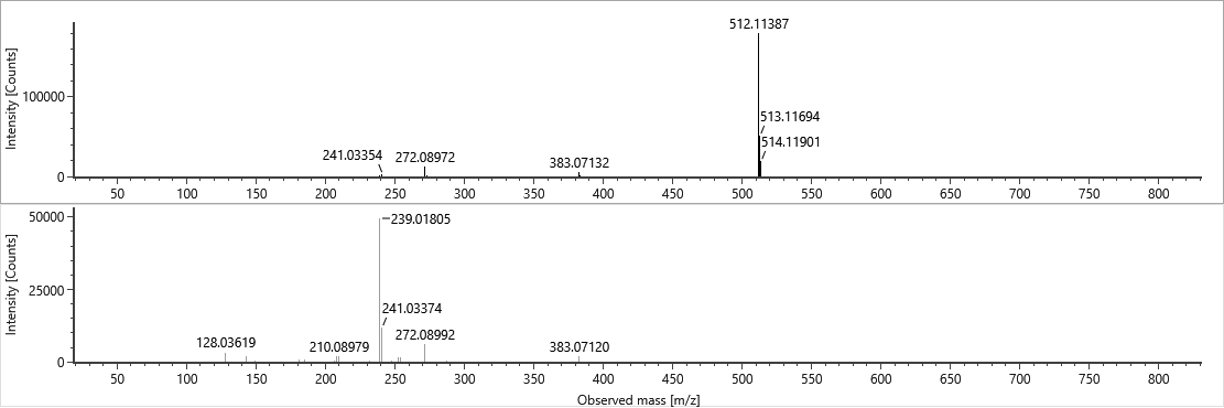

Supplement: Supplementary file 2 — es4c05112_si_002.zip [file es4c05112_si_002.zip › library/png/Phenanthrene glutathione.png]
