# Supplementary material for: Exposure of Polycyclic Aromatic Hydrocarbons (PAHs) and Crude Oil to Atlantic Haddock (Melanogrammus aeglefinus): A Unique Snapshot of the Mercapturic Acid Pathway
Source: Environ Sci Technol. 2024 Aug 5;58(33):14855–63. doi: 10.1021/acs.est.4c05112 (PMC11340023; doi:10.1021/acs.est.4c05112)
Supplement: Supplementary file 3 — es4c05112_si_003.pdf [file es4c05112_si_003.pdf]

Supporting Information

Exposure of polycyclic aromatic hydrocarbons (PAHs) and crude oil to Atlantic haddock (*Melanogrammus aeglefinus*): a unique snapshot of the mercapturic acid pathway

Charlotte L. Nakken[1,2*], Marc H.G. Berntssen[2], Sonnich Meier[2], Lubertus Bijlsma[3], Svein A. Mjøs[1], Elin Sørhus[2], and Carey E. Donald[2]

[1]Department of Chemistry, University of Bergen, Bergen, Norway
[2]Marine Toxicology, Institute of Marine Research, Bergen, Norway
[3]Environmental and Public Health Analytical Chemistry, Research Institute for Pesticides and Water, University Jaume I, Castellón, Spain

*Corresponding author:

e-mail: charlotte.nakken@uib.no

ORCID: https://orcid.org/0000-0002-8335-4909

Summary of the Supporting Information:

4 pages.

2 tables.

The metabolite library is available as a .pdf and .html file (ZIP).

Table S1. Detection results of polycyclic aromatic hydrocarbon (PAH) metabolites from the mercapturic acid pathway (MAP) in fish exposed to single PAH treatments.

Metabolite name	Neutral mass (Da)	Adduct	Expected m/z	Observed m/z	Observed retention time (min)	Observed drift (ms)	Observed collision cross section (CCS) (Å²)
1,4-Dimethylphenanthrene cysteinylglycine A	382.1351	-H	381.1278	381.1284	5.5	6.73	200.21
1,4-Dimethylphenanthrene cysteinylglycine B	382.1351	-H	381.1278	381.1281	7.5	6.50	194.36
1,4-Dimethylphenanthrene glutathione I	511.1777	-H	510.1704	510.1708	7.6	7.42	217.24
1,4-Dimethylphenanthrene glutathione II	529.1883	-H	528.1810	528.1816	5.3	7.51	219.22
1-Methylphenanthrene cysteinylglycine A	368.1195	-H	367.1122	367.1121	5.0	6.39	191.70
1-Methylphenanthrene cysteinylglycine B	368.1195	-H	367.1122	367.1127	8.5	6.40	192.08
1-Methylphenanthrene glutathione I	497.1621	-H	496.1548	496.1547	5.8	7.27	213.51
1-Methylphenanthrene glutathione II	515.1726	-H	514.1653	514.1657	2.2	7.46	218.20
Anthracene cysteinylglycine	354.1038	-H	353.0965	353.0972	2.5	6.27	189.08
Benz(a)anthracene cysteinylglycine I A	404.1195	-H	403.1122	403.1123	6.2	6.86	203.38
Benz(a)anthracene cysteinylglycine I B	404.1195	-H	403.1122	403.1122	5.9	6.92	204.83
Benz(a)anthracene cysteinylglycine II	422.1300	-H	421.1228	421.1230	4.4	6.89	203.92
Benz(a)anthracene cysteinylglycine III	438.1249	-H	437.1177	437.1181	2.2	6.60	196.72
Benz(a)anthracene cysteinylglycine IV A	456.1355	-H	455.1282	455.1289	2.8	7.08	208.64
Benz(a)anthracene cysteinylglycine IV B	456.1355	-H	455.1282	455.1283	3.7	7.10	209.13
Benz(a)anthracene glutathione I A	551.1726	-H	550.1653	550.1654	3.2	8.03	232.77
Benz(a)anthracene glutathione I B	551.1726	-H	550.1653	550.1656	2.5	7.81	227.05
Benz(a)anthracene glutathione II	567.1675	-H	566.1603	566.1601	2.2	8.15	235.78
Benzo(a)pyrene cysteinylglycine A	480.1355	-H	479.1282	479.1288	4.2	7.21	211.86
Benzo(a)pyrene cysteinylglycine B	480.1355	-H	479.1282	479.1288	5.2	7.28	213.66
Chrysene cysteine	399.1140	-H	398.1068	398.1076	4.1	6.49	194.41
Chrysene cysteinylglycine I A	404.1195	-H	403.1122	403.1128	4.8	6.81	202.05

Chrysene cysteinylglycine I B	404.1195	-H	403.1122	403.1127	5.8	6.92	204.83
Chrysene cysteinylglycine II	456.1355	-H	455.1282	455.1290	3.9	7.04	208.01
Chrysene glutathione I A	551.1726	-H	550.1653	550.1659	2.3	7.95	230.76
Chrysene glutathione I B	551.1726	-H	550.1653	550.1662	2.9	8.03	232.71
Chrysene glutathione II	567.1675	-H	566.1603	566.1609	1.5	7.58	221.02
Chrysene mercapturic acid	389.1086	-H	388.1013	388.1020	4.5	6.89	204.22
Dibenz(a,h)anthracene glutathione I A	601.1883	-H	600.1810	600.1811	5.8	8.43	243.18
Dibenz(a,h)anthracene glutathione I B	601.1883	-H	600.1810	600.1811	6.7	8.63	248.13
Dibenz(a,h)anthracene glutathione II	635.1938	-H	634.1865	634.1866	3.7	8.63	248.58
Phenanthrene cysteinylglycine	354.1038	-H	353.0965	353.0974	2.1	6.30	189.75
Phenanthrene glutathione	513.1206	-H	512.1133	512.1139	2.9	7.24	212.65

Table S2. Detection results of polycyclic aromatic hydrocarbon (PAH) metabolites from the mercapturic acid pathway (MAP) in fish exposed to crude oil.

PAH type	Observed m/z	Mass error (ppm)	Observed CCS (Å²)	Observed retention time (min)	Diagnostic common neutral losses found (within 5mDa tolerance)	Diagnostic common fragment ions found (*m/z*) (within 5mDa tolerance)
Dimethylated 3-ring cysteinylglycine	381.1277	-0.5	198.68	4.6	144.05349 (Cysteinylglycine)	143.04622 (Cysteinylglycine)
4-ring cysteinylglycine	403.1124	0.6	200.28	4.8	144.05349 (Cysteinylglycine)	143.04622 (Cysteinylglycine)
Methylated 3-ring glutathione	496.1547	-0.2	211.05	4.9	273.09609 (Glutathione)	272.08881 (Glutathione)
Dimethylated 3-ring glutathione A	510.1708	0.6	217.26	6.6	273.09609 (Glutathione)	272.08881 (Glutathione)
Dimethylated 3-ring glutathione B	510.1703	-0.3	220.21	7.1	273.09609 (Glutathione)	272.08881 (Glutathione)
Methylated 3-ring cysteinylglycine	367.1117	-1.3	192.42	9.3	144.05349 (Cysteinylglycine)	143.04622 (Cysteinylglycine)
